# Effect of lysine side chain length on histone lysine acetyltransferase catalysis

**DOI:** 10.1038/s41598-020-69510-0

**Published:** 2020-08-03

**Authors:** Giordano Proietti, Yali Wang, Giorgio Rainone, Jasmin Mecinović

**Affiliations:** 1 Department of Physics, Chemistry and Pharmacy, University of Southern Denmark, Campusvej 55, 5230 Odense, Denmark; 2 Institute for Molecules and Materials, Radboud University, Heyendaalseweg 135, 6525 AJ Nijmegen, The Netherlands; 3 Department of Blood Transfusion, China-Japan Union Hospital, Jilin University, 126 Xiantai Street, Changchun, 130033 People’s Republic of China

**Keywords:** Chemical tools, Enzymes, Peptides, Post-translational modifications

## Abstract

Histone lysine acetyltransferase (KAT)-catalyzed acetylation of lysine residues in histone tails plays a key role in regulating gene expression in eukaryotes. Here, we examined the role of lysine side chain length in the catalytic activity of human KATs by incorporating shorter and longer lysine analogs into synthetic histone H3 and H4 peptides. The enzymatic activity of MOF, PCAF and GCN5 acetyltransferases towards histone peptides bearing lysine analogs was evaluated using MALDI-TOF MS assays. Our results demonstrate that human KAT enzymes have an ability to catalyze an efficient acetylation of longer lysine analogs, whereas shorter lysine analogs are not substrates for KATs. Kinetics analyses showed that lysine is a superior KAT substrate to its analogs with altered chain length, implying that lysine has an optimal chain length for KAT-catalyzed acetylation reaction.

## Introduction

**Figure 1:**
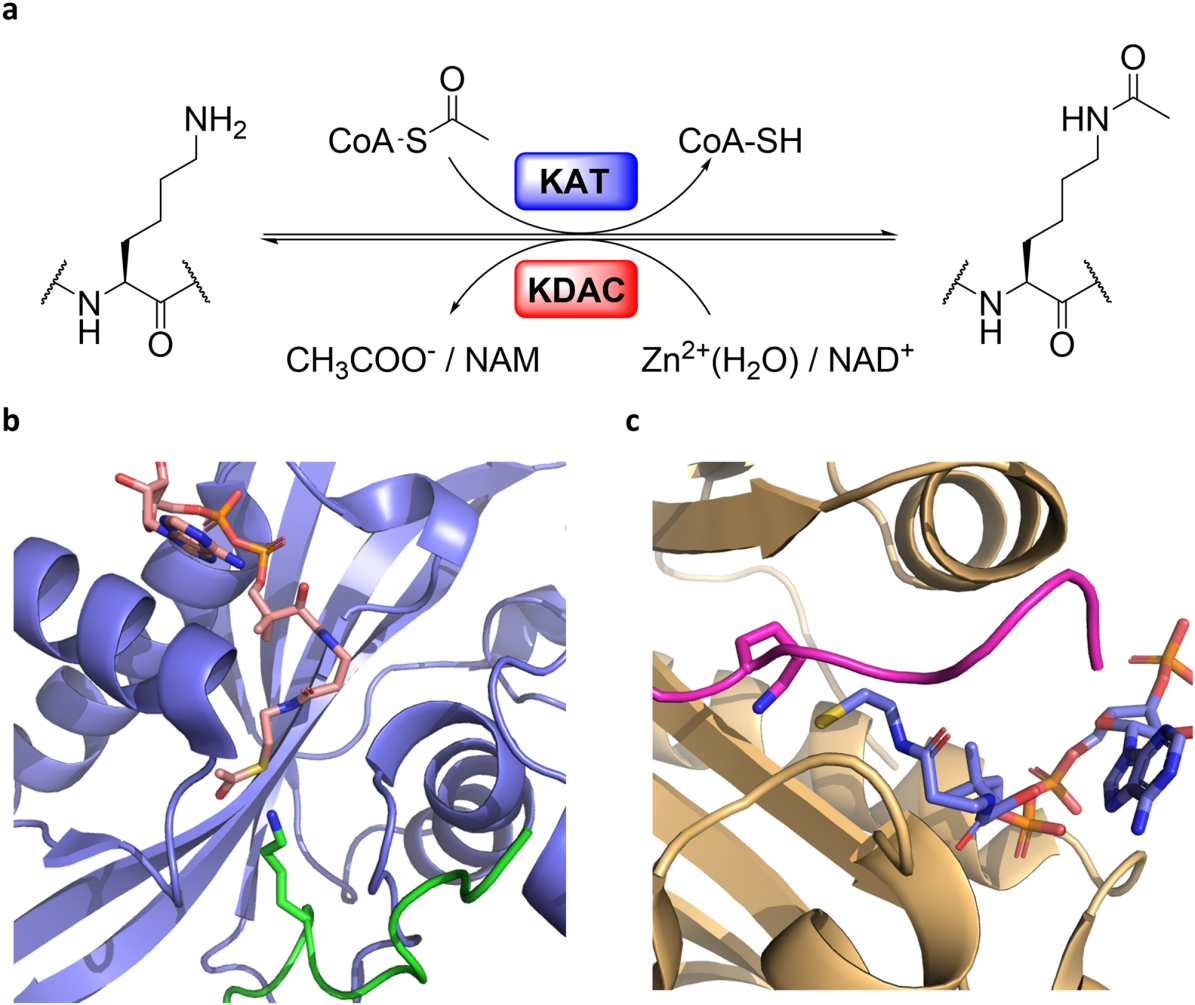
(**a**) KAT-catalyzed histone lysine acetylation and KDAC-catalyzed histone lysine deacetylation; (**b**) View on HAT1 acetyltransferase (blue) complexed with AcCoA (pink) and H4K12 (green) (PDB: 2P0W); (**c**) View on tGCN5 acetyltransferase (yellow) complexed with CoASH (blue) and H3K14 (magenta) (PDB: 1QSN).

Posttranslational modifications (PTMs) occurring on histone proteins are reckoned to play a key role in several biological processes, including gene expression, DNA repair and replication^1,2^. The arrangement of chemical modification patterns present on flexible histone tails has been postulated to constitute the “Histone Code,” a molecular handbook that is utilized by transcriptional factors to selectively express one gene over the estimated twenty thousand others encoded within the human genome^2–4^. The panorama of histone PTMs is wide, and new PTMs are continuously emerging^5–8^. Among the multitude of PTMs discovered, lysine acetylation appears to be one of the most widespread^9^. The installation of the acetyl group from acetyl Coenzyme A (AcCoA) cosubstrate onto the N^Ɛ^-amino group of lysine residues in histones is catalyzed by histone lysine acetyltransferases (KATs) (Fig. 1a) ^10,11^. Histone lysine acetylation is correlated with the genome accessibility and transcription enhancement by influencing the dynamic equilibrium that regulates chromatin packing^12^. The neutralization of the lysine’s positive charge, which occurs upon the acetyl transfer, allows chromatin to adopt a more loose and transcriptionally accessible euchromatin state^12,13^. To guarantee a precise regulation of lysine acetylation, histones acetylome profile is further tuned by the activity of histone lysine deacetylases (KDACs) that catalyze the removal of the lysine’s acetyl moiety (Fig. 1a) ^14^, whereas recognition of acetylated lysine residues on histone tails by bromodomains participates in the recruitment of transcription factors^15,16^.

KATs are comprised of a large family of proteins that can be categorized in base of their cellular localization and homology of sequences^17^. The nuclear KATs—type A—are responsible for the acetylation of histones and several non-histone proteins in the nucleus, regulating the activity of many oncoproteins, including p53^18^. As a prerogative of their catalytic mechanism that requires AcCoA as the acetyl donor, most of KATs share a highly conserved 15–33 amino acids pattern (motif a: R*/*Q-X-X-G-X-G*/*A), which is essential for the accommodation of the AcCoA cosubstrate^19^. On the other hand, the presence of different auxiliary domains, among which chromo- and bromodomains, provides KATs with specific substrate preferences and the possibility of interaction with unique binding partners, to form multi-subunit complexes^17,19^. Under this light it is possible to further divide type A KATs in three big subfamilies: the GNAT (Gcn5 related *N*-acetyltransferase), the MYST (MOZ, Ybf2, Sas2 and Tat-interacting protein) and the p300/CBP families^17^. Outside the beforementioned phylogenetic tree, TAF1/TBP (belonging to the components of transcription factors complexes family), SRC1 and CLOCK (belonging to the nuclear receptors coactivator family), the fungal Rtt109 and αTAT1 (accountable for the acetylation of α-tubulin) are among the most well studied type A KATs^17^. Even though lysine’s nucleophilic character in the catalytic site is triggered by a water-mediated deprotonation initiated by a glutamic acid residue, which is highly conserved among the GNAT and MYST families, or aspartic acid in the case of p300, to date different catalytic mechanisms have been proposed among KATs^20,21^. The GNAT family members were found to catalyze a direct acetyl transfer from the AcCoA cosubstrate to the nucleophilic N^Ɛ^-amino group of lysine upon constitution of a ternary complex comprising of KAT-AcCoA-Histone^20,21^. Members of the MYST family are instead reckoned to catalyze acetylation of histone substrates through a ping-pong mechanism. Combined crystallographic, kinetics, mutagenesis and thermodynamic evidences support that the acetyl transfer mechanism in MYST enzymes is mediated via the formation of the S-acetylated cysteine intermediate in the catalytic pocket that upon reaction with lysine produces the acetyllysine product^22–24^. Although several KAT crystal structures have been determined, only a few examples include ternary complexes with both histone substrates and AcCoA cosubstrate (or CoASH product), contributing to a limited understanding of KATs’ catalytic properties (PDB ID: 2P0W and 1QSN, Fig. 1b,c).

Due to their indisputable importance in tuning gene expression, not surprisingly many KATs have been found overexpressed and/or dysregulated in several human pathologies, such as cancer, inflammation, and neurodegenerative disorders^25–28^. In the past decade, several inhibitors targeting this biomedically important family of epigenetic enzymes have been developed, many of them lacking inter and intra families selectivity as well as displaying poor activity^29,30^. Given the challenges encountered in the development of KATs inhibitors, a better understanding of the substrate scope for KAT catalysis is of great biomolecular and medicinal interest. Exploring the substrate and cosubstrate specificity of KAT-catalyzed reactions is an important tool that can provide valuable information for the rational design of new inhibitors^31^. Towards this aim, extensive work has been carried out to unravel the specificity of the AcCoA cosubstrate in KAT catalysis^32,33^. KATs have been found to catalyze the transfer of sterically more demanding acyl moieties, including propionyl, crotonyl, and butyryl, both in vivo and in vitro, from their respective acyl CoA cosubstrate, with differences in cosubstrate acceptance among families^7, 8, 34–36^. The promiscuity of AcCoA led to the development of several chemical probes for KATs as well as a click-based labelling procedure for the detection of KATs substrates, employing synthetic CoA surrogates and engineered KATs^37–39^. Although in the recent years the investigation of epigenetic enzymes’ susceptibility towards lysine chemical modification gathered great attentions, especially with regards to histone lysine methyltransferases (KMTs), histone lysine demethylases (KDMs) and KDACs, we are currently lacking basic molecular knowledge in the context of histone lysine acetylation^40–47^. Enzyme kinetics showed p300 catalytic efficiency towards a collection of Lys analogs; a synthetic H4K8 peptide was found to be the preferred substrate, followed by D-Lys and γ-thiahomolysine that showed very poor catalytic efficiency^48^. More recently, γ-thialysine was demonstrated to be efficiently accepted by a panel of human KATs for the transfer of acetyl, propionyl and butyryl moieties from their respective acyl CoA cosubstrates^49^. To provide a better insight into the role of lysine side chain on histone acetylation, this study is aimed at understanding of the relevance of lysine side chain length on human KAT catalysis.

## Results and Discussion

**Figure 2:**
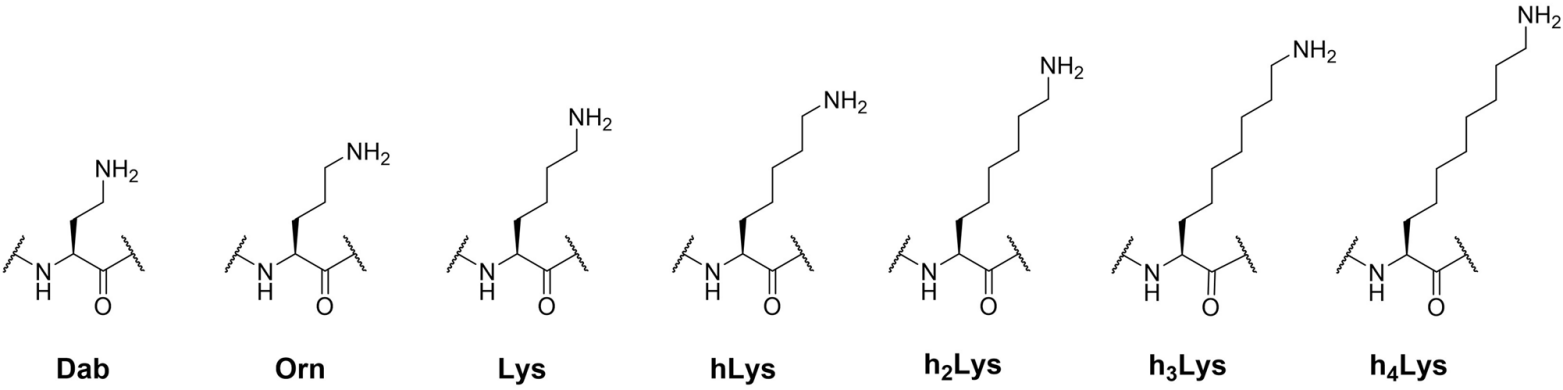
A panel of lysine analogs possessing a different side chain length.

**Figure 3:**
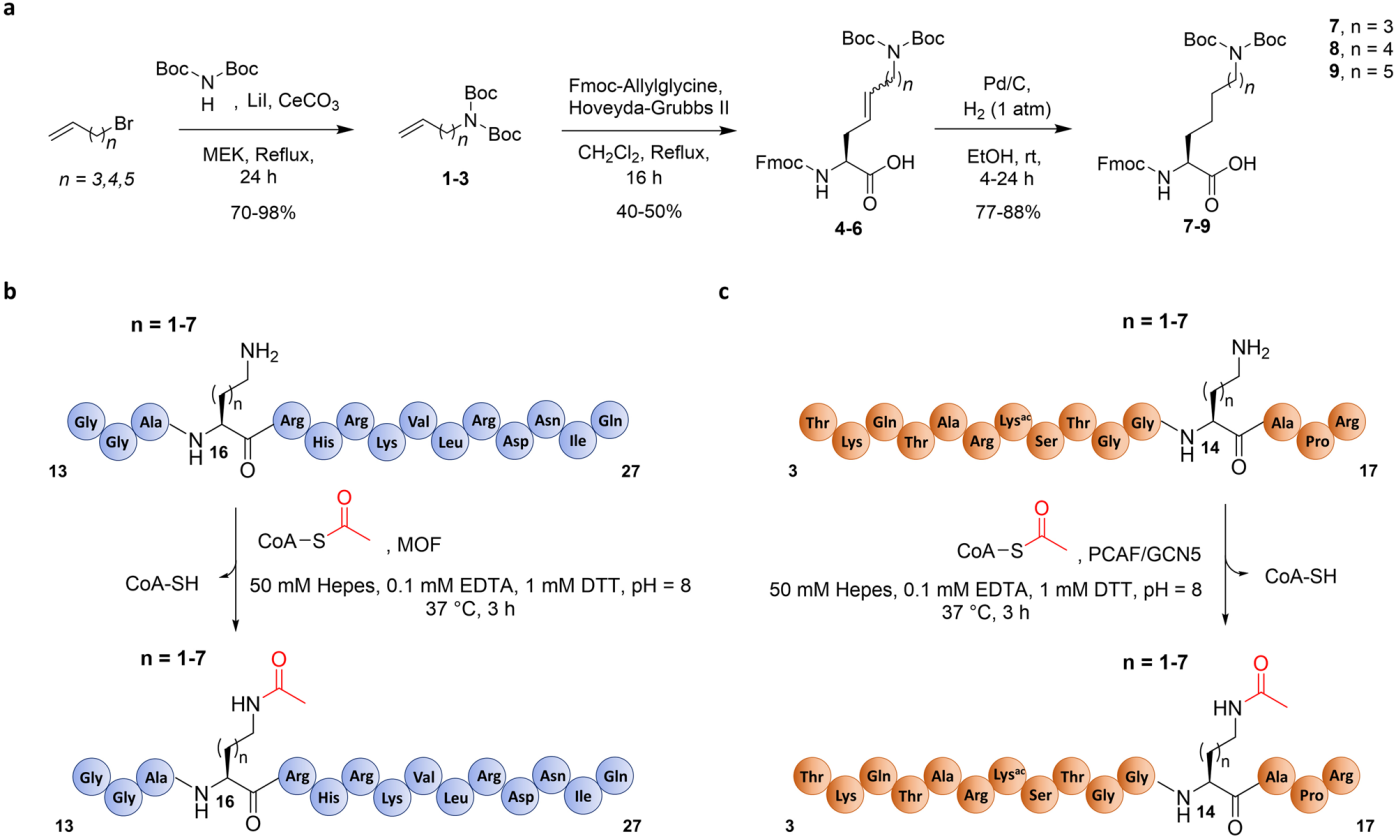
Synthesis and enzymatic evaluation of lysine analogs with altered chain length. (**a**) Synthetic scheme for the preparation of lysine analogs with longer chain length; (**b**) Graphic representation of AcCoA-mediated acetylation of H4 peptides by MOF. (**c**) Graphic representation of AcCoA-mediated acetylation of H3 peptides by PCAF and GCN5.

To investigate the role of the length of lysine’s side chain on human KAT catalysis, a panel of histone peptides bearing lysine analogs that differ in the chain length was developed via Fmoc-based Solid-Phase Peptide Synthesis (SPPS) (Fig. 2). While several unnatural lysine analogs with shorter and longer side chain are commercially available (Dab = two carbons; Orn = three carbons; Lys; hLys = five carbons), novel longer analogs were synthesized in three steps employing a cross-metathesis synthetic strategy (Fig. 3a). Hoveyda-Grubbs II generation catalyst was employed to perform the coupling of Fmoc-allylglycine with synthetic diBoc-protected amino alkenes of variable alkyl chain lengths. The resulting cis/trans mixture was subsequently reduced by hydrogenation in the presence of Pd/C to yield the desired Fmoc-protected lysine analogs (Fig. 3a). Three different human KATs were selected and expressed in *E. coli*: (i) MOF (KAT8), a member of the MYST family, which preferentially catalyzes acetylation of K16 on the histone H4 tail; and (ii) GCN5 (KAT2A) and PCAF (KAT2B), members of the GNAT family, which predominantly catalyze acetylation of K14 and secondarily of K9 on the histone H3 tail^50,51^. To obtain interpretable data, we decided to exclude secondary sites of acetylation from the histone H3 peptide sequence. Therefore, we employed a truncated 15-mer H4 peptide (residues 13–27, sequence: GGA**K**_**16**_RHRKVLRDNIQ) as a reference sequence to study MOF-catalyzed acetylation of H4K16, and two different 15-mer H3 peptides, with alternatively synthetically acetylated K9 and K14 on H3 peptide, to investigate the acetylation of both sites by GCN5 and PCAF (residues 3–17, sequence: TKQTARKacSTGG**K**_**14**_APR; residues 1–15, sequence: ARTKQTAR**K**_**9**_STGGKacA). Fmoc-protected lysine analogs containing a modified chain length were incorporated into the key positions of H3 and H4 peptides by SPPS (Fig. 3b,c). Synthetic histone peptides were purified by RP-HPLC, and their identity and purity were assessed by ESI–MS and analytical HPLC (Supplementary Table 1 and Supplementary Figs. 1–24).

**Figure 4:**
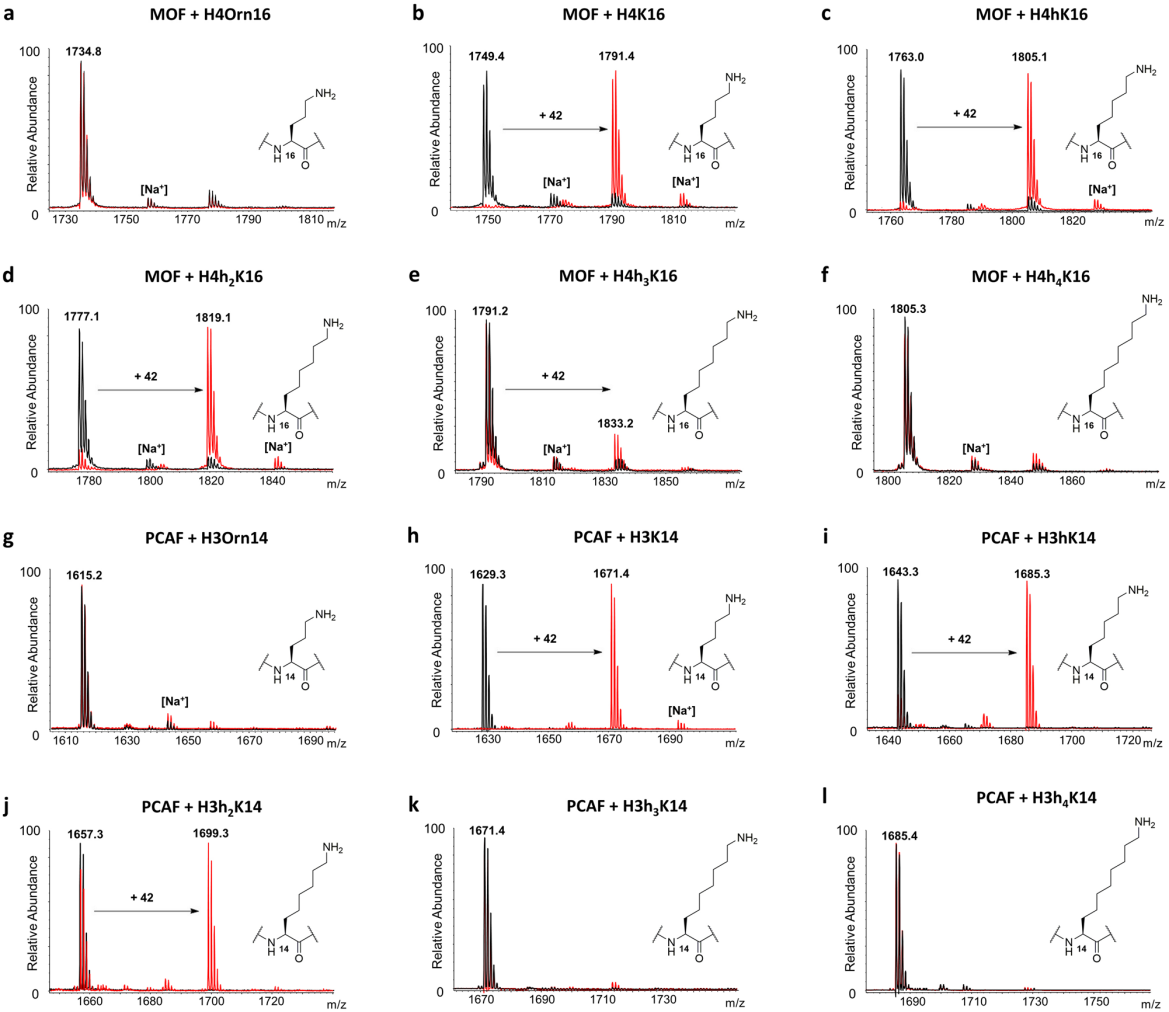
MALDI-TOF MS data showing (**a**–**f**) MOF (2 μM)-catalyzed acetylation of H4K*16 histone peptides (100 μM) and (**g**–**l**) PCAF (2 μM)-catalyzed acetylation of H3K*14 histone peptides (100 μM) containing lysine analogs in the presence of AcCoA cosubstrate (300 μM); Overlaid MS spectra of KAT-catalyzed reactions (red) and no enzyme control reactions (black) that were quenched after 3 h incubation at 37 °C.

MALDI-TOF MS enzymatic assays were carried out to investigate KAT-catalyzed acetylation of synthetic histone peptides. The enzymatic activity of the recombinantly expressed human KATs was measured at different time points (2 µM KAT enzyme, 100 µM histone peptide, 300 µM AcCoA, buffer: 50 mM Hepes, 0.1 mM EDTA, 1 mM DTT, pH = 8.0, 37 °C, hereafter used as standard conditions) (Fig. 3b,c). MOF catalyzed full acetylation of H4K16 in 30 min, whereas GCN5 and PCAF catalyzed quantitative acetylation of H3K14 in 15 min and H3K9 only after 2–3 h (Fig. 4b, h and Supplementary Figs. 25–28). It is noteworthy that diacetylation of histone peptides was not detected under our assay conditions, due to preacetylation of the secondary site on H3. Two control experiments in the absence of KAT and AcCoA showed no acetylation within detection limits, confirming the dependence of the acetylation reaction on the KAT enzymes and AcCoA (Supplementary Figs. 26–28). As further control, H4 and H3 peptides containing stereochemically inverted D-lysine were synthesized. MALDI-TOF data revealed that histones bearing D-lysine are not substrates for our panel of KAT enzymes under standard conditions (Supplementary Figs. 29–33). These findings demonstrate that the lysine’s L-stereochemistry is an essential chemical prerequisite for MOF, GCN5 and PCAF catalysis. Interestingly, p300, one of the most promiscuous KATs, has been observed to catalyze acetylation of the H4K8 histone peptide containing D-Lys (D-H4K8)^48^. Kinetics data showed that D-H4K8 has a very poor catalytic efficiency relative to H4K8 (76-fold drop in V/K with respect to the H4K8 peptide), a result that may lay in the marked structural differences between p300 and the other KAT families^19^. In a broader context, D-Lys has been found to be a very poor substrate for functionally related human KMTs, where traces of methylation were detected occasionally upon increased reaction times and enzyme concentrations^42^.

We next examined the capacity of human KAT enzymes to catalyze acetylation of unnatural lysine analogs under standard conditions. Histone peptides bearing shorter lysine analogs, Dab and Orn, did not undergo acetylation under standard conditions with MOF, PCAF and GCN5, at primary and secondary sites of acetylation (Fig. 4a,g and Supplementary Figs. 34–41). In contrast, we observed KAT-catalyzed acetylation of longer side chain analogs (hK, h_2_K, h_3_K), though in different degrees among the three enzymes. MOF-catalyzed acetylation of H4hK16 and H4h_2_K16 was found to be almost quantitative (95% and 90%) after 3 h (Fig. 4c,d). On the other hand, GCN5/PCAF catalytic activity towards the same analogs was generally found reduced. Acetylation of the H3hK14 peptide led to a formation of 83% of H3hK14ac by PCAF and 62% of H3hK14ac by GCN5 after 3 h (Fig. 4i, Supplementary Fig. 42), whereas the H3h_2_K14 peptide was found to be less efficiently acetylated after 3 h (55% by PCAF and 25% by GCN5) (Fig. 4j, Supplementary Fig. 43). Following the same pattern shown for H3K14, the unnatural analogs introduced at position 9 of H3 underwent less efficient acetylation in the presence of PCAF and GCN5. After 3 h, PCAF and GCN5 were only able to catalyze partial acetylation of H3hK9, producing 50% and 30% of H3hK9ac, respectively (Supplementary Figs. 44–45), whereas the H3h_2_K9 peptide was acetylated to a lesser degree (33% and 14%; Supplementary Figs. 46–47). For all three KATs, h_3_K-containing histone peptides were observed to be very poor substrates, with conversion as low as 12% for MOF after 3 h (Fig. 4e), and traces (< 5%) for PCAF (Fig. 4k) and GCN5 at H3K14 (Supplementary Fig. 48). No acetylation was observed within limits of detection for the H3h_3_K9 peptide in the presence of PCAF and GCN5 (Supplementary Figs. 49–50). Interestingly, no acetylation of h_4_K-containing peptides was detected either with MOF (Fig. 4f), PCAF (Fig. 4l and Supplementary Fig. 51) or GCN5 (Supplementary Figs. 52–53) on H4 and H3, establishing the maximal lysine side chain length to be suitable for KAT catalysis to seven carbons. Our results on KAT-catalyzed acetylation of lysine analogs possessing extended side chains provide an interesting finding. Firstly, to date the only longer lysine analog found to be acetylated by p300 has been γ-thiahomolysine, which differs from the fully carbon-based homologous hLys in basicity, side-chain angle and bond length. Secondly, the extremely stringent chain length requirements regulating the activity of other lysine-modifying epigenetic enzymes, such as KMTs and KDACs, are not a prerequisite for KAT enzymes^42,46^. In this context, trimethyllysine-binding epigenetic reader proteins appear to tolerate the alterations of the side chain length to a higher degree than the enzymes that install the methyl group on lysine^52^.

To further assess MOF, GCN5 and PCAF substrate specificity towards our library of histone peptides possessing lysine and its longer side chain analogs, MALDI-based kinetics investigations were carried out under steady-state conditions. Kinetic analysis of bi-substrate enzymes, such as KATs, is reckoned to be challenging and dependent on many factors. Firstly, given the nature of these enzymes, the binding of one of the two substrates directly influences the binding of the other (with a direct effect on the Michaelis–Menten parameter K_m_). Secondly, as suggested by Wapenaar and Dekker, the catalytic mechanism of KATs seems to be dependent on the nature of the enzyme construct (whether catalytic domain, full-length or belonging to multi-subunit complexes), as in the case for ESA1, which displayed a ternary complex mechanism when integrated in the piccolo NuA4 complex, and the characteristic cysteine mediated ping-pong mechanism when ESA1 catalytic domain alone was employed^24,53,54^. Therefore, to obtain data that would allow us to directly compare the catalytic properties of the selected KATs towards our selection of histone peptides bearing lysine analogs with longer side chains, two strategies to standardize our kinetic evaluation were taken: i) all the experiments were carried out with the same saturating concentrations of AcCoA and ii) only KATs catalytic domains were employed in the study. Overall, the unnatural lysine analogs exhibited a decreased catalytic activity when compared to their natural counterparts, with decreasing k_cat_ values at the insertion of any additional carbon on lysine side chain (Table 1). Evaluation of MOF kinetic properties revealed that lysine is approximately a 50 times better substrate than its side-chain extended analogs (Table 1). In the case of the H4hK16 peptide, the drop in the catalytic efficiency (k_cat_/K_m_) can be attributable to the only decrease of k_cat_ values, due to the display of comparable K_m_ values with H4K16. Interestingly, H4h_2_K16 is a comparable substrate to H4hK16, with very similar k_cat_/K_m_ values. The result seems to not depend on the number of turnovers of MOF, which are found decreased, in line with the general trend, but rather because of H4h_2_K16′s more favourable binding affinity – as reflected in fourfold lower K_m_ values. PCAF and GCN5 kinetic analysis revealed that K_m_ values generally remained conserved among the peptides’ series, implying a similar binding affinity (Table 1). In contrast, k_cat_ values decreased substantially with an increase of the side chain length, both in the K14 and K9 peptide series, and for both PCAF and GCN5 (Table 1). This finding, combined with the conserved K_m_ values, revealed that the resulting drops in k_cat_/K_m_ values towards the side chain extended lysine analogs were mainly a result of a reduced turnover rate. Inserting one carbon (H3hK14) and two carbons (H3h_2_K14) into the lysine side chain caused k_cat_/K_m_ decreases of 354-and 890-fold for GCN5, and 263- and 625-fold for PCAF, compared to the acetylation of the H3K14 sequence.

**Table 1:** Kinetics parameters for KAT-catalyzed acetylation of H4K*16, H3K*14 and H3K*9 histone peptides.

Enzyme	Peptide	k_cat_^*app*^ (min^−1^)	K_m_^*app*^ (μM)	k_cat_^*app*^/K_m_^*app*^ (mM^−1^ min^−1^)
MOF	H4K16	23.10 ± 1.0	593.3 ± 86	38.9
	H4hK16	0.27 ± 0.1	343.7 ± 99	0.8
	H4h_2_K16	0.06 ± 0.01	82.2 ± 15	0.7
PCAF	H3K14	364 ± 1.2	727 ± 95	500
	H3hK14	0.93 ± 0.2	487.6 ± 98	1.9
	H3h_2_K14	0.5 ± 0.1	606.1 ± 96	0.8
	H3K9	2.75 ± 0.2	235.0 ± 35	11.7
	H3hK9	0.21 ± 0.01	269.6 ± 54	0.8
	H3h_2_K9	0.07 ± 0.01	179.0 ± 37	0.4
GCN5	H3K14	354 ± 2.1	995 ± 198	356
	H3hK14	0.87 ± 0.1	878.2 ± 157	1.0
	H3h_2_K14	0.40 ± 0.1	1,051 ± 226	0.4
	H3K9	3.81 ± 0.5	1910 ± 202	2.0
	H3hK9	0.76 ± 0.2	1624 ± 157	0.5

Examination of the secondary site of acetylation (H3K9) revealed a different trend, where H3hK9 and H3h_2_K9 were found to be more comparable but still poorer substrates to H3K9, with a 15- and 30-fold drop in k_cat_/K_m_values for PCAF and fourfold drop for GCN5-catalyzed acetylation of H3hK9. A significant decrease in catalytic efficiency may be the result of substrates clashing in the active site, driven by the loss of a catalytically required water molecule^55^. Previous work showed that GCN5 preferentially acetylates K14 over K9 in wild-type histone 3 with a ([k_cat_/K_m_]^K14^/[k_cat_/K_m_]^K9^) value of 57^50^. In our study the analysis of H3 substrate selectivity yielded ([k_cat_/K_m_]^K14^/[k_cat_/K_m_]^K9^) values 43 for PCAF and 177 for GCN5.

**Figure 5:**
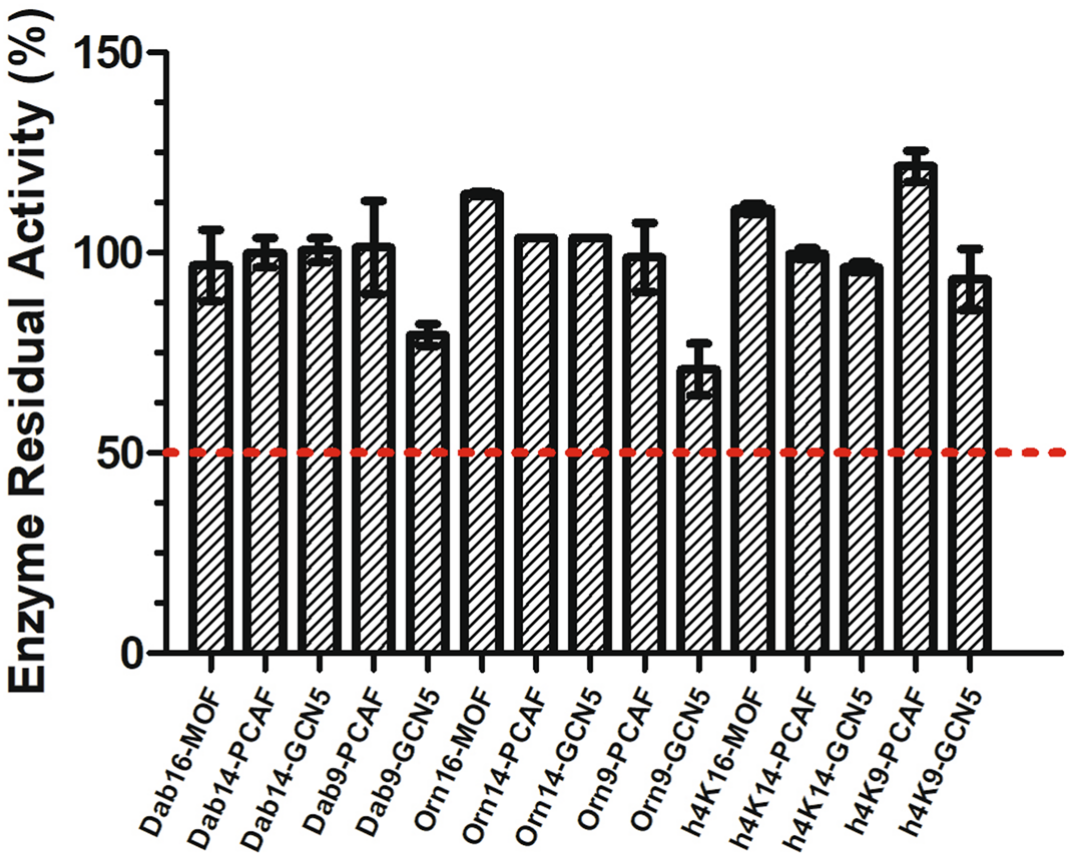
Enzymes’ residual activity upon incubation of MOF (0.2 µM), PCAF (0.5 µM) or GCN5 (0.5 µM) with H4K*16 (MOF) or H3K*14/H3K*9 (PCAF and GCN5) histone peptides.

Next, we proceeded by investigating the potential inhibitory activity of the histone peptides that did not act as KAT substrates. For practical reasons peptides bearing D-Lys and h_3_Lys analogs were not included in the evaluation, due to overlapping of MS signals with the respective natural peptide and acetylated product. Therefore, only histone peptides containing Dab, Orn and h_4_Lys were employed in this experiment. To our purpose, we carried out a single-point screening assay, by incubating an equimolar amount (100 µM) of the H4K16/H3K14/H3K9 peptides and the potential inhibitor peptides containing lysine analogs, and quenching the reactions after 1 h at 37 °C. MS analyses revealed that all tested peptides were not able to significantly inhibit KATs. In all experiments the presence of the unnatural histone peptides did not influence the catalytic properties of the enzymes (Supplementary Figs. 54–58), whose residual activity was found to be always greater than 50% when compared to the positive control consisting of the same reaction in the absence of an inhibitor peptide (Fig. 5). From these findings it can be concluded that IC_50_ values of histone peptide inhibitors are > 100 µM. A possible explanation of the discovered low inhibitory ability of histone peptides may lay in the low binding affinity (as reflected in high K_m_ values) showed in the enzyme kinetics experiments, which translates into the requirement of higher concentration of the peptides to appreciate competitive inhibition.

## Conclusion

Our combined synthetic and enzymatic investigations demonstrate that histone lysine acetyltransferases are special enzymes of the epigenetic machinery that have a capacity to acetylate histone peptides bearing lysine analogs with extended side chain. In line with their well-documented ability to catalyze the transfer of bulkier acyl moieties from their respective acyl CoA cosubstrates, human KATs showed an additional degree of substrate promiscuity by less efficiently accepting as substrates histone peptides bearing lysine analogs with longer side chains. Acetyltransferases MOF, PCAF and GCN5 were observed to catalyze the acetylation of lysine analogs bearing up to 7 carbons in the chain length (hK, h_2_K and h_3_K), both in primary and secondary sites of acetylation. Interestingly, none of the shorter (Dab, Orn) analogs, D-lysine or the longest lysine analog in our panel (h_4_K) were found as KAT substrates, indicating that although KATs alleged substrate promiscuity, the active site dynamics in KATs are still governed by stringent structural prerequisites. We further showed that the development of histone peptide based competitive inhibitors for KATs may suffer of lack of potency due to the relatively low binding affinities of these peptides towards KATs. Overall, this study shows that human KAT enzymes do have a broader substrate scope, as demonstrated by their capacity to catalyze acetylation of lysine analogs with longer side chain length. We believe that a better understanding of KAT catalysis, as demonstrated by basic molecular investigations on the substrate scope and biocatalytic potential reported here, provides an important knowledge for examination of posttranslational modifications of histone proteins and for rational drug design, targeting biomedically important KAT enzymes.

## Methods

### Synthesis and purification of histone peptides

All the histone peptides were manually synthesized employing standard Fmoc-SPPS chemistry on a 0.05 mmol scale on Wang resing (0.87 g/mmol loading capacity, 100–200 mesh). Upon standard amino acid (3.0 eq) activation with HOBt (3.6 eq) and DIC (3.3 eq) in DMF (final volume 2 mL, mixed for 2 min), the mixture was added to the Fmoc-deprotected growing peptide, and coupling reactions were carried out at room temperature for 2 h. Incorporation of newly synthesized amino acids (1.3 eq) were performed overnight at room temperature, with HOBt (1.56 eq) and DIC (1.43 eq). Fmoc deprotection was achieved by swelling the peptide in a solution of 20% piperidine in DMF (v/v), for 20 min. The qualitative Kaiser Test was employed to monitor the accomplishment of both coupling and deprotection steps. DMF washes (bubbling 3 × 2 min with N_2_) followed every deprotection and coupling steps. After the incorporation of the last amino acid, the peptides were Fmoc deproteced, dried over Et_2_O and cleaved off the resin employing a 95% TFA, 2.5% TIPS, 2.5% MQ cleavage cocktail for 4 h. After that the crude peptides were precipitated on cold Et_2_O (− 20 °C), lyophilised and purified by preparative HPLC on a Phenomenex Gemini-NX 3u C18 110A reversed-phase column (150 × 21.2 mm) with a flow rate of 10 mL/min at 30 °C. The eluent system constituted of a multistep gradient of Solvent B (0.1% TFA in CH_3_CN) in Solvent A (0.1% TFA in MQ water). Two different 30 min methods were used to purify the peptides: Method A) for H3 sequences: 0–3 min (3%), 3–15 min (3–15%), 15–20 min (15–30%), 20–22 min (30–100%), 22–25 (100%), 25–27 min (100–3%) and 27–30 min (3%) for re-equilibration of the column. Method B) for H4 sequences: 0–3 min (3%), 3–15 min (3–20%), 15–20 min (20–35%), 20–22 min (35–100%), 22–25 (100%), 25–27 min (100–3%) and 27–30 min (3%) for re-equilibration of the column. The pure fractions were combined and freeze-dried overnight to yield the target histone peptides as white-off fluffy solids. The purity of the synthesized peptides was then assessed via MALDI TOF–MS, ESI–MS and analytical traces were monitored at 215 nm on a Phenomenex Gemini 5 µm C18 110 Å LC column at a flow rate of 1 mL/min.

### Expression and purification of KATs

#### MOF

Plasmid carrying recombinant His-tagged Human MOF catalytic domain (residues 125–458 in pET19b) was gently provided by Professor Frank J. Dekker (University of Groningen). The protein was expressed and purified as previously described^23^. Briefly, *E. coli* BL21(DE3) cells enriched with human His-tagged MOF WT plasmid, were cultured in LB growth media supplemented with 50 µg/mL ampicillin at 37 °C, to an OD_600_ of 0.6, upon which expression was induced by addition of IPTG (0.3 mM final) and followed by incubation at 20 °C overnight. Harvested cells were pelleted and re-suspended into 10 mM Tris pH 7.4, 750 mM NaCl, 1% glycerol, 1 mM β-ME lysis buffer in presence of protease inhibitor cocktail (Roche) and lysate by sonication. The supernatant was incubated with Ni–NTA beads for 2 h at 4 °C. The beads were loaded on a gravity flow column and washed with 50 mM imidazole in lysis buffer. Subsequently, the protein was eluted with 10 mM MES, 750 mM NaCl, 10 mM Mg citrate, 250 mM imidazole, 1 mM β-ME, 1% glycerol, pH 6.5 and concentrated with a 30 kDa spinfilter device (AMICON, 30 MWCO). The protein was furtherly purified by size-exclusion chromatography (SEC) using the AKTA system, employing a Superdex 200 column equilibrated with 10 mM MES, 750 mM NaCl, 10 mM MgCitrate, 1 mM β-ME, 1% glycerol pH 6.5 at 0.5 mL/min flow speed and purity of the eluted protein was assessed with SDS-page. Pure fractions were pooled, rapidly flash-frozen and stored at -80 °C.

#### PCAF

Plasmid carrying recombinant SNAP-His-tagged Human PCAF catalytic domain (residues 498–658 in pET16b vector) was gently provided by Professor Milan Mrksich (Northwestern University). The protein was expressed as it follows: *E. coli* BL21(DE3) cells enriched with hPCAF plasmid, were cultured in 2xYT growth media supplemented with 100 µg/mL carbenicillin at 37 °C, to an OD_600_ of 0.6, upon which expression was induced by addition of IPTG (0.5 mM final) and followed by incubation at 16 °C overnight. Harvested cells were pelleted and re-suspended into 50 mM Tris pH 8.5, 200 mM NaCl, 5 mM β-ME lysis buffer in presence of protease inhibitor cocktail (Roche) and lysate by sonication. The supernatant was incubated with Ni–NTA beads for 2 h at 4 °C. The beads were loaded on a gravity flow column and washed with 50 mM Tris pH 8.5, 200 mM NaCl, 50 mM imidazole, 5 mM β-ME. Subsequently, the protein was eluted with 50 mM Tris pH 8.5, 200 mM NaCl, 300 mM imidazole, 5 mM β-ME and concentrated using a 30 kDa spinfilter device (AMICON, 30 MWCO). The protein was furtherly purified by size-exclusion chromatography (SEC) using the AKTA system, employing a Superdex 75 column equilibrated with 50 mM Tris pH 8.0, 200 mM NaCl, 1 mM DTT at 0.5 mL/min flow speed. The purity of the eluted protein was assessed with SDS-page. Pure fractions were pooled, rapidly flash-frozen and stored at -80 °C.

#### GCN5

Plasmid carrying recombinant His-tagged Human GCN5 catalytic domain (residues 497–662 in pET28a-LIC vector) was purchased from Addgene (25,482). The protein was expressed and purified as previously described^56^. Briefly, *E. coli* BL21(DE3) cells enriched with hGCN5 WT plasmid were cultured in TB growth media supplemented with 50 µg/mL kanamycin at 37 °C to an OD_600_ of 0.6, upon which expression was induced by addition of IPTG (0.5 mM final) and followed by incubation at 16 °C overnight. Harvested cells were pelleted and re-suspended into 50 mM Na_2_HPO_4_ pH 7.5, 500 mM NaCl, 5% glycerol, 1 mM β-ME lysis buffer in presence of protease inhibitor cocktail (Roche, Basel, Switserland) and lysate by sonication. The supernatant was incubated with Ni–NTA beads for 2 h at 4 °C. The beads were loaded on a gravity flow column and washed with 20 mM HEPES–NaOH pH 7.5, 500 mM NaCl, 50 mM imidazole, 5% glycerol, 1 mM β-ME. Subsequently, the protein was eluted with 20 mM Hepes–NaOH pH 7.5, 500 mM NaCl, 250 mM imidazole, 5% glycerol, 1 mM β-ME and the buffer was exchanged to 20 mM Hepes–NaOH pH 7.5, 150 mM NaCl, 1 mM βME by concentration with a 10 kDa spinfilter device (AMICON, 10 MWCO). Purity of the eluted protein was assessed with SDS-page, and GCN5 was separated in aliquots, rapidly flash-frozen and stored at -80 °C.

### MALDI-TOF MS enzymatic assays

The KAT enzymatic activity towards histone peptides was measured at different time points under standard conditions (2 µM KAT enzyme, 100 µM peptide, 300 µM AcCoA) in the reaction buffer (50 mM HEPES, 0.1 mM EDTA, 1 mM DTT, pH = 8.0). The reactions were carried out in a final volume of 50 µL and shaken up in a Thermomixer C at 750 rpm, at 37 °C. The reactions were quenched by the addition of TFA 10% in MilliQ water at different time points. All reactions were aliquoted and mixed 1:2 with a solution of α-Cyano-4-hydroxycinnamic acid (CHCCA) in 1:1 MQ and ACN (0.1% TFA) and loaded onto an MTP 384 polished steel target to be analyzed by a UltrafleXtreme-II tandem mass spectrometer (Bruker, Billerica, MA, USA).

### MALDI-TOF MS kinetics assays

Histone peptides kinetics evaluation was carried out with a MALDI-TOF MS assay under steady-state conditions^57,58^. Histone peptides (1,050–0 µM) were incubated with AcCoA (100 µM) and the reactions were started by the addition of the enzyme (MOF 570 nM, PCAF and GCN5 50 nM) in a final volume of 20 µL in kinetic buffer (50 mM HEPES, 0.1 mM EDTA, 0.01% TRITON-X, pH = 7.4). For histone peptides bearing unnatural lysine analogs, higher concentration of enzymes was used (MOF, PCAF and GCN5 2 µM). Steady state conditions were guaranteed by employing saturating concentrations of AcCoA (> 5 × K_m_ value). AcCoA stock solutions in milli-Q water were calibrated with a NanoDrop 2000 spectrophotometer (Thermo Scientific, Waltham, MA, USA), employing the characteristic molar extinction coefficient ε_260nm_ = 16,400 M^−1^ cm^−1^. Reactions were incubated at 37 °C, shaken at 750 rpm and quenched with TFA 10% in milli-Q water at different time points, within linear production of acetylated peptides extrapolated from time-course plots. All reactions were aliquoted and mixed 1:2 with a solution of α-cyano-4-hydroxycinnamic acid (CHCCA) in 1:1 MQ and ACN (0.1% TFA) and loaded onto MTP 384 polished steel target to be analyzed by UltrafleXtreme-II tandem mass spectrometer (Bruker). The amount of acetylated peptide was calculated by integration of the product peak area and divided it by the amount of unacetylated peptide, taking in account all the ionic species, at any concentration points using the FlexAnalysis software. Kinetic values were extrapolated by fitting V_0_ values (µM of produced peptide per minutes) and histone peptide concentrations to the Michaelis–Menten equation using the GraphPad Prism 5 software. Kinetic experiments were carried out in duplicates and final values are reported as value ± SD.

### Inhibition assays

Equimolar amount of the natural histone peptide and the histone peptide possessing the unnatural lysine analog (1:1; 100 µM) were incubated in the presence of AcCoA (300 µM) in the reaction buffer (50 mM HEPES, 0.1 mM EDTA, 1 mM DTT, pH = 8.0). The reactions were started by the addition the enzymes MOF (200 nM), PCAF (500 nM) and GCN5 (500 nM), and carried out at 37 °C for 1 h, after which they were quenched by the addition of 10% TFA in MQ and analyzed by MALDI-TOF MS. Every experiment was carried out in triplicates and normalized to the positive control reaction. Data are shown as the mean value ± SEM.

## Supplementary information

Supplementary file1 (PDF 13910 kb)
