# Supplementary material for: Effect of lysine side chain length on histone lysine acetyltransferase catalysis
Source: Sci Rep. 2020 Aug 3;10:13046. doi: 10.1038/s41598-020-69510-0 (PMC7400623; doi:10.1038/s41598-020-69510-0)
Supplement: Supplementary file 1 — Supplementary file1 (PDF 13910 kb) [file 41598_2020_69510_MOESM1_ESM.pdf]

Supplementary Information

Effect of Lysine Side Chain Length on Histone Lysine Acetyltransferase Catalysis

Giordano Proietti[1,2], Yali Wang [2,3], Giorgio Rainone[1] & Jasmin Mecinović[1,2*]

[1] Department of Physics, Chemistry and Pharmacy, University of Southern Denmark, Campusvej 55, 5230 Odense, Denmark.

[2] Institute for Molecules and Materials, Radboud University, Heyendaalseweg 135, 6525 AJ Nijmegen, The Netherlands.

[3] Department of Blood Transfusion, China-Japan Union Hospital, Jilin University, 126 Xiantai Street, Changchun, 130033, P. R. China.

*e-mail: mecinovic@sdu.dk

Table of Contents

1	General experimental	3
2	Synthetic protocols	4
3	List of peptides	9
4	ESI-MS analysis of peptides	10
5	Analytical HPLC of peptides	11
6	Time course plot of KAT-catalyzed acetylation	23
7	MALDI-TOF MS supporting figures	24
8	Kinetics plots	57
9	NMR spectra	60
10	References	68

1. General Experimental

All commercially available reagents were purchased and used without further purification. Unless otherwise noted all the reagents were purchased from Sigma-Aldrich. Fmoc-L-hLys(Boc)-OH was purchased from Iris Biotech. Wang resin (100-200 mesh), Fmoc-Orn(Boc)-OH, Fmoc-Dab(Boc)-OH, Fmoc-D-Lys(Boc)-OH were purchased from Bachem AG. Di-tert-butyliminodicarboxylate was purchased from Fluorochem. Reactions were magnetically stirred and monitoring by thin layer chromatography (TLC) was performed on glass backed silica sheets (Merck Silica Gel 60 F254) and the plates were visualized by UV fluorescence (254 nm) and/or spraying with potassium permanganate (KMnO4) or ninhydrin. Flash column chromatography was performed on a Biotage Isolera ONE system employing Silicycle SiliaSEP prepacked columns with the designated solvent systems. ^{1}H NMR and ^{13}C NMR spectra were obtained using a Bruker Avance III 400 MHz. ^{1}H NMR chemical shift values are reported as δ in units of parts per million (ppm) relative to the internal standard tetramethylsilane (TMS, δ = 0 ppm). ^{13}C NMR shifts are reported as δ in units of parts per million (ppm) and the spectra were internally referenced to the residual solvent signal (CHCl3 δ = 77.0 ppm). Coupling constant are reported as *J* values in Hertz (HZ). The following abbreviations were used to explain multiplicities: s = singlet, d = doublet, t = triplet, q = quartet, m = multiplet, br = broad. High-resolution mass spectra were obtained with a JEOL AccuTOF CS JMS-T100CS mass spectrometer. LC-MS analysis for the final side chain extended lysine analogs and peptides was carried out on a Thermo Finnigan LCQ-Fleet ESI-ion trap (Thermofischer, Breda, the Netherlands) bearing a Phenomenex Gemini-NX C18 3 µM 50 x 2.0 mm (Phenomenex, Utrecht, The Netherlands). Lyophilization of peptides was performed with an ilShin Freeze Dryer (ilShin, Ede, The Netherlands).

2. Synthetic protocols

General procedures:

General procedure A: preparation of diBoc-protected amino alkenes **1-3**

To a stirring suspension of di-tert-butyliminodicarboxylate (1.30 g, 6 mmol, 1.0 eq), lithium iodide (0.04 g, 0.3 mmol. 0.05 eq) and cesium carbonate (3.91 g, 12 mmol, 2.0 eq) in 2-butanone (30 mL), bromoalkene (9 mmol, 1.5 eq) were added dropwise and the reaction was left stirring under reflux for 16 hours. Subsequently, the reaction mixture was brought to room temperature, quenched with brine (60 mL) and extracted with Et_2O (3 x 30 mL). The organic layers were combined, dried over $NaSO_4$, and concentrated *in vacuo*. The crude mixture was purified by flash chromatography.

General procedure B: preparation of unsaturated Fmoc-Lys$(Boc)_2$-OH analogs **4-6**

Fmoc-Allyl glycine (0.1 g, 0.3 mmol, 1.0 eq) and its cross-metathesis partner diBoc-protected amino alkenes **1-3** (0.9 mmol, 3.0 eq) were dissolved in dry CH_2Cl_2 (30 mL) in a flamed dry flask under inert atmosphere. Subsequently, Hoveyda-Grubbs II generation catalyst (10% mol, 18.7 mg) was added and the dark green solution was left stirring under reflux for 16 h. The reaction mixture was then concentrated *in vacuo*. The crude mixture was purified by flash chromatography to afford a mixture of cis and trans isomers. Only partial characterization (^{1}H NMR, HRMS) was possible for this analogs.

General procedure B: preparation of Fmoc-Lys$(Boc)_2$-OH analogs **7-9**

A solution of unsaturated analogs **4-6** (0.12 mmol, 1 eq) in EtOH (5 mL) was added to a stirring suspension of Pd/C (10 wt. %) in EtOH (15 mL) being kept under N_2 flow. The N_2 stream was swiftly switched to H_2 (1 atm) and the reaction was left stirring at RT. The progress of the reaction was monitored by ESI-MS until full reduction of the starting material. Subsequently, the reaction mixture was filtered through Celite and concentrated *in vacuo* to yield the final compounds without further purification.

Di-*tert*-butyl N-4-pentenyliminodicarboxylate (1).

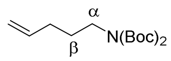

Synthesized via the general procedure A starting from 5-bromopent-1-ene (1.06 mL, 9 mmol). Purified by flash chromatography (0-10% EtOAc in *n*-heptane, 8 cv) to yield **1** as a colourless oil (1.59 g, 93%). to yield **3** as a Colourless oil (1.59 g, 93%). R_f = 0.65 (EtOAc/*n*-heptane 1:3). ^{1}H NMR ($CDCl_3$, 400 MHz): δ 5.81 (ddt, *J* = 16.9, 10.2, 6.6 Hz, 1H, CH_2=C*H*), 5.09 – 4.92 (m, 2H, CH_2=CH), 3.57 (t, *J*=7.5 Hz 2H, α-CH_2), 2.06 (q, *J* = 7.2 Hz, 2H, CH-CH_2), 1.67 (p, *J* = 7.5 Hz, 2H, β-CH_2), 1.51 (s, 18H, 6 x CH_3 Boc). ^{13}C NMR (101 MHz, $CDCl_3$) δ 152.67, 137.89, 114.90, 82.09, 46.05, 31.01, 28.10. ESI-MS [M+Na]$^+$: 308.0 m/z. Spectroscopic data in line with previous characterization.[1]

D**i-*tert*-butyl N-4-hexenyliminodicarboxylate (2).**

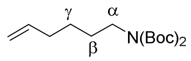

Synthesized via the general procedure A starting from 6-bromohex-1-ene (1.2 mL, 9 mmol). Purified by flash chromatography (0-5% EtOAc in *n*-heptane) to yield **2** as a colourless oil (1.26 g, 70%). R_f = 0.74 (EtOAc/*n*-heptane 1:3). ^{1}H NMR ($CDCl_3$, 400 MHz): δ 5.80 (ddt, *J* = 16.9, 10.2, 6.7 Hz, 1H, CH_2=C*H*), 5.04 – 4.93 (m, 2H), 4.95 (ddt, *J* = 10.2, 2.2, 1.2 Hz, 1H, CH_2=CH), 3.55 (t, *J*=7.5 Hz, 2H, α-CH_2), 2.05 (q, *J* = 7.1 Hz, 2H, CH-CH_2), 1.58 (p, 2H, *J* = 7.5 Hz β-CH_2), 1.51 (s, 18H, 6 x CH_3 Boc), 1.45 – 1.33 (m, 2H, γ-CH_2). ^{13}C NMR (101 MHz, $CDCl_3$) δ 152.67, 137.89, 114.90, 82.09, 46.05, 31.01, 28.13, 28.10. ESI-MS [M+Na]$^+$: 321.9 m/z. Spectroscopic data in line with previous characterization.[2]

Di-*tert*-butyl N-4-heptenyliminodicarboxylate (3).

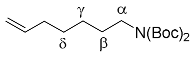

Synthesized via the general procedure A starting from 7-bromohept-1-ene (1.37 mL, 9 mmol). Purified by flash chromatography (0-5% EtOAc in *n*-heptane, 10 cv) to yield **3** as a colourless oil (1.58 g, 84%). R_f = 0.67 (EtOAc/*n*-heptane 1:3). ^{1}H NMR ($CDCl_3$, 400 MHz): δ 5.79 (ddt, *J* = 16.9, 10.2, 6.6 Hz, 1H, CH_2=C*H*), 5.02-4.91 (m, 2H) 4.93 (ddt, *J* = 10.2, 2.3, 1.2 Hz, 1H), 3.55 (t, *J* = 7.5 2H, α-CH_2), 2.06 (q, *J* = 7.2 Hz, 2H, CH-CH_2), 1.56 (p, 2H, *J* = 7.5 Hz β-CH_2), 1.51 (s, 18H, 6 x CH_3 Boc), 1.45 – 1.36 (m, 2H, γ-CH_2), 1.35 – 1.25 (m, 2H, δ-CH_2). ^{13}C NMR (101 MHz, $CDCl_3$) δ 152.72, 138.83, 114.38, 81.98, 46.42, 33.70, 28.90, 28.58, 28.10, 26.29. ESI-MS [M+Na]$^+$: 335.9 m/z.

(S,E)-2-((((9H-fluoren-9-yl)methoxy)carbonyl)amino)-8-(bis(tert-butoxycarbonyl)amino)oct-4-enoic acid (4).

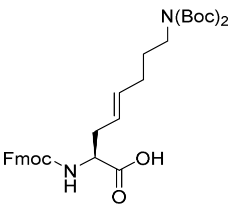

Synthesized via the general procedure B employing **1** (0.25 g, 0.9 mmol) as the cross-metathesis partner. Purified by flash chromatography (20-80% EtOAc/ cyclohexane +0.1% AcOH, 8 cv) to afford **4** as a colourless oil mixture of cis and trans isomers (71 mg 40%). R_f = 0.33 (EtOAc + 0.1% AcOH). (^{1}H NMR (400 MHz, Methanol-d_4) δ 7.78 (d, *J* = 7.5 Hz, 2H, Fmoc-*H*), 7.65 (dd, *J* = 7.5, 4.2 Hz, 2H, Fmoc-*H*), 7.38 (t, *J* = 7.4 Hz, 2H, Fmoc-*H*), 7.29 (t, *J* = 7.2 Hz, 2H, Fmoc-*H*), 5.64 – 5.37 (m, 2H, C*H*=C*H*), 4.36 – 4.28 (m, 2H), 4.25 – 4.16 (m, 2H), 3.53 (t, *J* = 7.4 Hz, 2H, CH_2-N(Boc)$_2$), 2.65 – 2.36 (m, 2H, β-CH_2), 2.00 (q, *J* = 7.1 Hz, 2H, ε–CH_2), 1.61 (p, *J* = 7.4 2H, ζ–CH_2), 1.46 (s, 18H, 6 x CH_3 Boc). HRMS [M+Na]$^+$ calculated 617.2834, found 617.2825.

(S,E)-2-((((9H-fluoren-9-yl)methoxy)carbonyl)amino)-9-(bis(tert-butoxycarbonyl)amino)non-4-enoic acid (5).

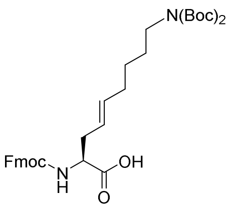

Synthesized via the general procedure B employing **2** (0.27 g, 0.9 mmol) as the cross-metathesis partner. Purified by flash chromatography (40-60% EtOAc + 1% AcOH in cyclohexane, 8 cv) to afford **5** as a brown oil mixture of cis and trans isomers (95 mg, 50%). R*f* = 0.33 (EtOAc + 0.1% AcOH). ^{1}H NMR (400 MHz, Methanol-d_4) δ 7.79 (d, *J* = 7.5 Hz, 2H, Fmoc-*H*), 7.67 (t, *J* = 7.4 Hz, 2H, Fmoc-*H*), 7.38 (t, *J* = 7.4 Hz, 2H, Fmoc-*H*), 7.30 (t, *J* = 7.5 Hz, 2H), 5.64 – 5.37 (m, 2H, C*H*=C*H*), 4.36 – 4.28 (m, 2H), 4.24 – 4.17 (m, 2H), 3.58 – 3.47 (m, 2H), 2.65-2.36 (m, 2H), 2.00 (q, *J* = 7.1 Hz 2H, ε–CH_2), 1.60 – 1.51 (m, 2H), 1.46 (s, 18H, 6 x CH_3 Boc), 1.40 – 1.26 (m, 2H). HRMS [M+Na]$^+$calculated 631.2995, found 631.2983.

(S,E)-2-((((9H-fluoren-9-yl)methoxy)carbonyl)amino)-10-(bis(tert-butoxycarbonyl)amino)dec-4-enoic acid (6).

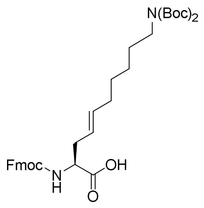

Synthesized via the general procedure B employing **3** (0.28 g, 0.9 mmol) as the cross-metathesis partner. purified by flash chromatography (40-60% EtOAc + 1% AcOH in cyclohexane, 2 cv) to afford **6** as a brown oil (76 mg, 41%). R_f = 0.33 (in EtOAc + 0.1% AcOH). δ 7.78 (d, *J* = 7.5 Hz, 2H), 7.65 (dd, *J* = 7.5, 4.2 Hz, 2H), 7.38 (t, *J* = 7.4 Hz, 2H), 7.29 (t, *J* = 7.2 Hz, 2H), 5.74 – 5.32 (m, 2H), 4.36-4.28 (m, 2H), 4.25 – 4.16 (m, 2H), 3.55 – 3.49 (m, 2H), 2.66-2.32 (m, 2H), 2.11 – 2.96 (m, 2H), 1.63 – 1.51 (m, 2H), 1.46 (s, 18H), 1.44 – 1.22 (m, 4H). HRMS $[M+Na]^+$ calculated 645.3151, found 645.3135

(S)-2-((((9H-fluoren-9-yl)methoxy)carbonyl)amino)-8-(bis(tert-utoxycarbonyl)amino)octanoic acid (7).

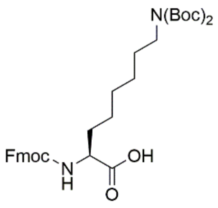

Synthesized via the general procedure C for reduction of **4** (71 mg, 0.12 mmol). Off-white solid (55 mg, 76%). R_f = 0.33 (EtOAc + 0.1% AcOH). (^{1}H NMR (400 MHz, Methanol-d_4) δ 7.76 (d, *J* = 7.6 Hz, 2H, Fmoc-*H*), 7.65 (t, *J* = 7.4 Hz, 2H, Fmoc-*H*), 7.36 (t, *J* = 7.5 Hz, 2H, Fmoc-*H*), 7.27 (t, *J* = 7.5 Hz, 2H, Fmoc-*H*), 4.32 (d, *J* = 7.0 Hz, 2H, Fmoc-CH_2), 4.25 (t, *J* = 7.1 Hz, 1H, Fmoc-C*H*), 4.11 (dd, *J* = 9.3, 4.6 Hz, 1H, α-*H*), 3.53 (t, *J* = 7.4 Hz, 2H, CH_2-N(Boc)$_2$), 1.95 – 1.80 (m, 1H, β-CH_2), 1.75 – 1.65 (m, 1H, β-CH_2), 1.54 (p, *J* = 7.2 Hz, 2H, ζ–CH_2), 1.51 (s, 18H, 6 x CH_3 Boc), 1.46 – 1.28 (m, 6H, γ, δ, ε–CH_2). ^{13}C NMR (101 MHz, MeOD) δ 176.03, 158.70, 154.16, 145.37, 145.19, 142.60, 128.78, 128.15, 126.28, 120.91, 83.58, 67.93, 55.23, 48.44, 47.38, 32.59, 30.01, 29.83, 28.31, 27.60, 26.87. HRMS $[M+Na]^+$ calculated 619.2995, found 619.2976

(S)-2-((((9H-fluoren-9-yl)methoxy)carbonyl)amino)-9-(bis(tertbutoxycarbonyl)amino)nonanoic acid (8).

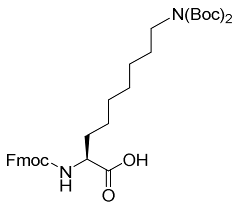

Synthesized via the general procedure C for reduction of **5** (95 mg, 0.15 mmol, 1 eq). Dark brown oil (79 mg, 88%). R_f = 0.35 (in EtOAc + 0.1% AcOH). ^{1}H NMR (400 MHz, Methanol-d_4) δ 7.80 (d, *J* = 7.6 Hz, 2H), 7.68 (t, *J* = 8.1 Hz, 2H, Fmoc-$\underline{H}$), 7.39 (t, *J* = 7.5 Hz, 2H, Fmoc-$\underline{H}$), 7.31 (t, *J* = 7.5 Hz, 2H, Fmoc-$\underline{H}$), 4.01 – 4.32 (m, 2H, Fmoc-$C\underline{H}_2$), 4.23 (t, *J* = 7.1 Hz, 1H, Fmoc-$C\underline{H}$), 4.16 (dd, *J* = 9.2, 4.7 Hz, 1H, α-$\underline{H}$), 3.55 (t, *J* = 7.4 Hz, 2H, $C\underline{H}_2$-N(Boc)$_2$), 1.90 – 1.81 (m, 1H, β-$C\underline{H}_2$), 1.75 – 1.64 (m, 1H, β-$C\underline{H}_2$) 1.57 (p, *J* = 7.3 Hz, 2H, η-$C\underline{H}_2$), 1.51 (s, 18H, 6 x $C\underline{H}_3$ Boc), 1.46 – 1.26 (m, 8H, γ, δ, ε, ζ-$C\underline{H}_2$). ^{13}C NMR (101 MHz, MeOD) δ 176.23, 158.62, 154.11, 145.32, 145.15, 142.55, 128.75, 128.14, 128.12, 126.26, 120.89, 83.53, 67.91, 55.36, 48.40, 47.37, 32.71, 30.16, 30.09, 30.01, 28.31, 27.64, 26.80. HRMS $[M+Na]^+$ calculated 633.3151, found 633.3134

(S)-2-((((9H-fluoren-9-yl)methoxy)carbonyl)amino)-10-(bis(tert-butoxycarbonyl)amino)decanoic acid (9).

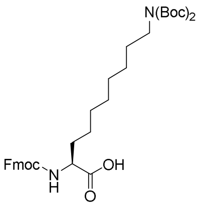

Synthesized via the general procedure C for reduction of of **6** (76 mg, 0.12 mmol). White solid (60 mg, 80%). R_f = 0.33 (in EtOAc + 0.1% AcOH). (^{1}H NMR (400 MHz, Methanol-d_4) δ 7.79 (d, *J* = 7.5 Hz, 2H), 7.67 (t, *J* = 7.1 Hz, 2H), 7.38 (t, *J* = 7.3 Hz, 2H), 7.30 (t, *J* = 7.4 Hz, 2H), 4.46 – 4.30 (m, 2H, Fmoc-$C\underline{H}_2$), 4.22 (t, *J* = 7.0 Hz, 1H,), 4.13 (dd, *J* = 9.2, 4.8 Hz, 1H, α-$\underline{H}$), 3.54 (t, *J* = 7.3 Hz, 2H, $C\underline{H}_2$-N(Boc)$_2$), 1.89 – 1.76 (m, 1H, β-$C\underline{H}_2$), 1.73 – 1.62 (m, 1H, β-$C\underline{H}_2$), 1.59 – 1.52 (m, 2H, θ-$C\underline{H}_2$), 1.49 (s, 18H, 6 x $C\underline{H}_3$ Boc), 1.44 – 1.24 (m, 10H, γ, δ, ε, ζ, η-$C\underline{H}_2$). ^{13}C NMR (101 MHz, MeOD) ^{13}C NMR (101 MHz, MeOD) δ 176.27, 158.65, 154.14, 145.35, 145.18, 128.76, 128.15, 126.27, 120.91, 83.55, 67.92, 55.42, 48.43, 47.40, 32.76, 30.42, 30.20, 30.06, 30.02, 28.31, 27.69, 26.86. HRMS $[M+Na]^+$ calculated 647.3308, found 647.3289.

3. List of peptides

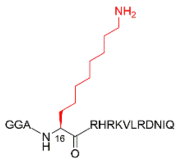

H4h$_4$K16

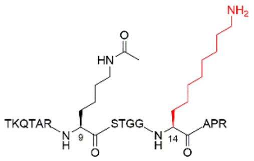

H3h$_4$K14

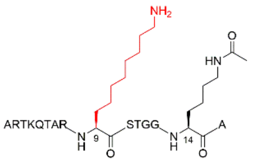

H3h$_4$K9

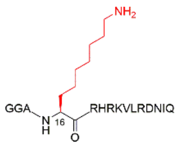

H4h$_3$K16

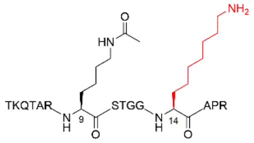

H3h$_3$K14

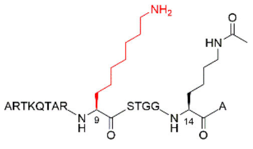

H3h$_3$K9

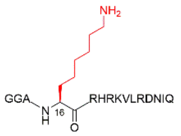

H4h$_2$K16

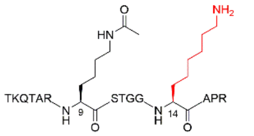

H3h$_2$K14

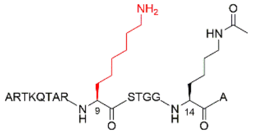

H3h$_2$K9

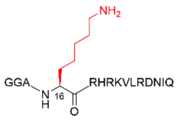

H4hK16

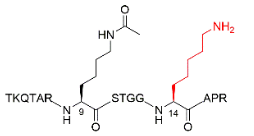

H3hK14

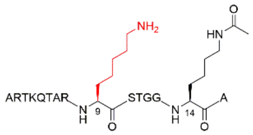

H3hK9

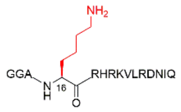

H4K16

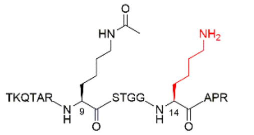

H3K14

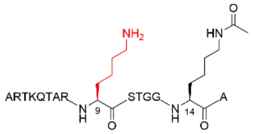

H3K9

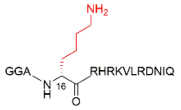

H4DK16

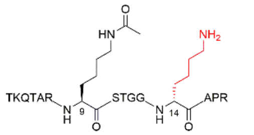

H3DK14

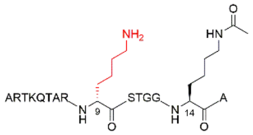

H3DK9

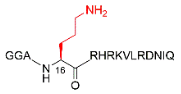

H4Orn16

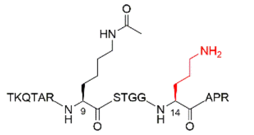

H3Orn14

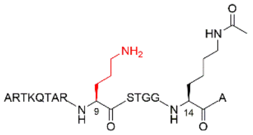

H3OrnK9

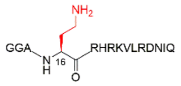

H4Dab16

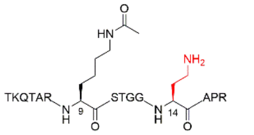

H3Dab14

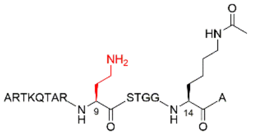

H3Dab9

4. Table S1. ESI-MS analysis of the peptides.

Entry	Peptide	Sequence	Formula	m/z calc.	$[M+2H]^{2+}$ found	$[M+3H]^{3+}$ found	$[M+4H]^{4+}$ found
1	H4Dab16	GGADab^{16}RHRKVLRDNIQ	$C_{71}H_{126}N_{30}O_{20}$	1718.9	860.5	574.0	430.8
2	H4Orn16	GGAOrn^{16}RHRKVLRDNIQ	$C_{72}H_{128}N_{30}O_{20}$	1732.9	867.5	578.9	434.3
3	H4DK16	GGAD-K^{16}RHRKVLRDNIQ	$C_{73}H_{130}N_{30}O_{20}$	1747.0	874.5	583.6	437.8
4	H4K16	GGAK^{16}RHRKVLRDNIQ	$C_{73}H_{130}N_{30}O_{20}$	1747.0	874.5	583.6	437.8
5	H4hK16	GGAhK^{16}RHRKVLRDNIQ	$C_{74}H_{132}N_{30}O_{20}$	1761.0	881.6	588.3	441.4
6	H4h$_2$K16	GGAh$_2$K^{16}RHRKVLRDNIQ	$C_{75}H_{134}N_{30}O_{20}$	1775.0	888.5	592.9	444.8
7	H4h$_3$K16	GGAh$_3$K^{16}RHRKVLRDNIQ	$C_{76}H_{136}N_{30}O_{20}$	1789.0	895.9	597.6	448.4
8	H4h$_4$K16	GGAh$_4$K^{16}RHRKVLRDNIQ	$C_{77}H_{138}N_{30}O_{20}$	1803.1	902.9	602.3	451.9
9	H3Dab14	TKQTARK^{Ac}STGGDab^{14}APR	$C_{65}H_{117}N_{25}O_{22}$	1599.9	801.5	534.3	400.9
10	H3Orn14	TKQTARK^{Ac}STGGOrn^{14}APR	$C_{66}H_{119}N_{25}O_{22}$	1613.9	807.9	538.9	404.5
11	H3DK14	TKQTARK^{Ac}STGGD-K^{14}APR	$C_{67}H_{121}N_{25}O_{22}$	1627.9	814.9	543.6	407.9
12	H3K14	TKQTARK^{Ac}STGGK^{14}APR	$C_{67}H_{121}N_{25}O_{22}$	1627.9	814.9	543.6	407.9
13	H3hK14	TKQTARK^{Ac}STGGhK^{14}APR	$C_{68}H_{123}N_{25}O_{22}$	1641.9	822.0	548.3	411.5
14	H3h$_2$K14	TKQTARK^{Ac}STGGh$_2$K ^{14}APR	$C_{69}H_{125}N_{25}O_{22}$	1656.9	829.0	553.0	415.0
15	H3h$_3$K14	TKQTARK^{Ac}STGGh$_3$K^{14}APR	$C_{70}H_{127}N_{25}O_{22}$	1669.9	836.0	557.6	418.5
16	H3h$_4$K14	TKQTARK^{Ac}STGGh$_4$K^{14}APR	$C_{71}H_{129}N_{25}O_{22}$	1683.9	843.0	562.3	422.0
17	H3Dab9	ARTKQTARDab^{9}STGGK^{Ac}A	$C_{63}H_{115}N_{25}O_{22}$	1573.9	787.9	525.6	394.4
18	H3Orn9	ARTKQTAROrn^{9}STGGK^{Ac}A	$C_{64}H_{117}N_{25}O_{22}$	1587.9	794.2	530.3	397.9
19	H3D-K9	ARTKQTARD-K^{9}STGGK^{Ac}A	$C_{65}H_{119}N_{25}O_{22}$	1601.9	801.9	534.9	401.5
20	H3K9	ARTKQTARK^{9}STGGK^{Ac}A	$C_{65}H_{119}N_{25}O_{22}$	1601.9	801.9	534.9	401.5
21	H3hK9	ARTKQTARhK^{9}STGGK^{Ac}A	$C_{66}H_{121}N_{25}O_{22}$	1615.9	808.9	539.8	404.9
22	H3h$_2$K9	ARTKQTARh$_2$K^{9}STGGK^{Ac}A	$C_{67}H_{123}N_{25}O_{22}$	1629.9	815.9	544.4	408.4
23	H3h$_3$K9	ARTKQTARh$_3$K^{9}STGGK^{Ac}A	$C_{68}H_{125}N_{25}O_{22}$	1643.9	822.8	549.1	411.9
24	H3h$_4$K9	ARTKQTARh$_4$K^{9}STGGK^{Ac}A	$C_{69}H_{127}N_{25}O_{22}$	1657.9	829.9	553.8	415.4

5. Analytical HPLC of peptides

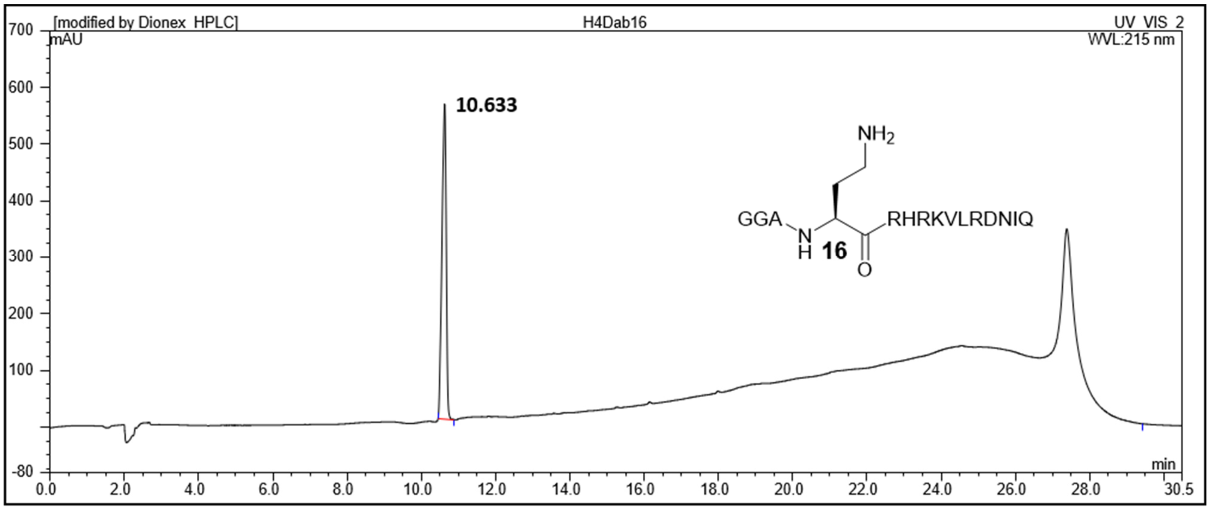

Figure S1. Analytical HPLC of the H4Dab16 peptide after RP-HPLC purification.

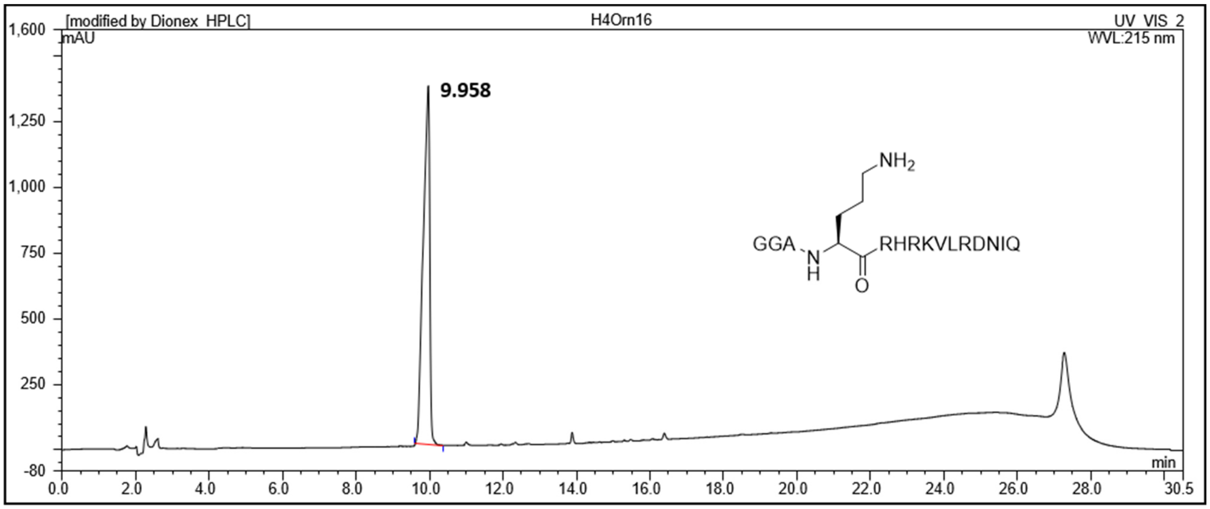

Figure S2. Analytical HPLC of the H4Orn16 peptide after RP-HPLC purification.

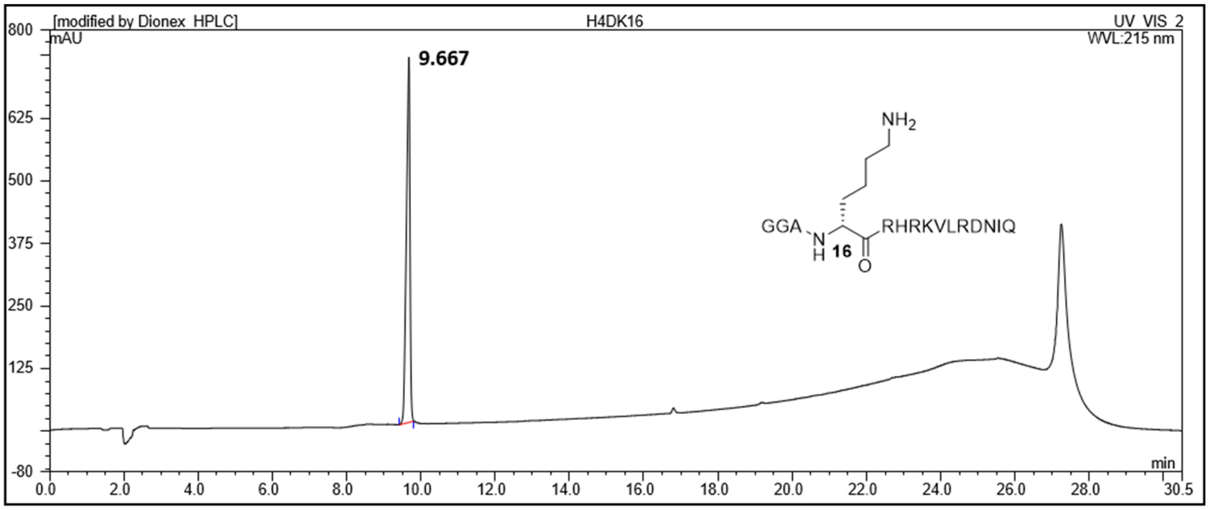

Figure S3. Analytical HPLC of the D-H4K16 peptide after RP-HPLC purification.

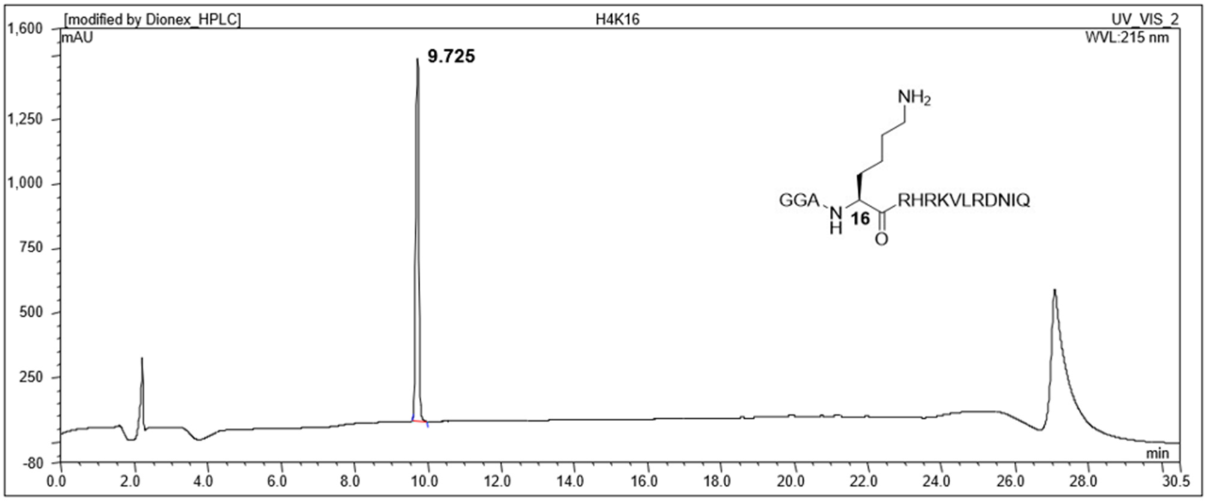

Figure S4. Analytical HPLC of the H4K16 peptide after RP-HPLC purification.

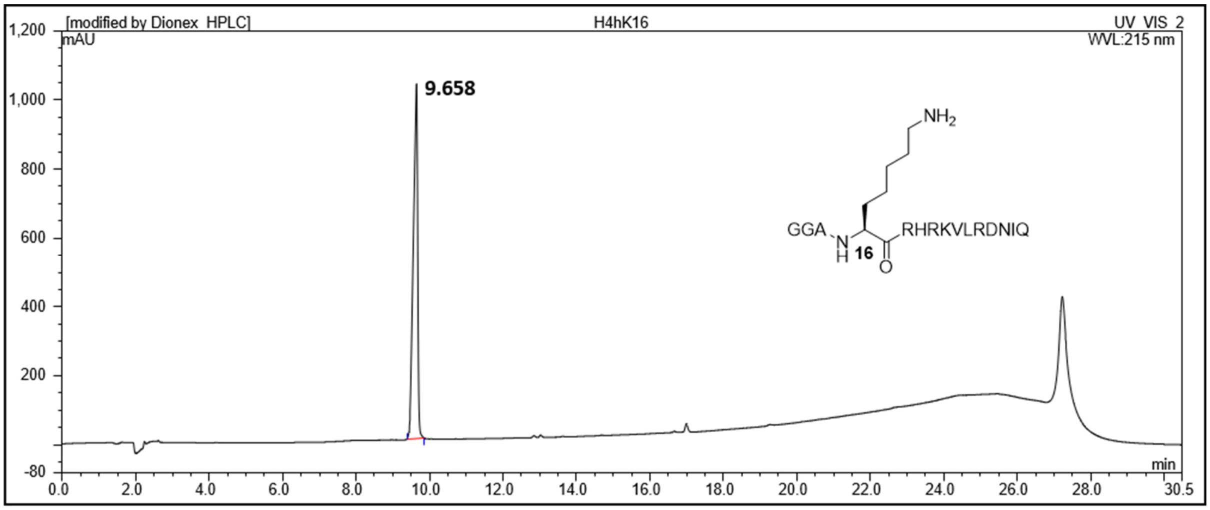

Figure S5. Analytical HPLC of the H4hK16 peptide after RP-HPLC purification.

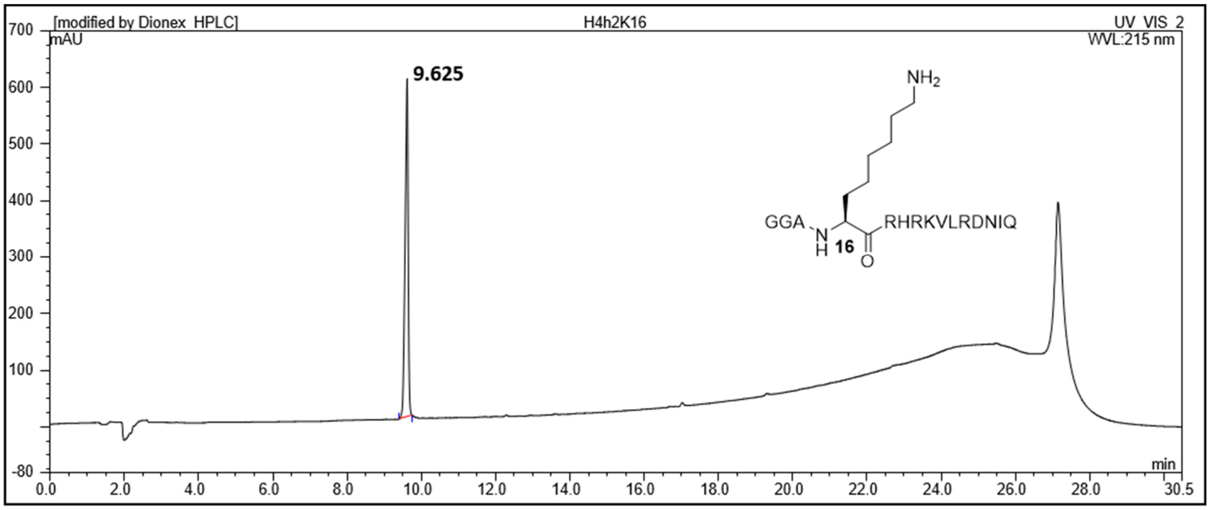

Figure S6. Analytical HPLC of the $H4h_2K16$ peptide after RP-HPLC purification.

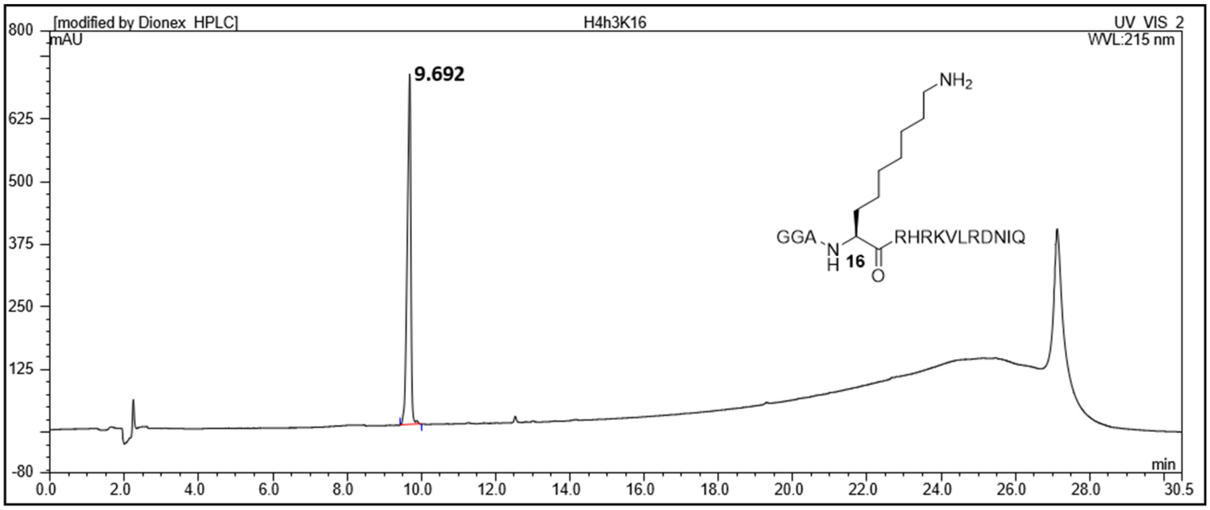

Figure S7. Analytical HPLC of the H4h$_3$K16 peptide after RP-HPLC purification.

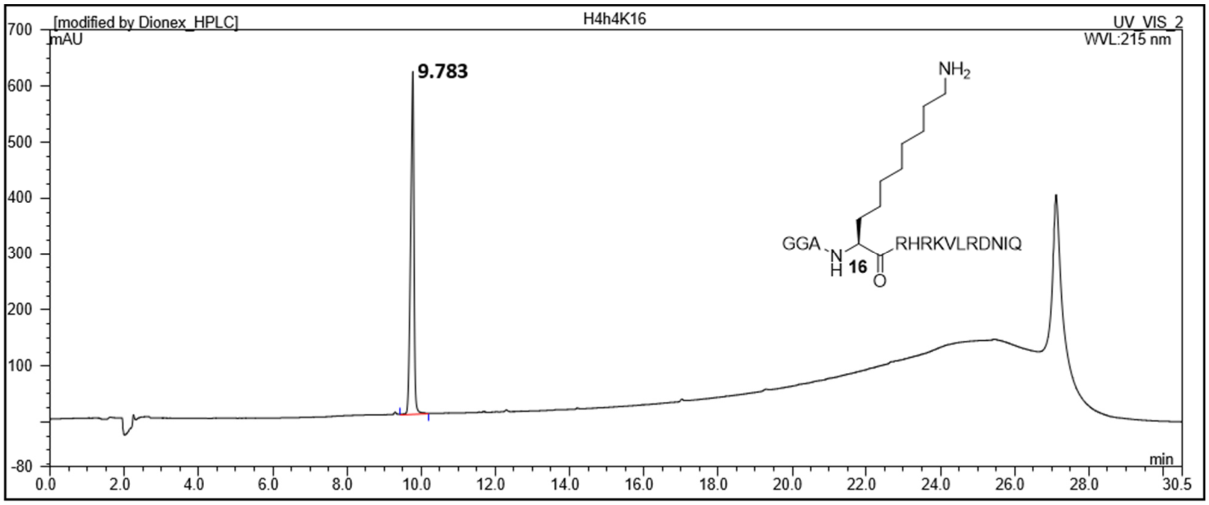

Figure S8. Analytical HPLC of the H4h$_4$K16 peptide after RP-HPLC purification.

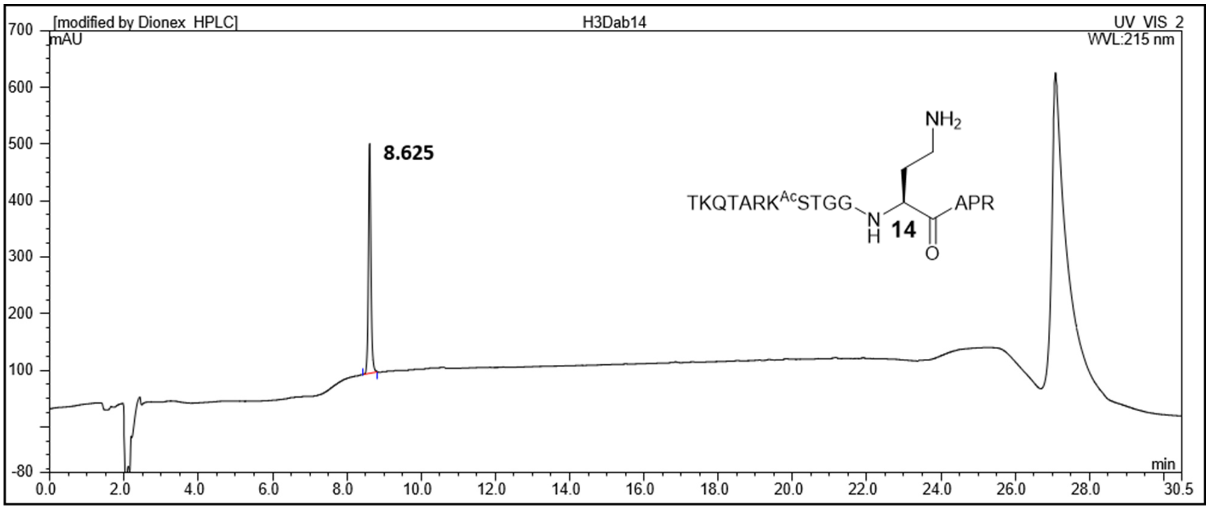

Figure S9. Analytical HPLC of the H3Dab14 peptide after RP-HPLC purification.

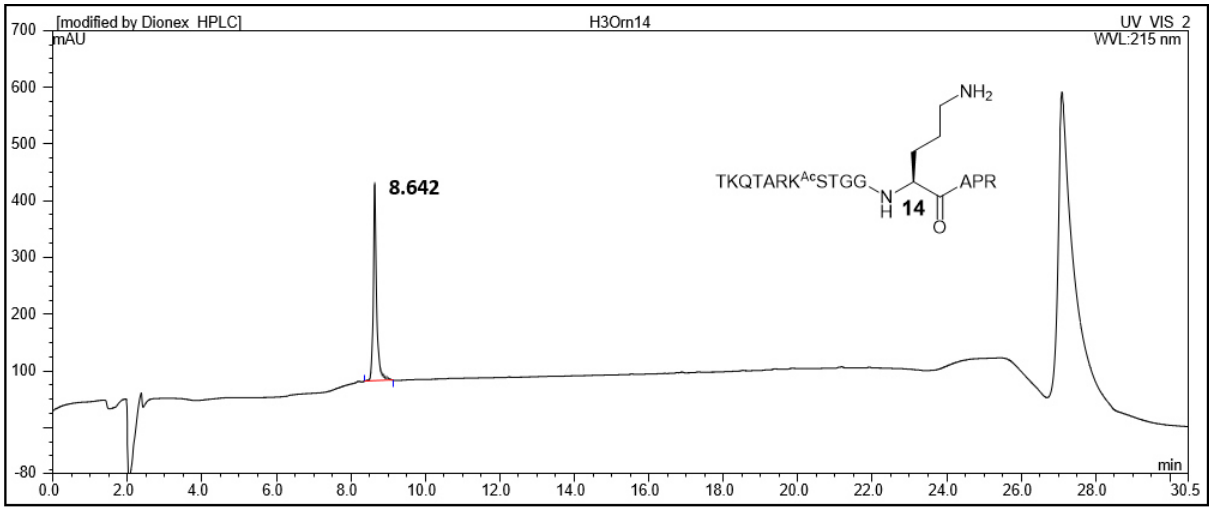

Figure S10. Analytical HPLC of the H3Orn14 peptide after RP-HPLC purification.

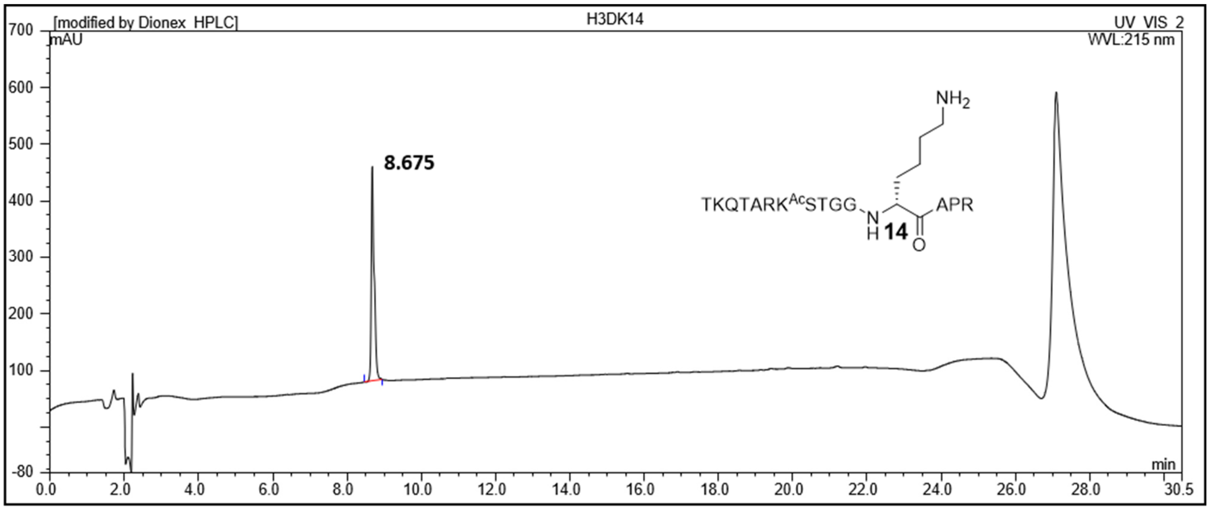

Figure S11. Analytical HPLC of the D-H3K14 peptide after RP-HPLC purification.

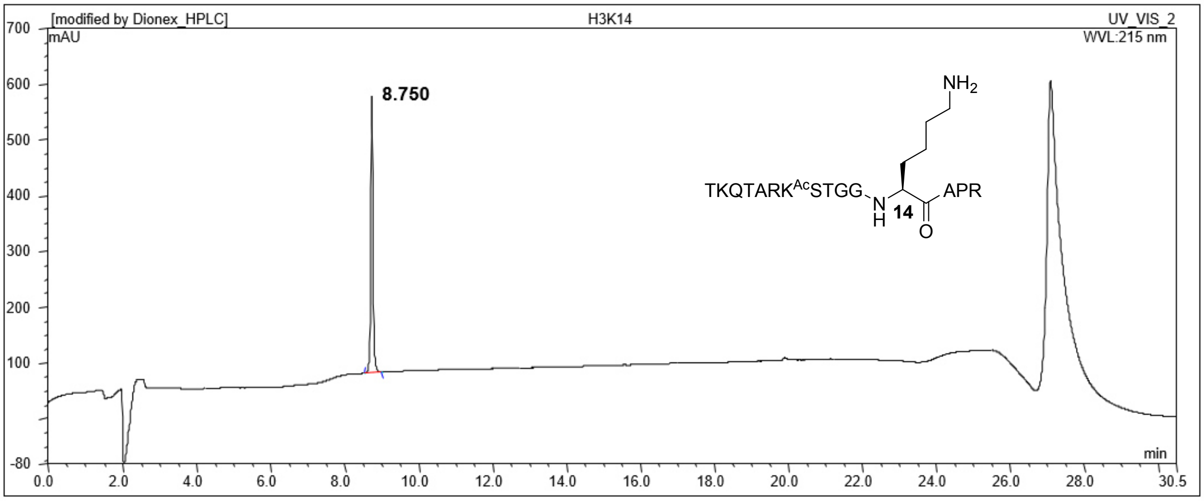

Figure S12. Analytical HPLC of the H3K14 peptide after RP-HPLC purification.

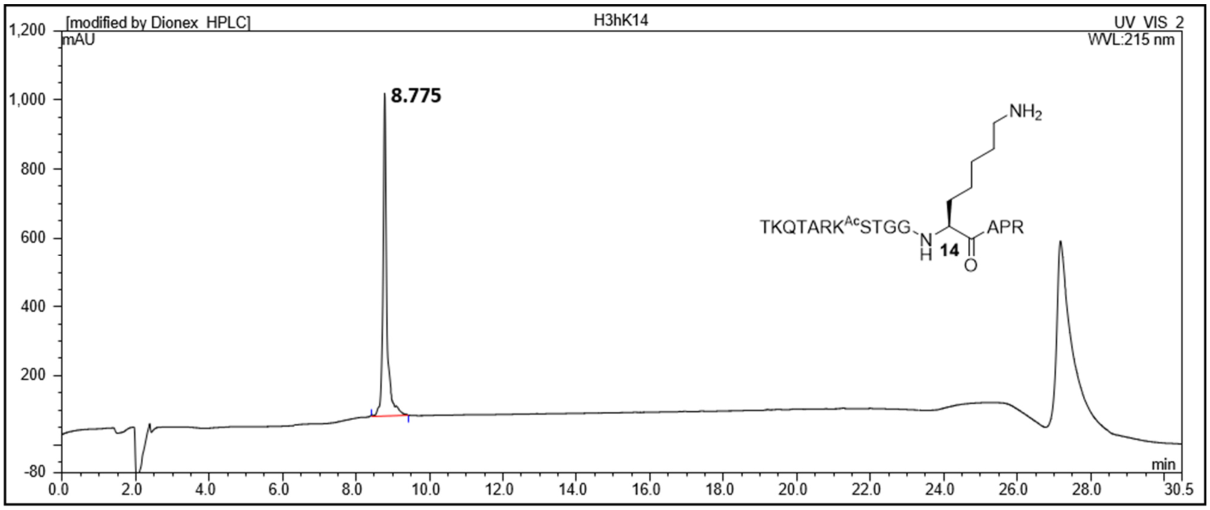

Figure S13. Analytical HPLC of the H3hK1 4 peptide after RP-HPLC purification.

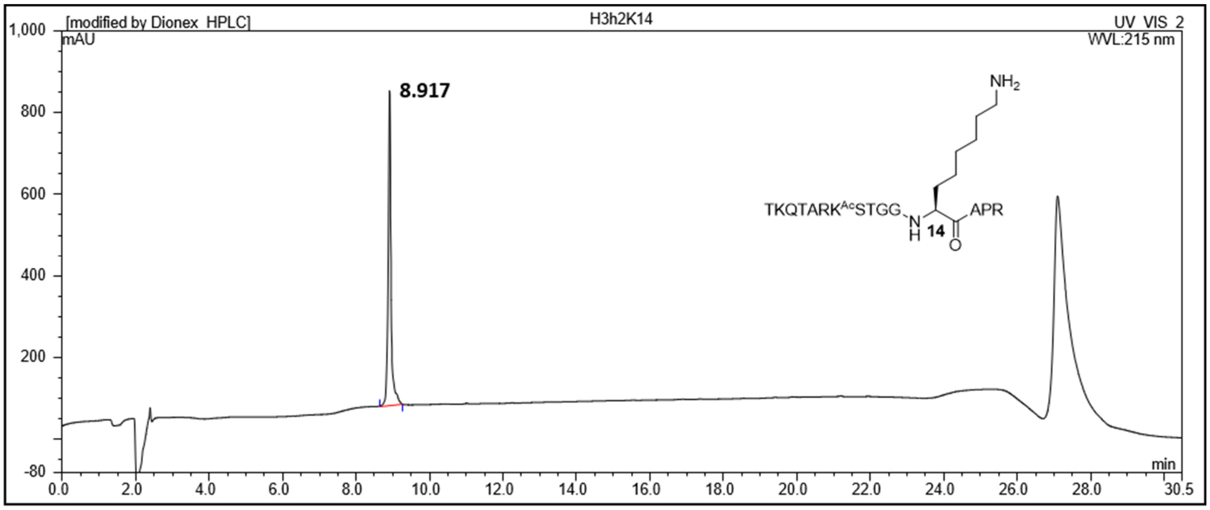

Figure S14. Analytical HPLC of the H3h$_2$K14 peptide after RP-HPLC purification.

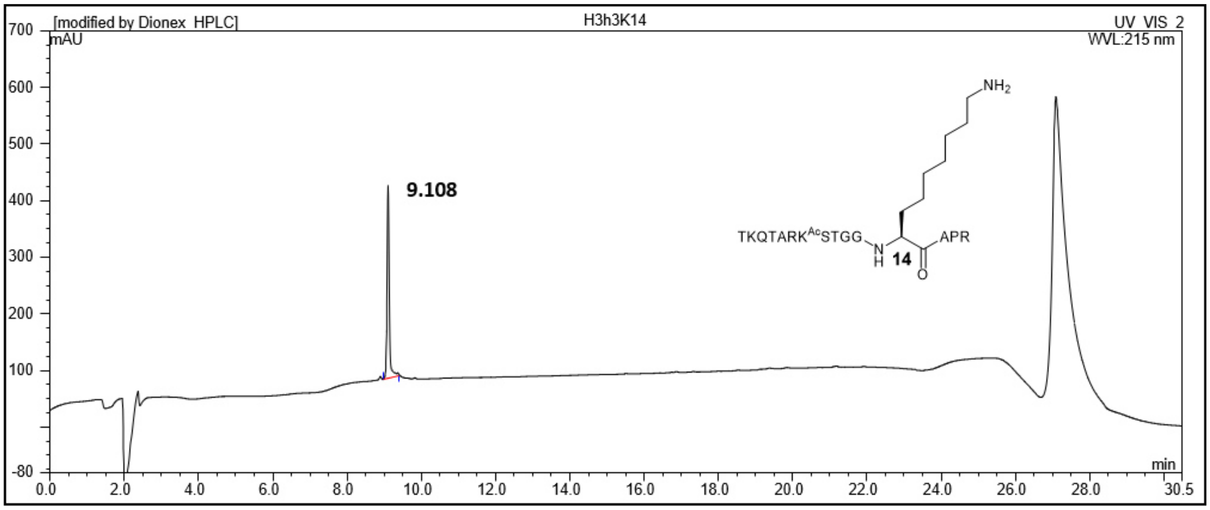

Figure S15. Analytical HPLC of the H3h_3K14 peptide after RP-HPLC purification.

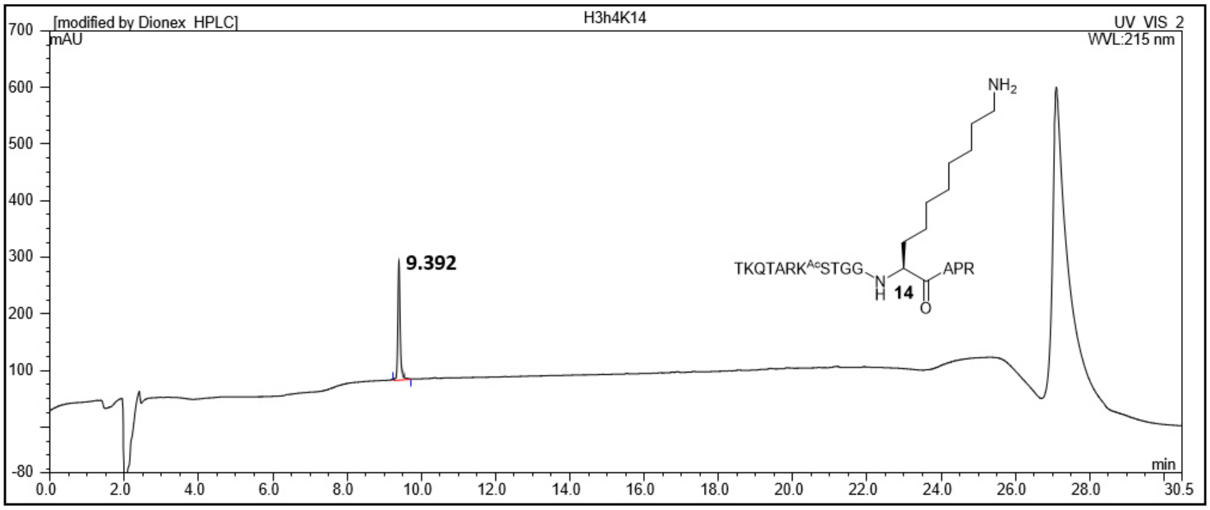

Figure S16. Analytical HPLC of the H3h_4K14 peptide after RP-HPLC purification.

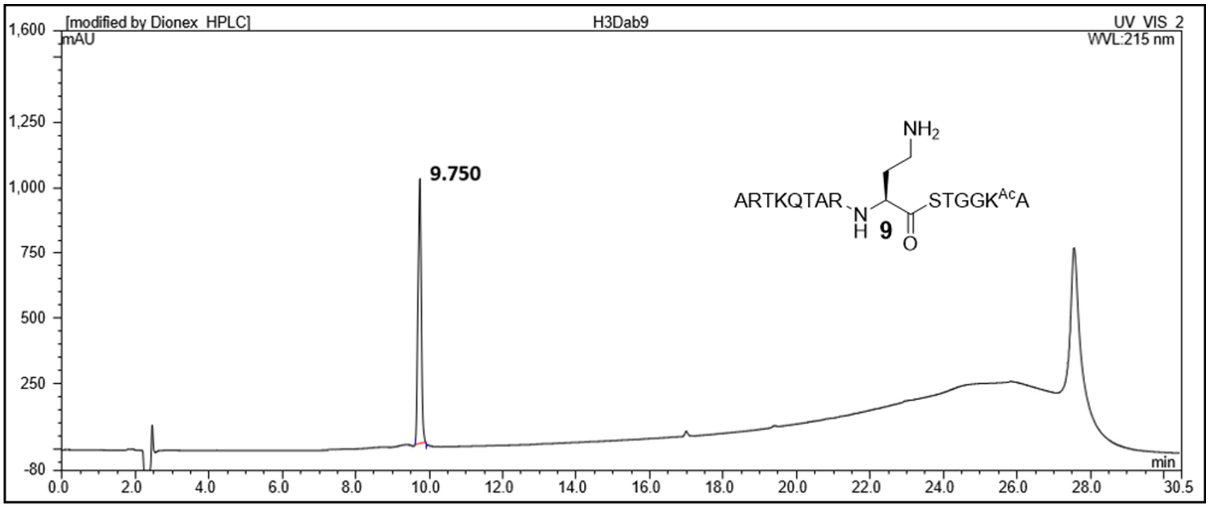

Figure S17. Analytical HPLC of the H3Dab9 peptide after RP-HPLC purification.

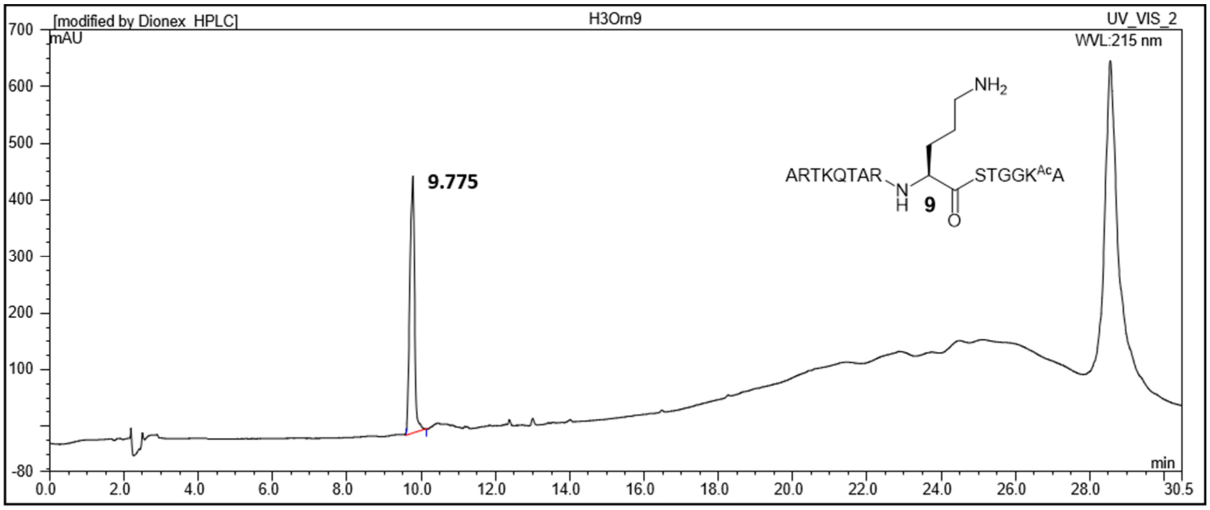

Figure S18. Analytical HPLC of the H3Orn9 peptide after RP-HPLC purification.

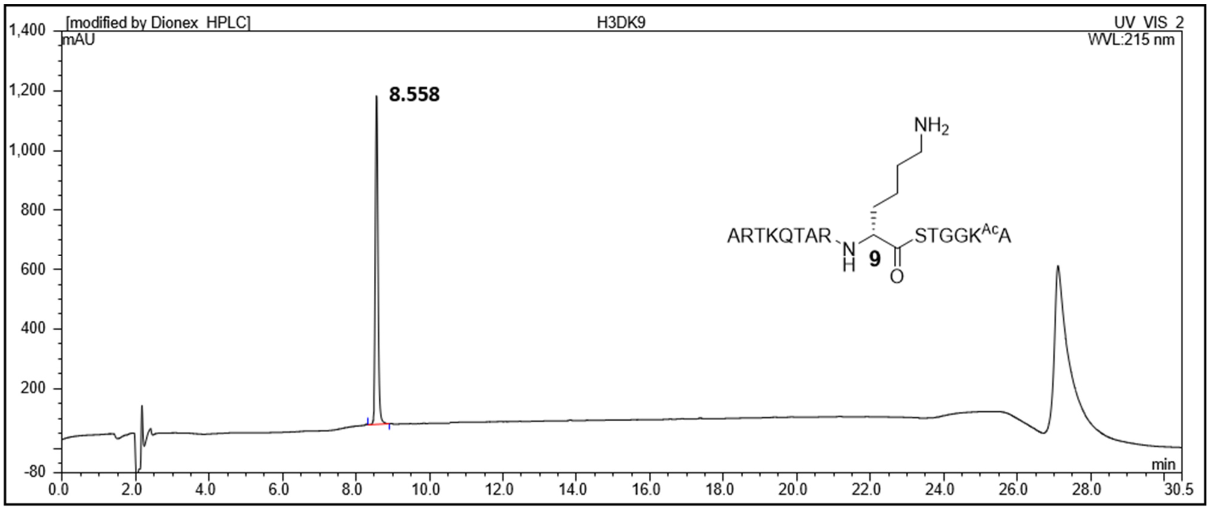

Figure S19. Analytical HPLC of the D-H3K9 peptide after RP-HPLC purification.

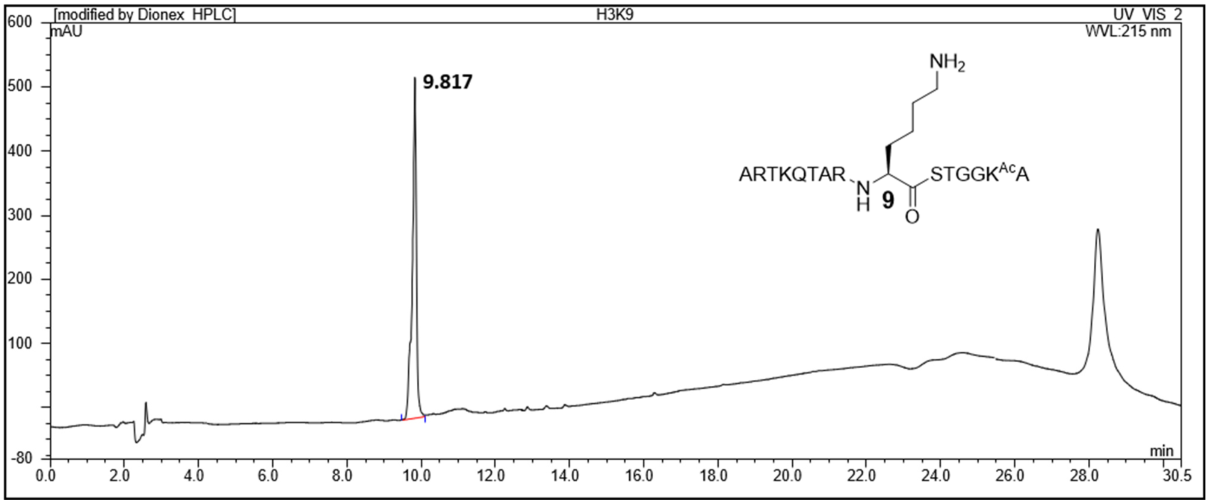

Figure S20. Analytical HPLC of the H3K9 peptide after RP-HPLC purification.

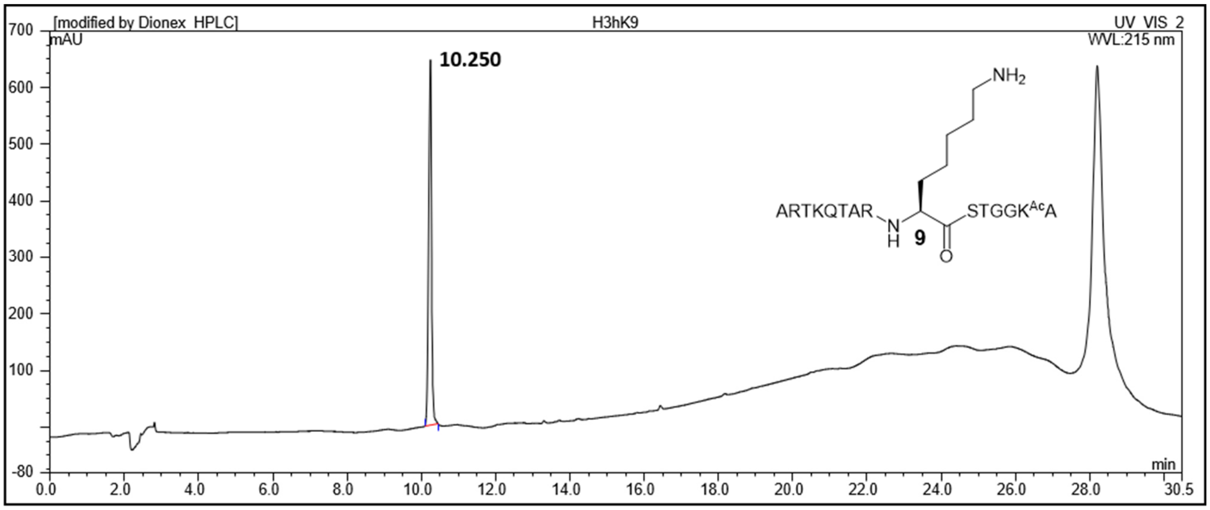

Figure S21. Analytical HPLC of the H3hK19 peptide after RP-HPLC purification.

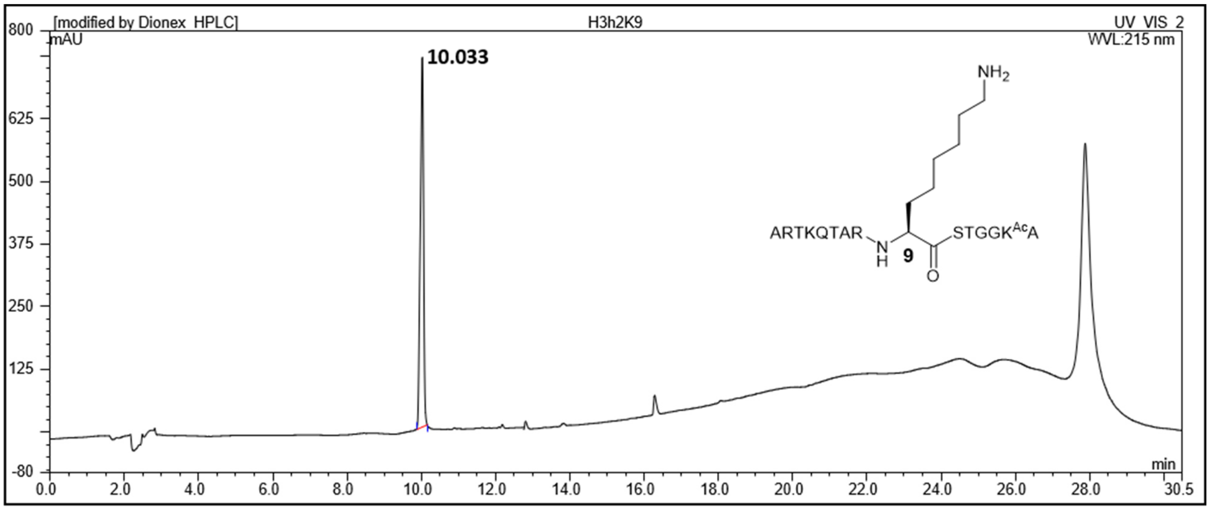

Figure S22. Analytical HPLC of the H3h$_2$K9 peptide after RP-HPLC purification.

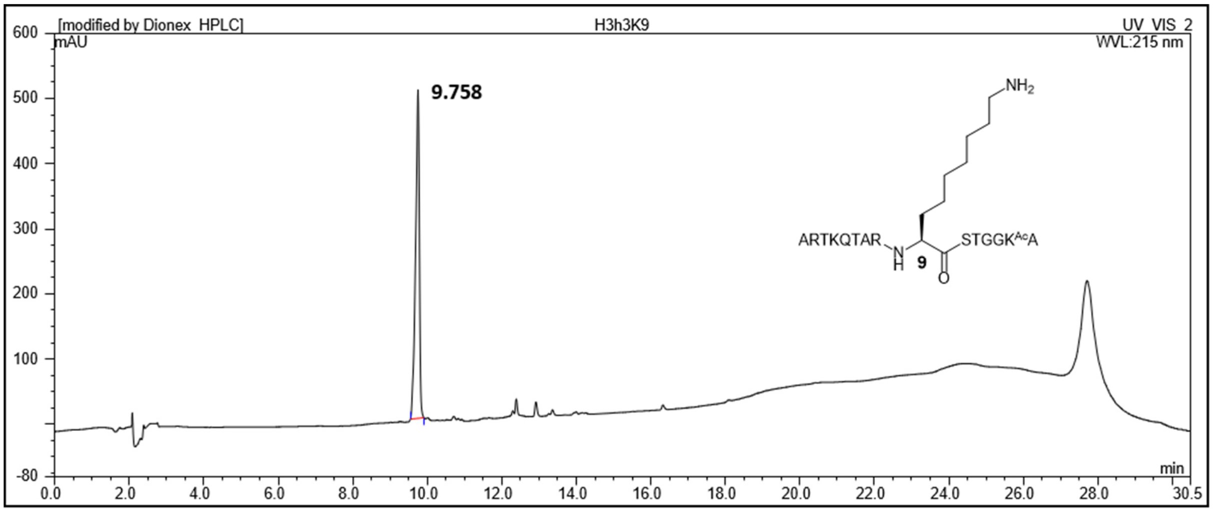

Figure S23. Analytical HPLC of the H3h$_3$K9 peptide after RP-HPLC purification.

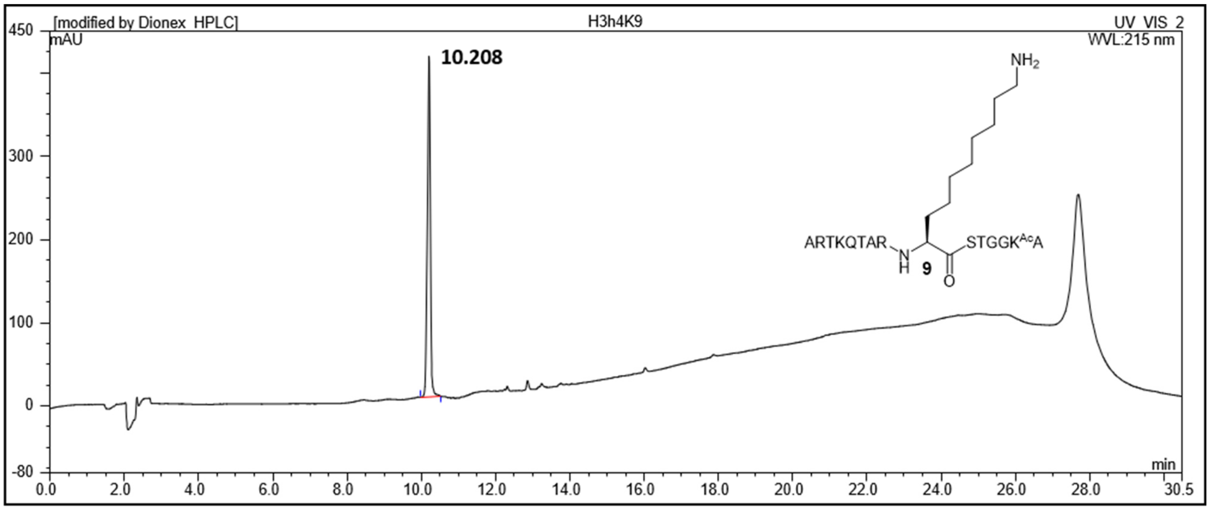

Figure S24. Analytical HPLC of the H3h$_4$K9 peptide after RP-HPLC purification.

6. Time course plot of KAT-catalyzed acetylation

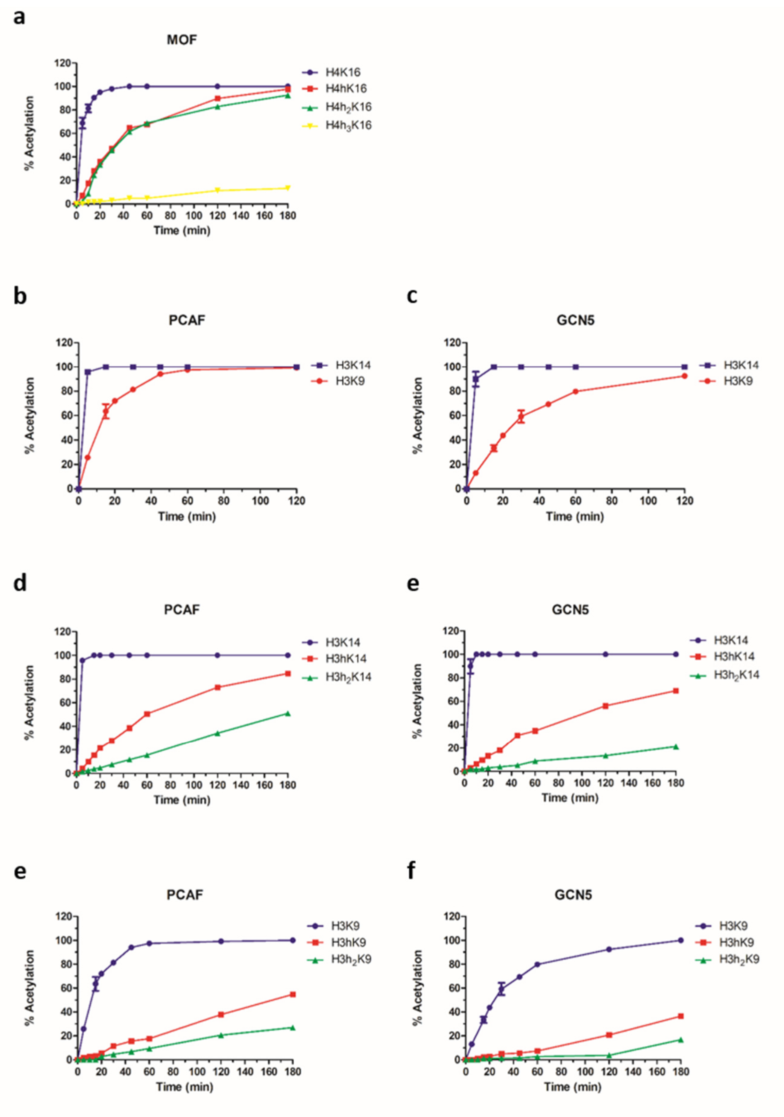

Figure S25. Time course analysis of KAT-catalyzed acetylation reactions of chain length extended lysine analogs. (a) MOF-catalyzed acetylation of lysine analogs on the H4K16 sequence. (b) PCAF-catalyzed comparison of acetylation for H3K14 and H3K9 peptides. (c) Gcn5-catalyzed comparison of acetylation for H3K14 and H3K9 peptides. (d) PCAF-catalyzed acetylation of lysine analogs on the H3K14 sequence. (e) PCAF-catalyzed acetylation of lysine analogs on the H3K9 sequence. (e) GCN5-catalyzed acetylation of lysine analogs on the H3K14 sequence. (f) GCN5-catalyzed acetylation of lysine analogs on the H3K9 sequence.

7. MALDI-TOF MS acetylation supporting figures

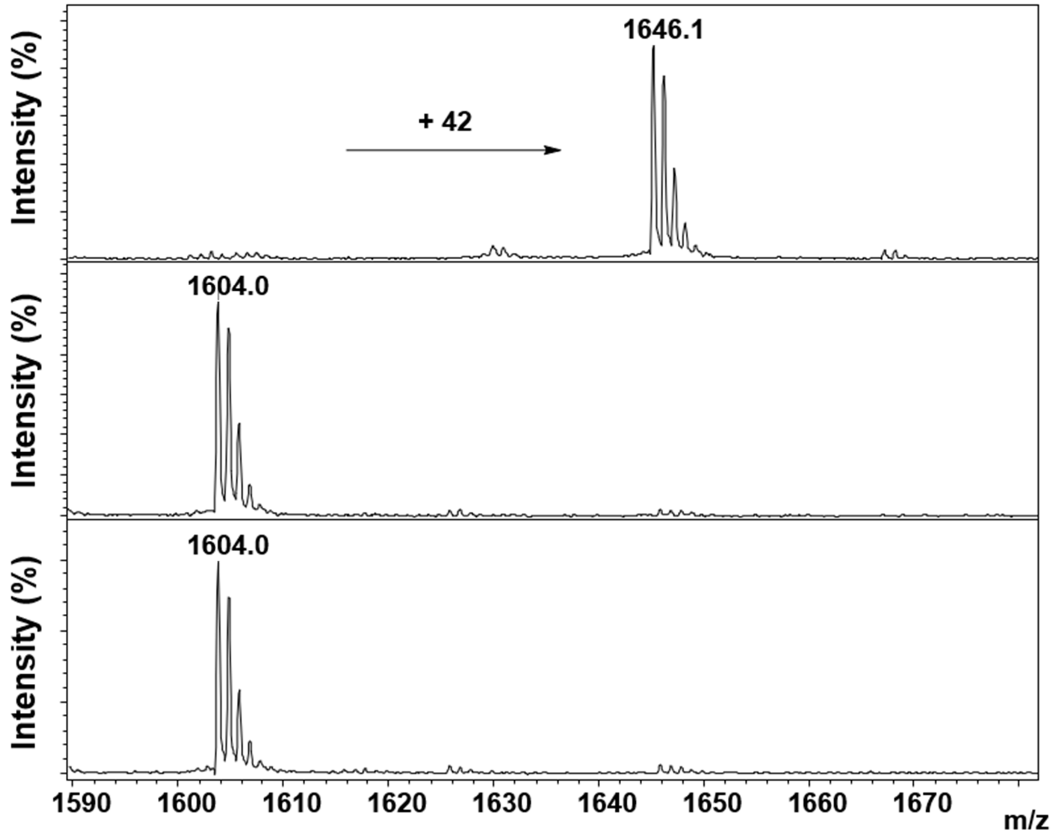

Figure S26. MALDI-TOF MS analysis of PCAF (2 μM)-catalyzed acetylation reaction of H3K9 peptide (100 μM) in the presence of AcCoA (300 μM) at 37 °C, after 3 h incubation (top panel). Control reactions after 3 h: absence of AcCoA (middle panel), absence of PCAF (bottom panel).

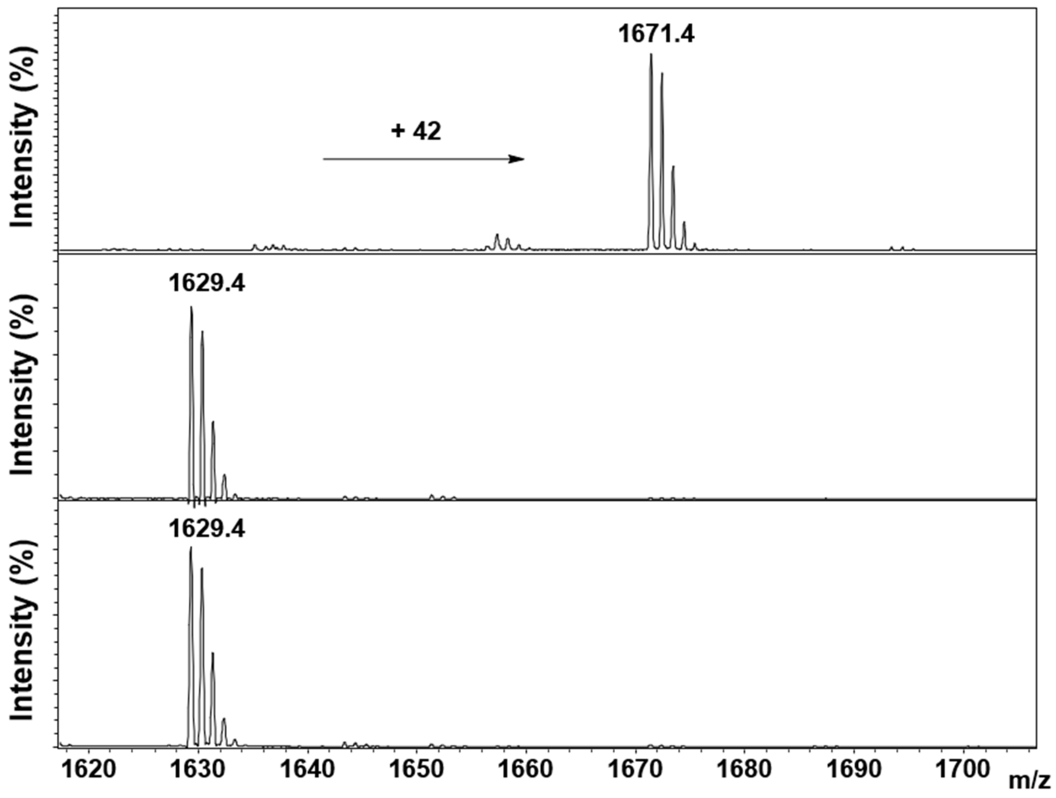

Figure S27. MALDI-OF MS analysis of GCN5 (2 μM)-catalyzed acetylation reaction of H3K14 peptide (100 μM) in the presence of AcCoA (300 μM) at 37 °C, after 3 h incubation (top panel). Control reactions after 3 h: absence of AcCoA (middle panel), absence of GCN5 (bottom panel).

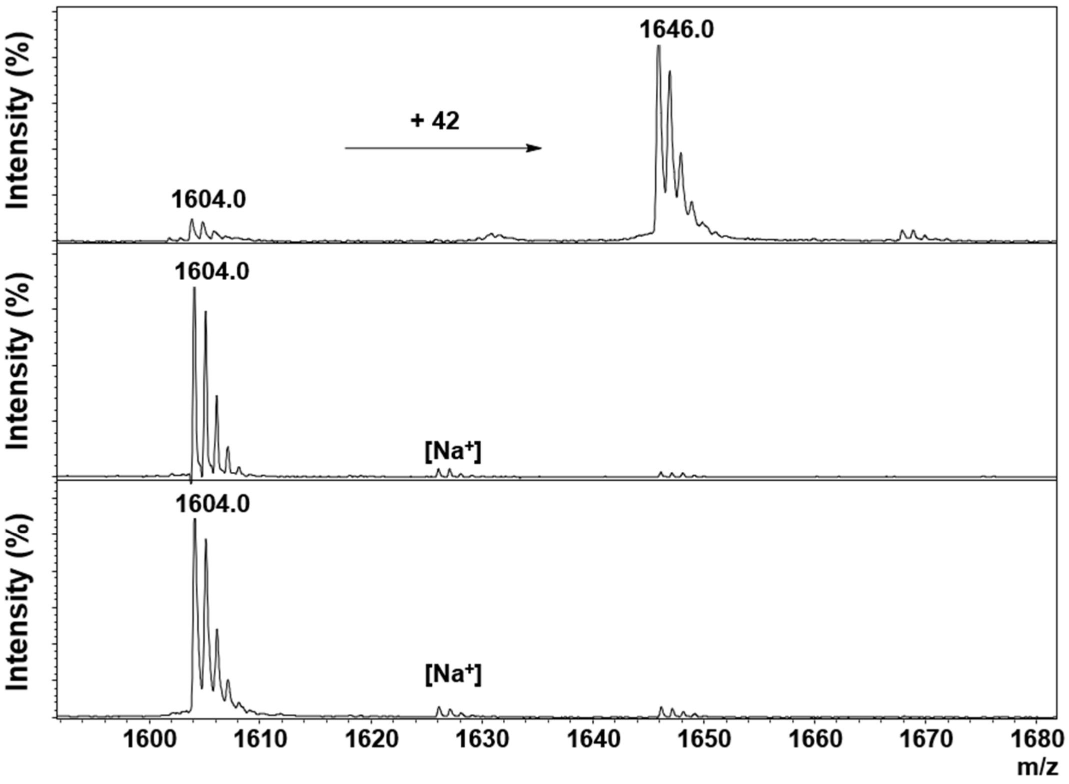

Figure S28. MALDI-OF MS analysis of GCN5 (2 μM)-catalyzed acetylation reaction of H3K9 peptide (100 μM) in the presence of AcCoA (300 μM) at 37 °C, after 3 h incubation (top panel). Control reactions after 3 h: absence of AcCoA (middle panel), absence of GCN5 (bottom panel).

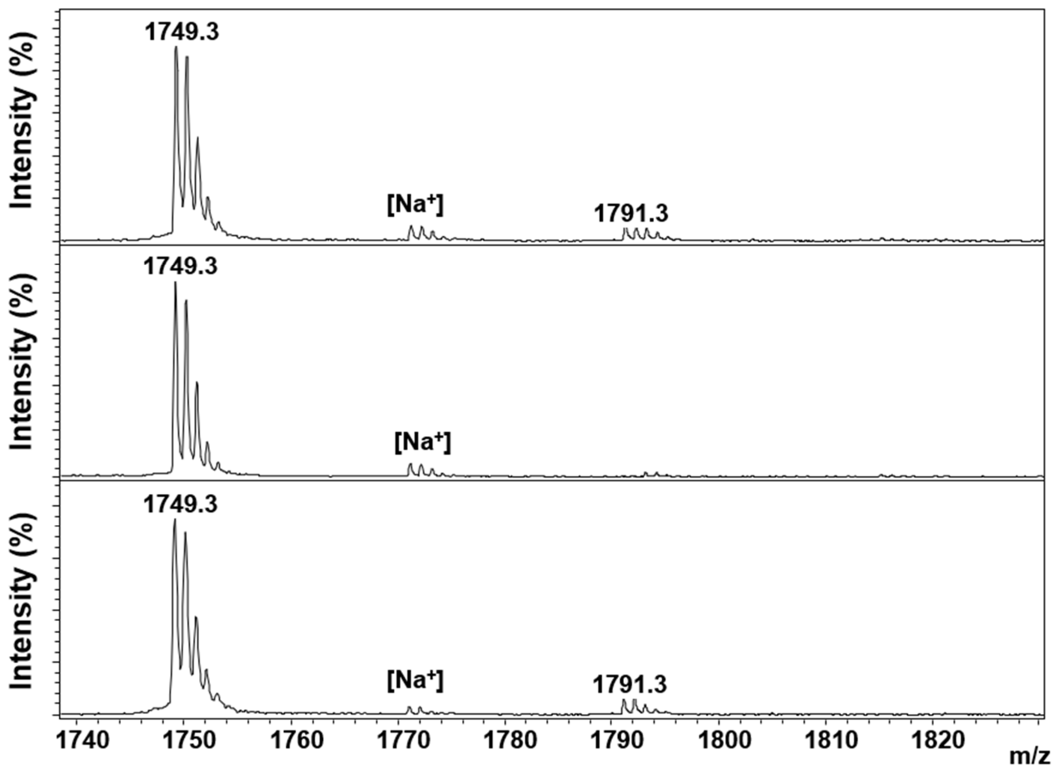

Figure S29. MALDI-TOF MS analysis of MOF (2 μM)-catalyzed acetylation reaction of D-H4K16 peptide (100 μM) in the presence of AcCoA (300 μM) at 37 °C, after 3 h incubation (top panel). Control reactions after 3 h: absence of AcCoA (middle panel), absence of MOF (bottom panel).

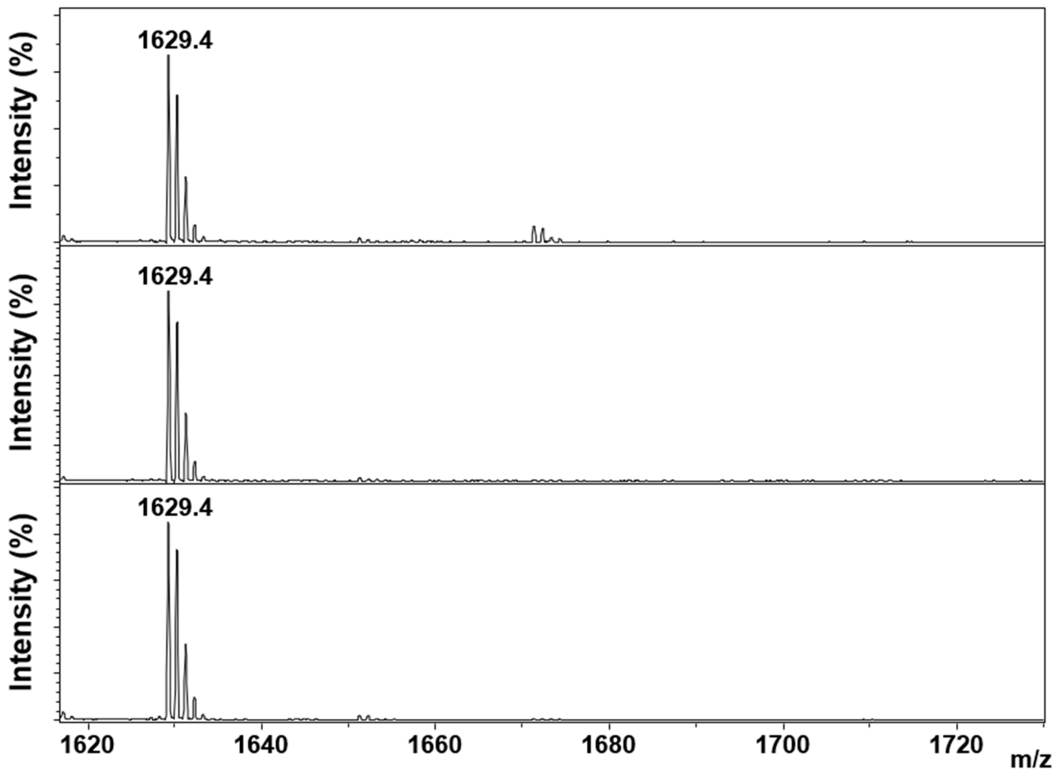

Figure S30. MALDI-TOF MS analysis of PCAF (2 μM)-catalyzed acetylation reaction of D-H3K14 peptide (100 μM) in the presence of AcCoA (300 μM) at 37 °C, after 3 h incubation (top panel). Control reactions after 3 h: absence of AcCoA (middle panel), absence of PCAF (bottom panel).

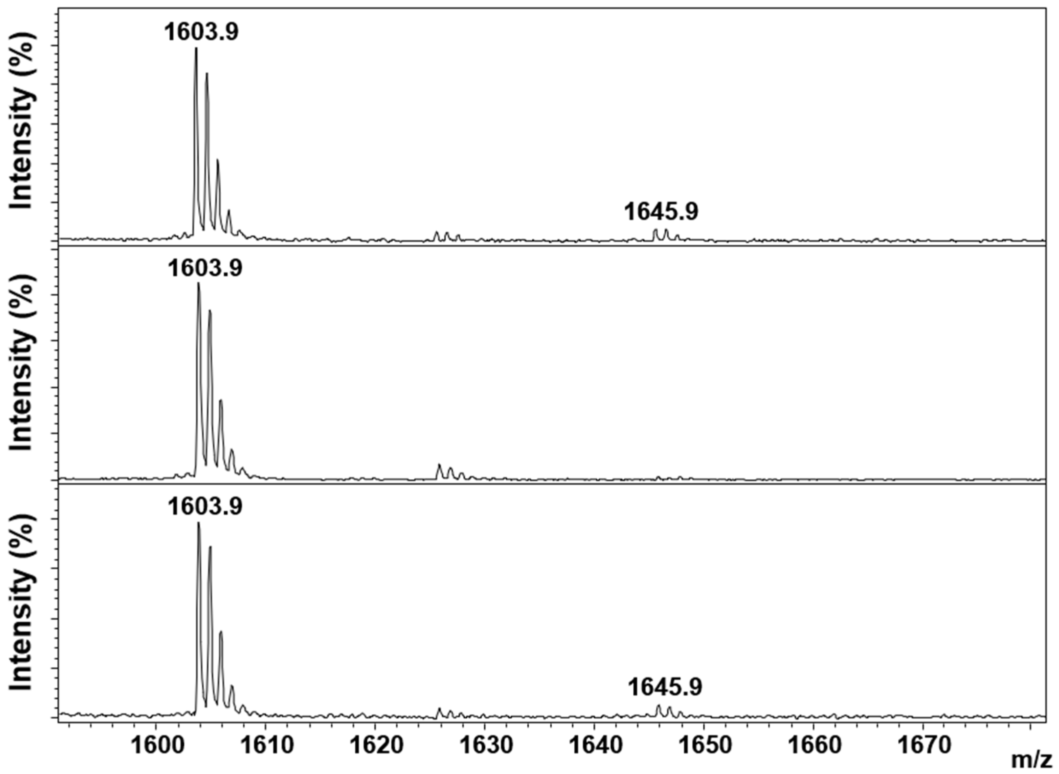

Figure S31. MALDI-TOF MS analysis of PCAF (2 μM)-catalyzed acetylation reaction of D-H3K9 peptide (100 μM) in the presence of AcCoA (300 μM) at 37 °C, after 3 h incubation (top panel). Control reactions after 3 h: absence of AcCoA (middle panel), absence of PCAF (bottom panel).

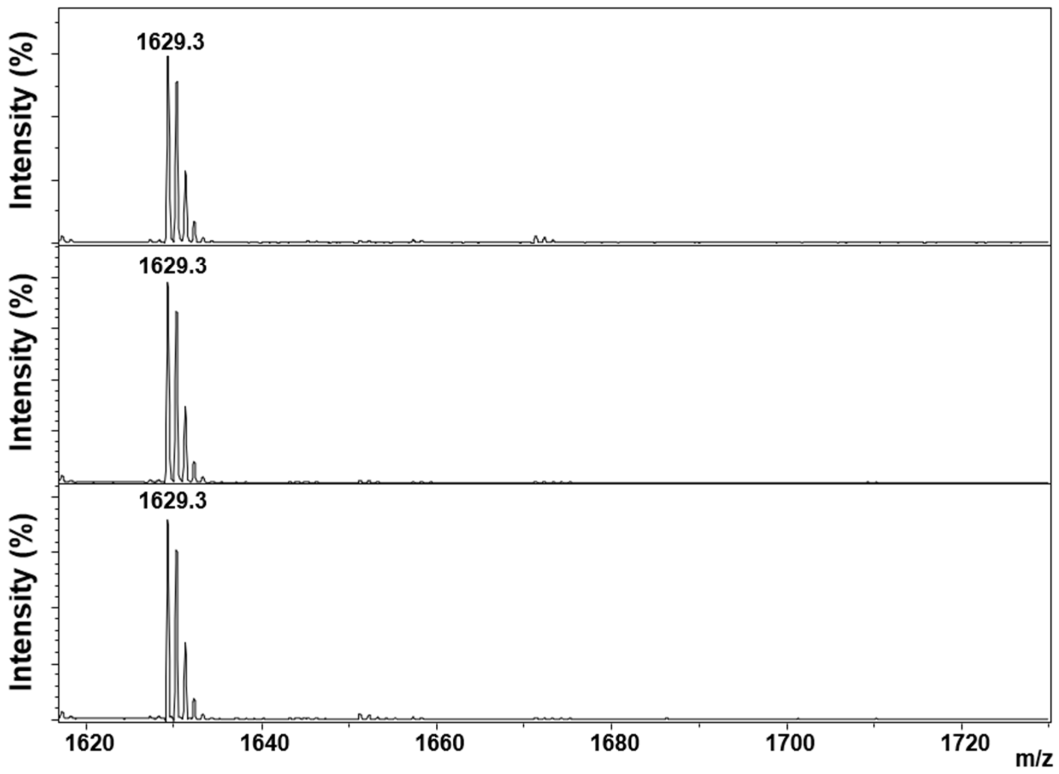

Figure S32. MALDI-OF MS analysis of GCN5 (2 μM)-catalyzed acetylation reaction of D-H3K14 peptide (100 μM) in the presence of AcCoA (300 μM) at 37 °C, after 3 h incubation (top panel). Control reactions after 3 h: absence of AcCoA (middle panel), absence of GCN5 (bottom panel).

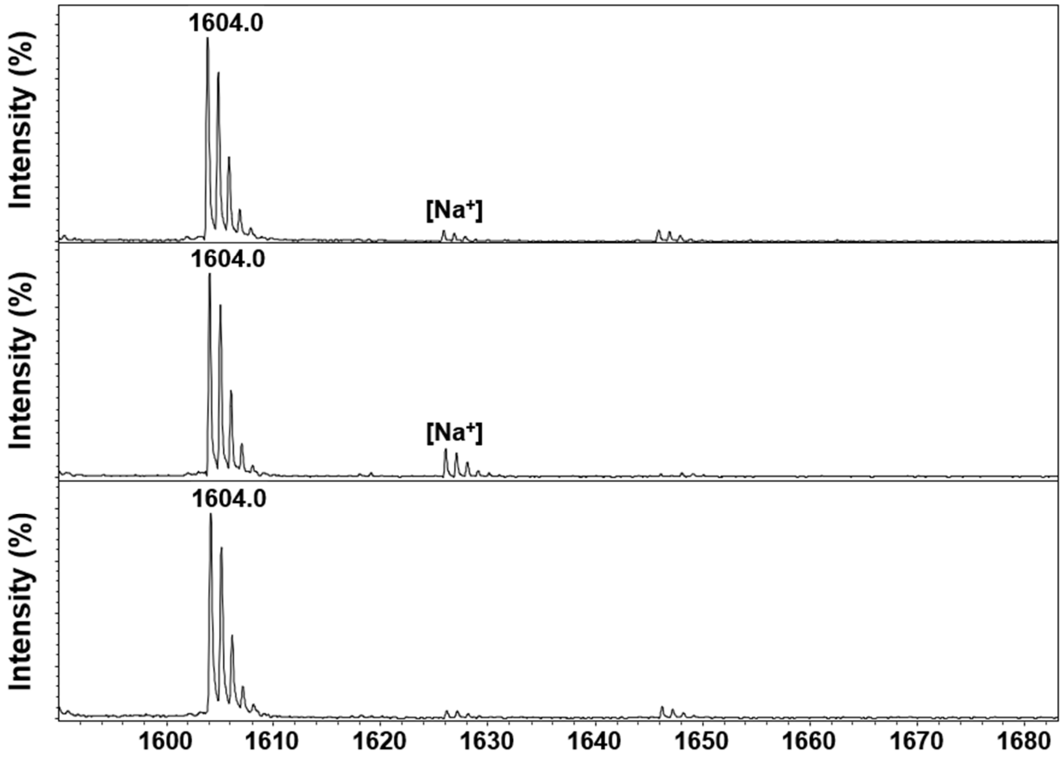

Figure S33. MALDI-TOF MS analysis of GCN5 (2 μM)-catalyzed acetylation reaction of D-H3K9 peptide (100 μM) in the presence of AcCoA (300 μM) at 37 °C, after 3 h incubation (top panel). Control reactions after 3 h: absence of AcCoA (middle panel), absence of GCN5 (bottom panel).

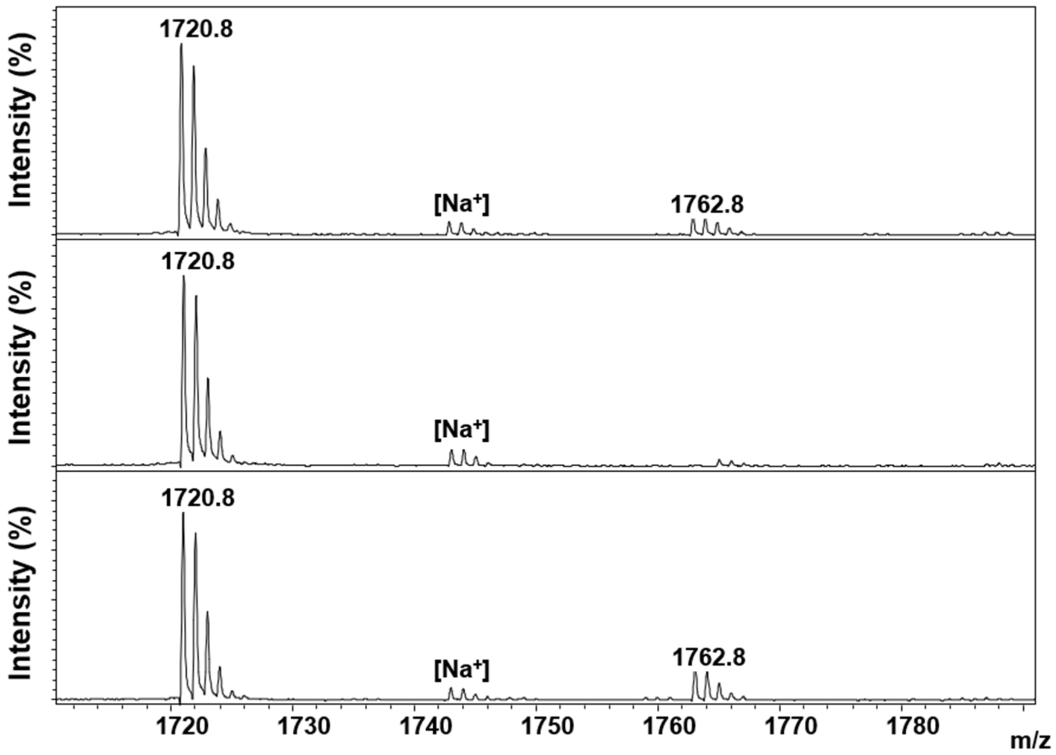

Figure S34. MALDI-TOF MS analysis of MOF (2 μM)-catalyzed acetylation reaction of H4Dab16 peptide (100 μM) in the presence of AcCoA (300 μM) at 37 °C, after 3 h incubation (top panel). Control reactions after 3 h: absence of AcCoA (middle panel), absence of MOF (bottom panel).

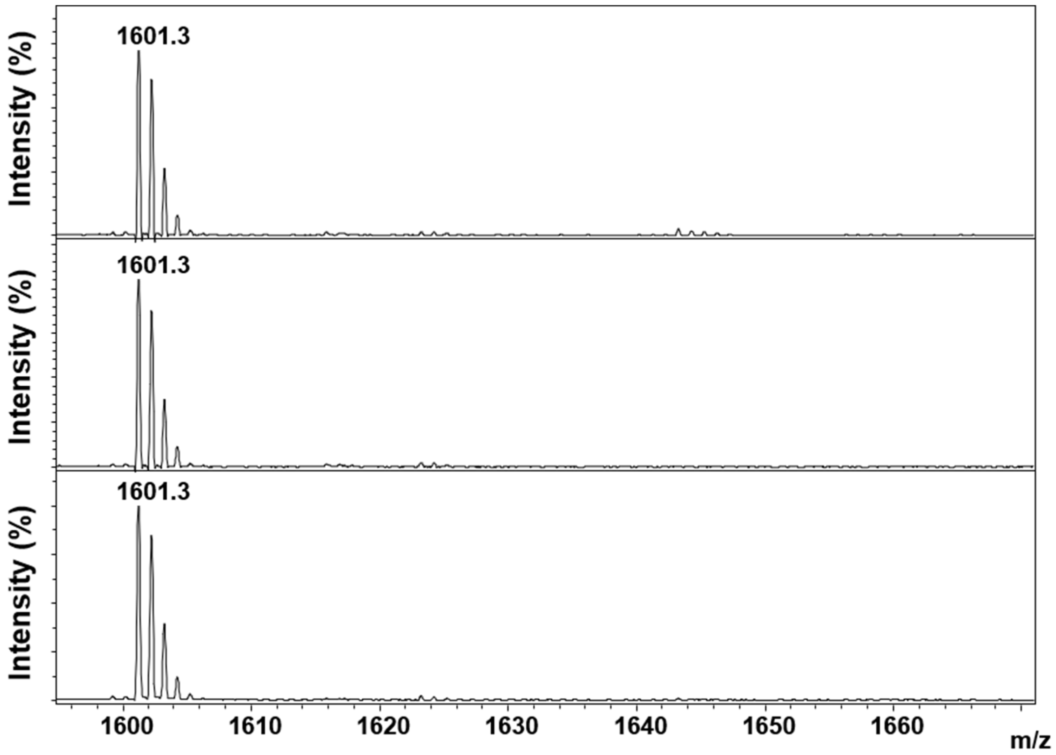

Figure S35. MALDI-TOF MS analysis of PCAF (2 μM)-catalyzed acetylation reaction of H3Dab14 peptide (100 μM) in the presence of AcCoA (300 μM) at 37 °C, after 3 h incubation (top panel). Control reactions after 3 h: absence of AcCoA (middle panel), absence of PCAF (bottom panel).

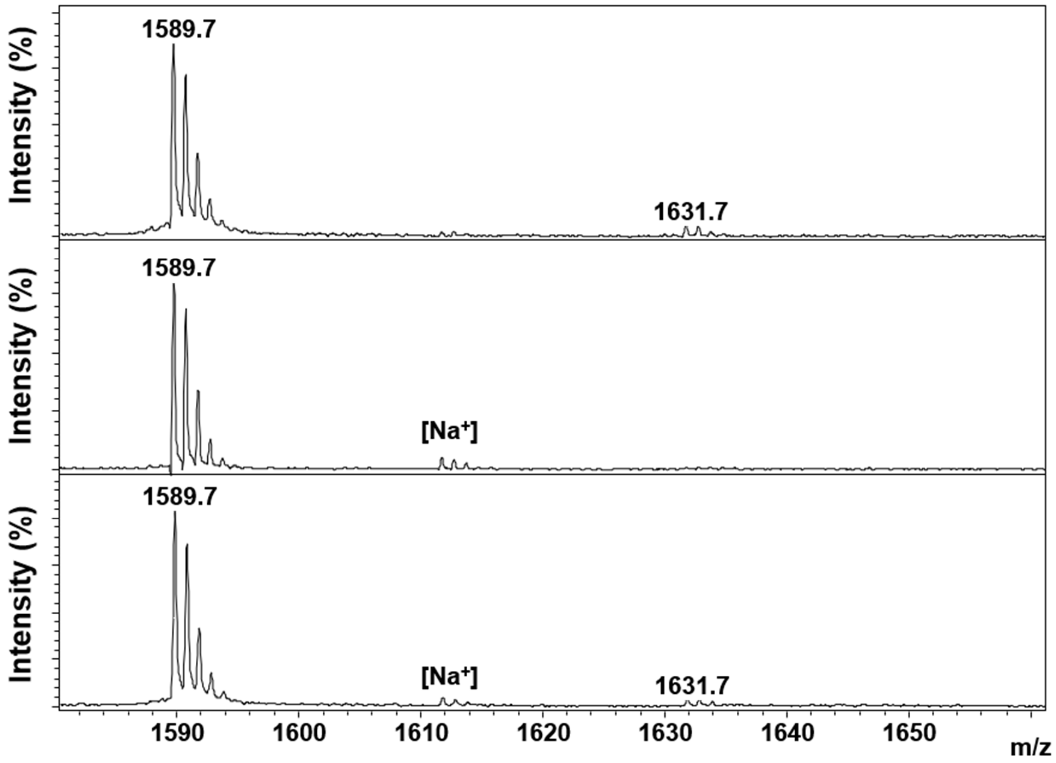

Figure S36. MALDI-TOF MS analysis of PCAF (2 μM)-catalyzed acetylation reaction of H3Orn9 peptide (100 μM) in the presence of AcCoA (300 μM) at 37 °C, after 3 h incubation (top panel). Control reactions after 3 h: absence of AcCoA (middle panel), absence of PCAF (bottom panel).

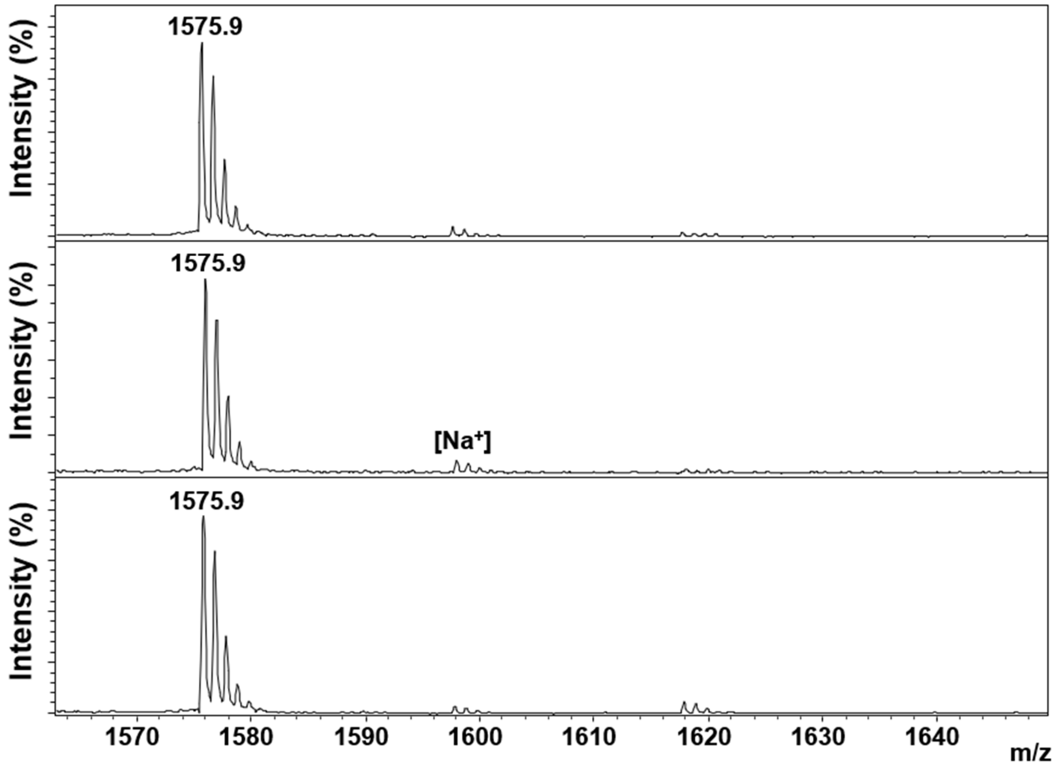

Figure S37. MALDI-TOF MS analysis of PCAF (2 μM)-catalyzed acetylation reaction of H3Dab9 peptide (100 μM) in the presence of AcCoA (300 μM) at 37 °C, after 3 h incubation (top panel). Control reactions after 3 h: absence of AcCoA (middle panel), absence of PCAF (bottom panel).

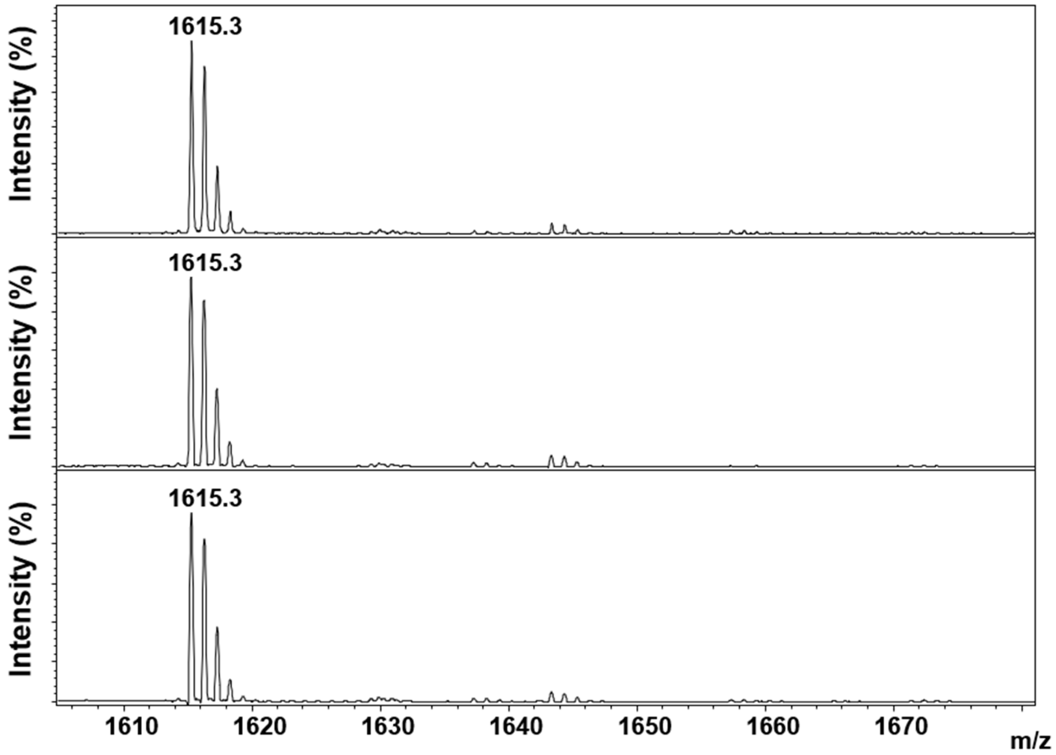

Figure S38. MALDI-TOF MS analysis of GCN5 (2 μM)-catalyzed acetylation reaction of H3Orn14 peptide (100 μM) in the presence of AcCoA (300 μM) at 37 °C, after 3 h incubation (top panel). Control reactions after 3 h: absence of AcCoA (middle panel), absence of GCN5 (bottom panel).

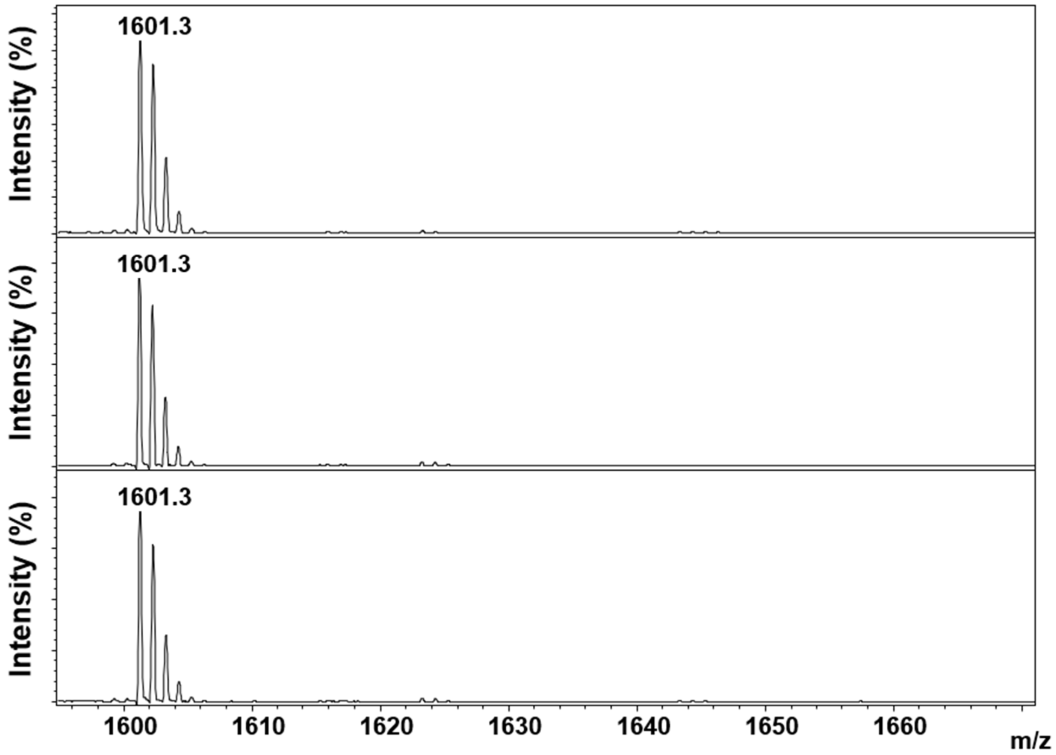

Figure S39. MALDI-TOF MS analysis of GCN5 (2 μM)-catalyzed acetylation reaction of H3Dab14 peptide (100 μM) in the presence of AcCoA (300 μM) at 37 °C, after 3 h incubation (top panel). Control reactions after 3 h: absence of AcCoA (middle panel), absence of GCN5 (bottom panel).

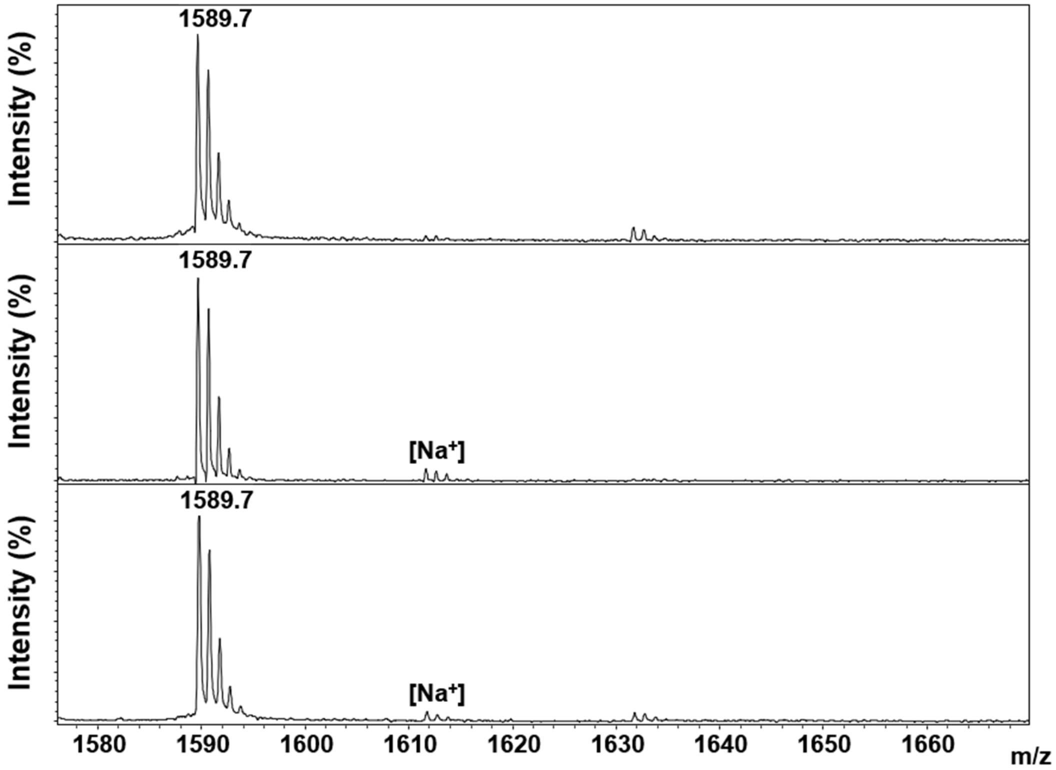

Figure S40. MALDI-TOF MS analysis of GCN5 (2 μM)-catalyzed acetylation reaction of H3Orn9 peptide (100 μM) in the presence of AcCoA (300 μM) at 37 °C, after 3 h incubation (top panel). Control reactions after 3 h: absence of AcCoA (middle panel), absence of GCN5 (bottom panel).

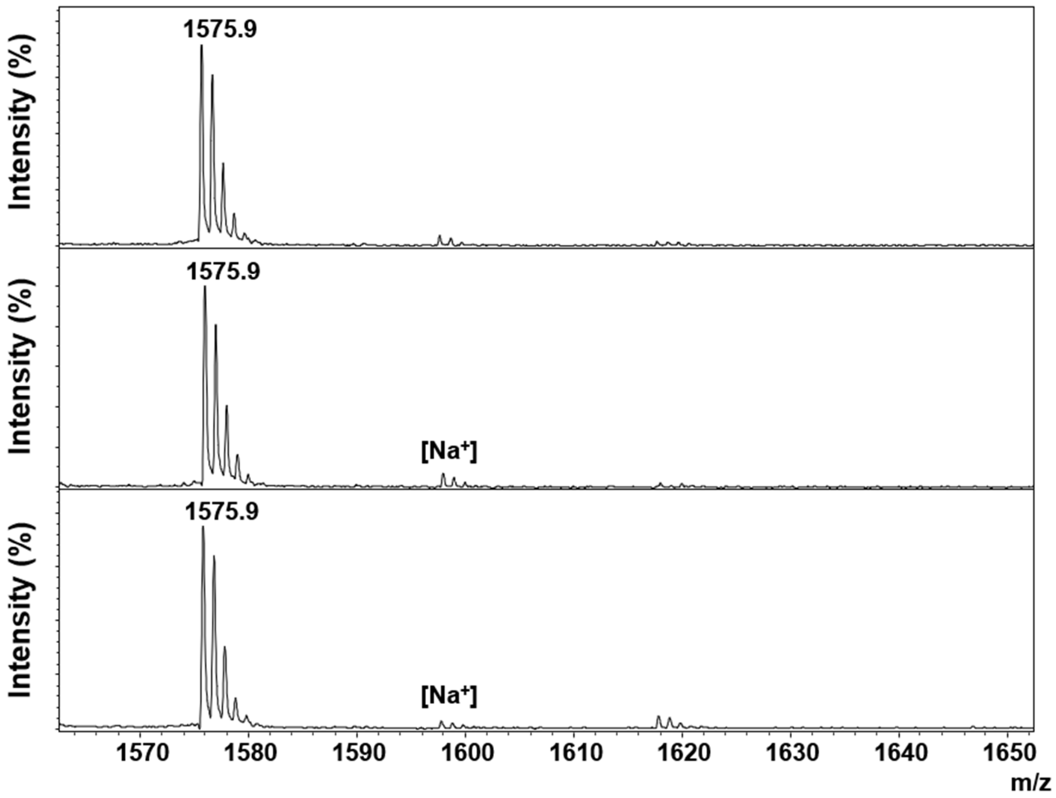

Figure S41. MALDI-TOF MS analysis of GCN5 (2 μM)-catalyzed acetylation reaction of H3Dab9 peptide (100 μM) in the presence of AcCoA (300 μM) at 37 °C, after 3 h incubation (top panel). Control reactions after 3 h: absence of AcCoA (middle panel), absence of GCN5 (bottom panel).

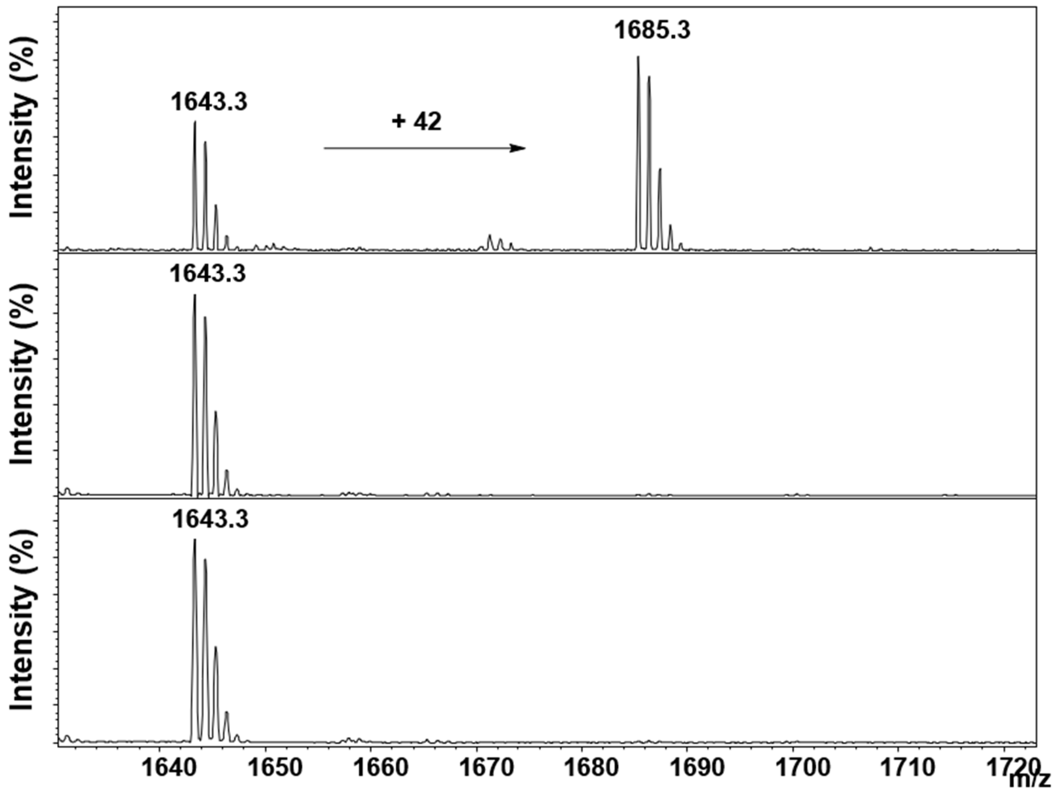

Figure S42. MALDI-TOF MS analysis of GCN5 (2 μM)-catalyzed acetylation reaction of H3hK14 peptide (100 μM) in the presence of AcCoA (300 μM) at 37 °C, after 3 h incubation (top panel). Control reactions after 3 h: absence of AcCoA (middle panel), absence of GCN5 (bottom panel).

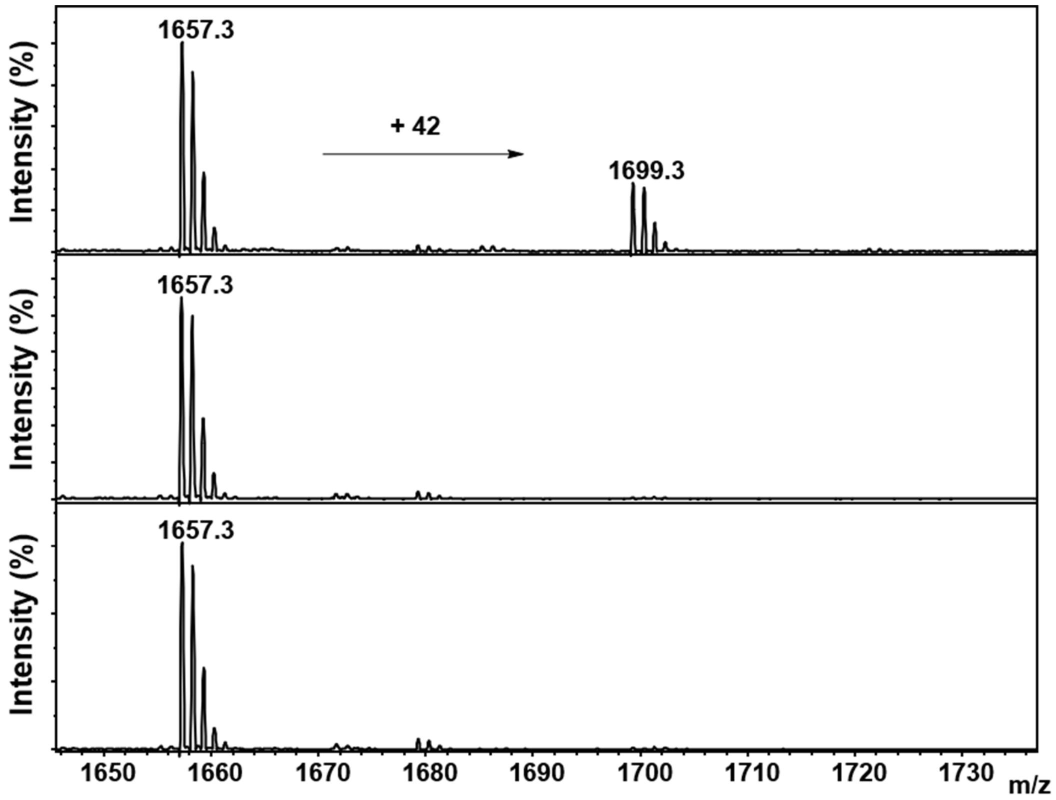

Figure S43. MALDI-TOF MS analysis of GCN5 (2 μM)-catalyzed acetylation reaction of H3h$_2$K14 peptide (100 μM) in the presence of AcCoA (300 μM) at 37 °C, after 3 h incubation (top panel). Control reactions after 3 h: absence of AcCoA (middle panel), absence of GCN5 (bottom panel).

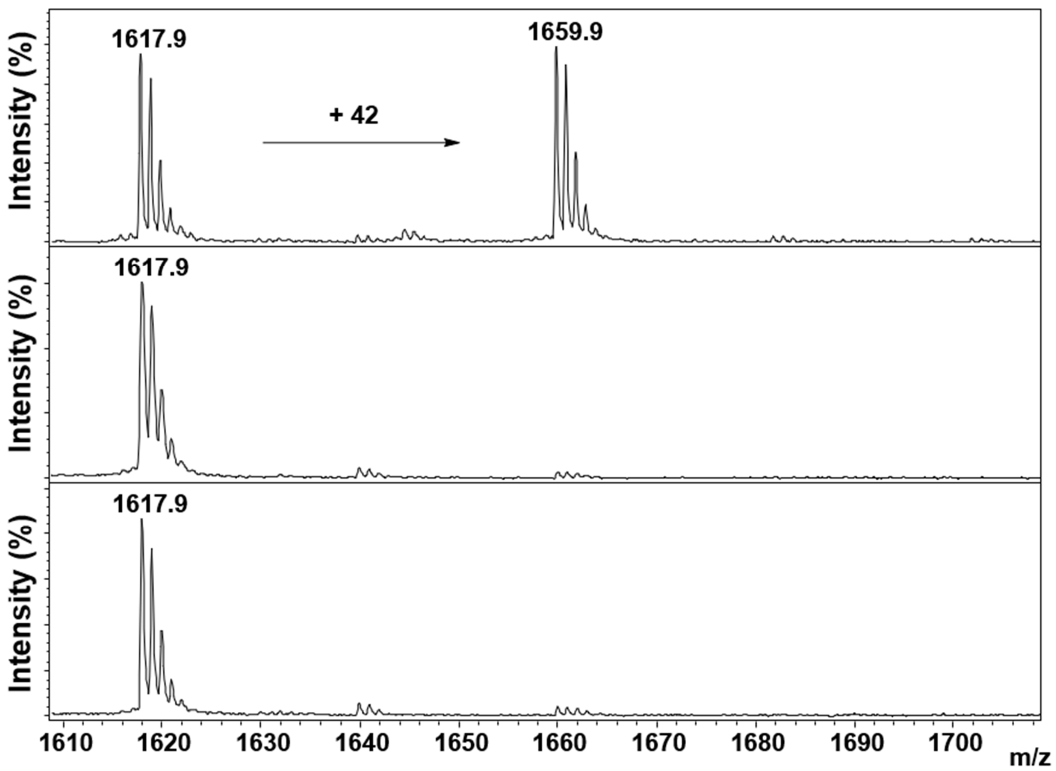

Figure S44. MALDI-TOF MS analysis of PCAF (2 μM)-catalyzed acetylation reaction of H3hK9 peptide (100 μM) in the presence of AcCoA (300 μM) at 37 °C, after 3 h incubation (top panel). Control reactions after 3 h: absence of AcCoA (middle panel), absence of PCAF (bottom panel).

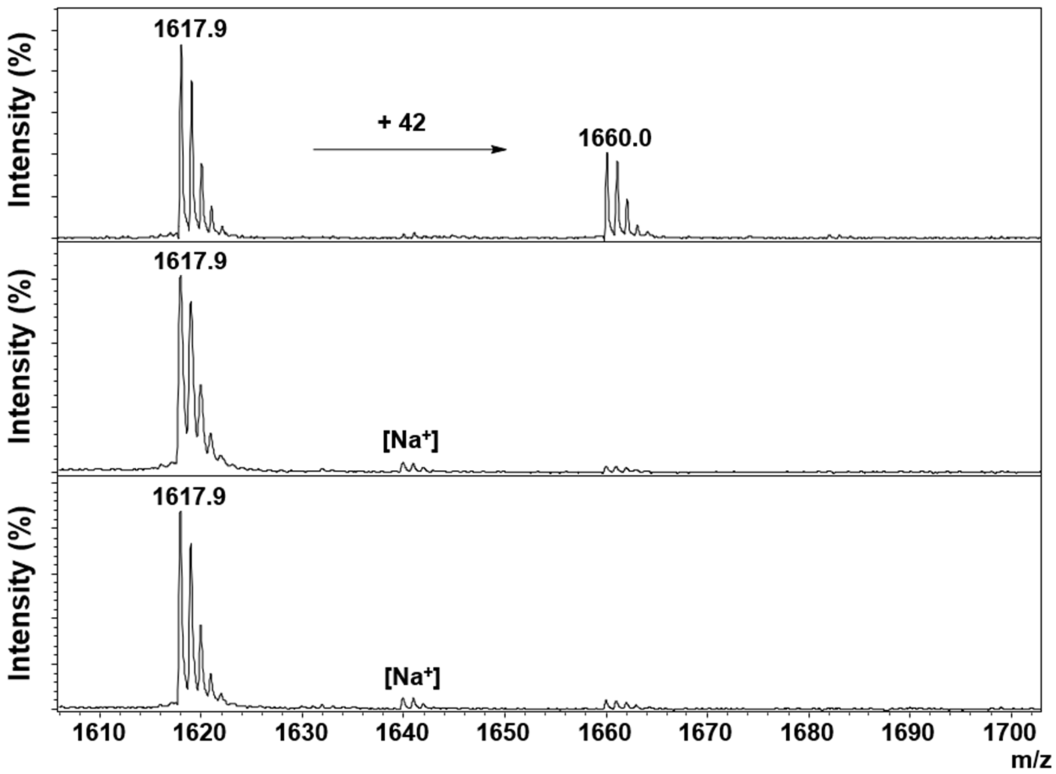

Figure S45. MALDI-TOF MS analysis of GCN5 (2 μM)-catalyzed acetylation reaction of H3hK9 peptide (100 μM) in the presence of AcCoA (300 μM) at 37 °C, after 3 h incubation (top panel). Control reactions after 3 h: absence of AcCoA (middle panel), absence of GCN5 (bottom panel).

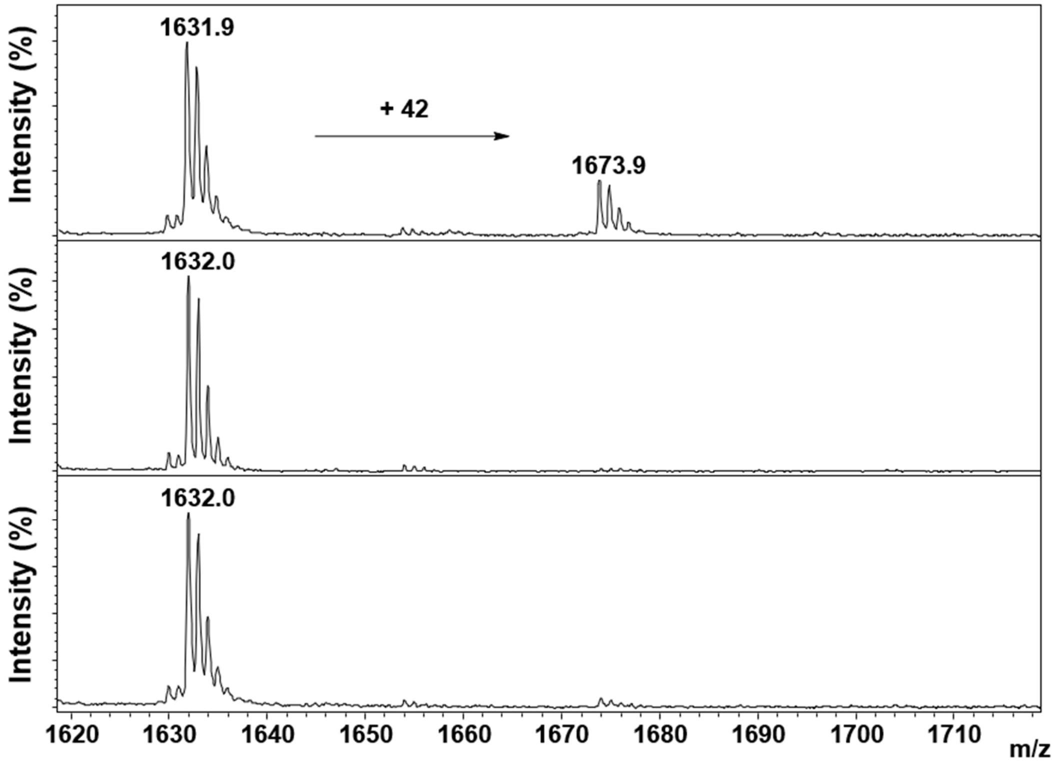

Figure S46. MALDI-TOF MS analysis of PCAF (2 μM)-catalyzed acetylation reaction of $H3h_2K9$ peptide (100 μM) in the presence of AcCoA (300 μM) at 37 °C, after 3 h incubation (top panel). Control reactions after 3 h: absence of AcCoA (middle panel), absence of PCAF (bottom panel).

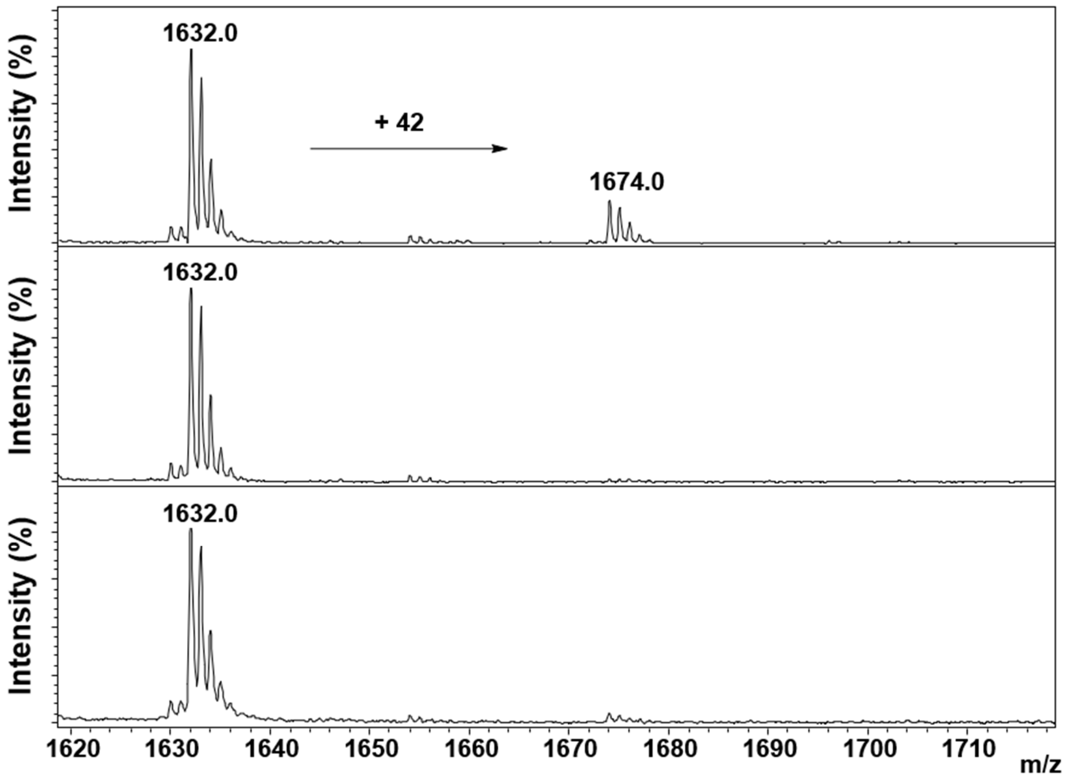

Figure S47. MALDI-TOF MS analysis of GCN5 (2 μM)-catalyzed acetylation reaction of $H3h_2K9$ peptide (100 μM) in the presence of AcCoA (300 μM) at 37 °C, after 3 h incubation (top panel). Control reactions after 3 h: absence of AcCoA (middle panel), absence of GCN5 (bottom panel).

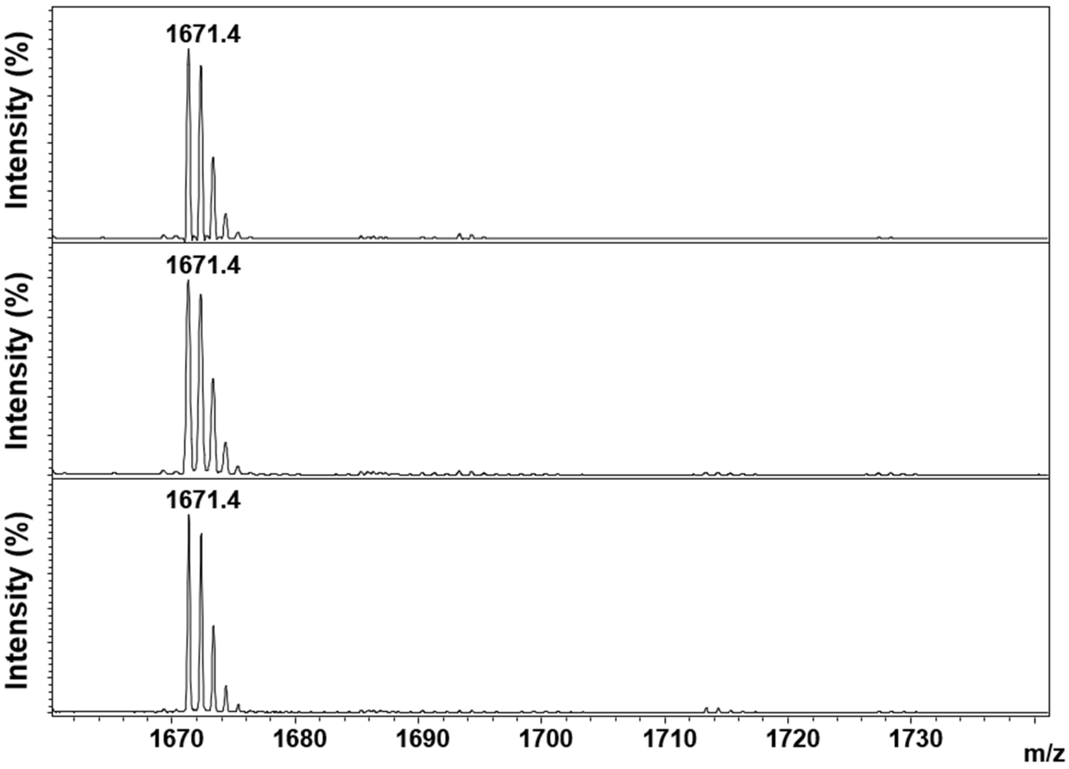

Figure S48. MALDI-TOF MS analysis of GCN5 (2 μM)-catalyzed acetylation reaction of H3h$_3$K14 peptide (100 μM) in the presence of AcCoA (300 μM) at 37 °C, after 3 h incubation (top panel). Control reactions after 3 h: absence of AcCoA (middle panel), absence of GCN5 (bottom panel).

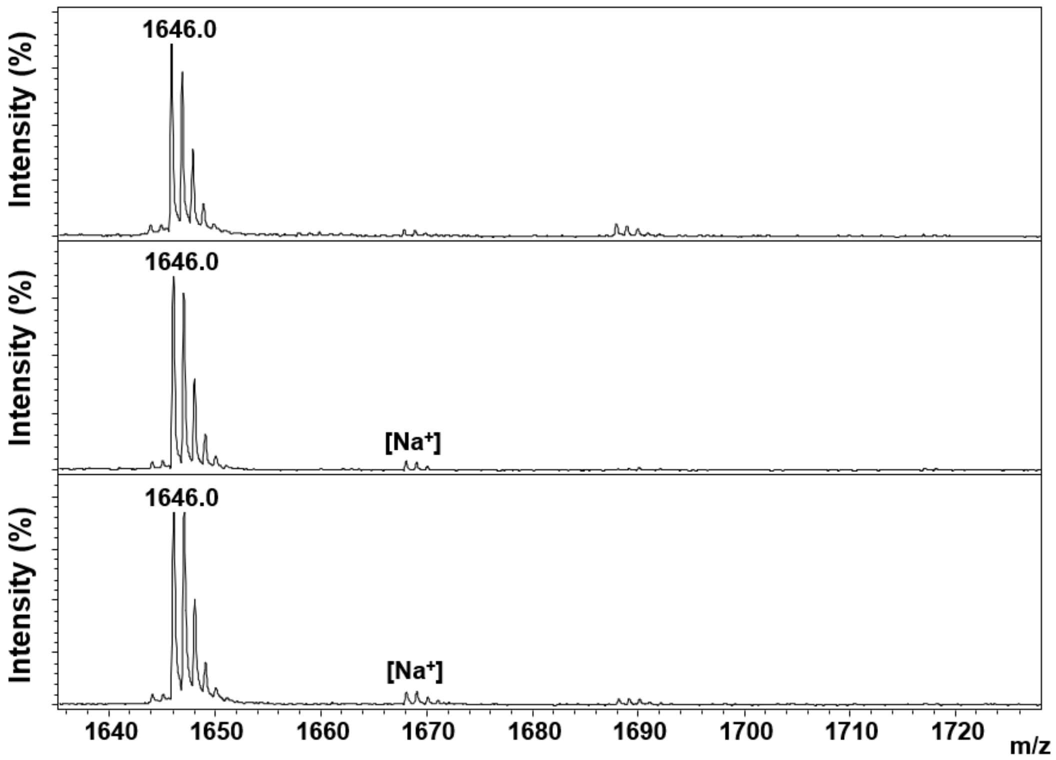

Figure S49. MALDI-TOF MS analysis of PCAF (2 μM)-catalyzed acetylation reaction of H3h$_3$K9 peptide (100 μM) in the presence of AcCoA (300 μM) at 37 °C, after 3 h incubation (top panel). Control reactions after 3 h: absence of AcCoA (middle panel), absence of PCAF (bottom panel).

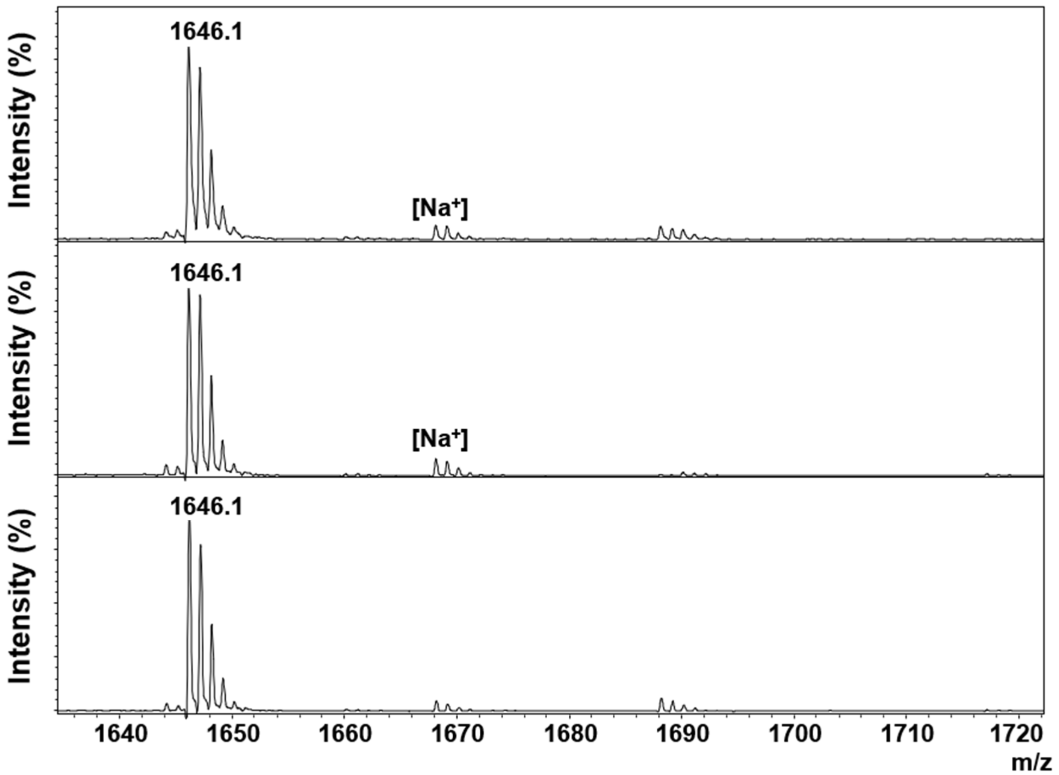

Figure S50. MALDI-TOF MS analysis of GCN5 (2 μM)-catalyzed acetylation reaction of H3h$_3$K9 peptide (100 μM) in the presence of AcCoA (300 μM) at 37 °C, after 3 h incubation (top panel). Control reactions after 3 h: absence of AcCoA (middle panel), absence of GCN5 (bottom panel).

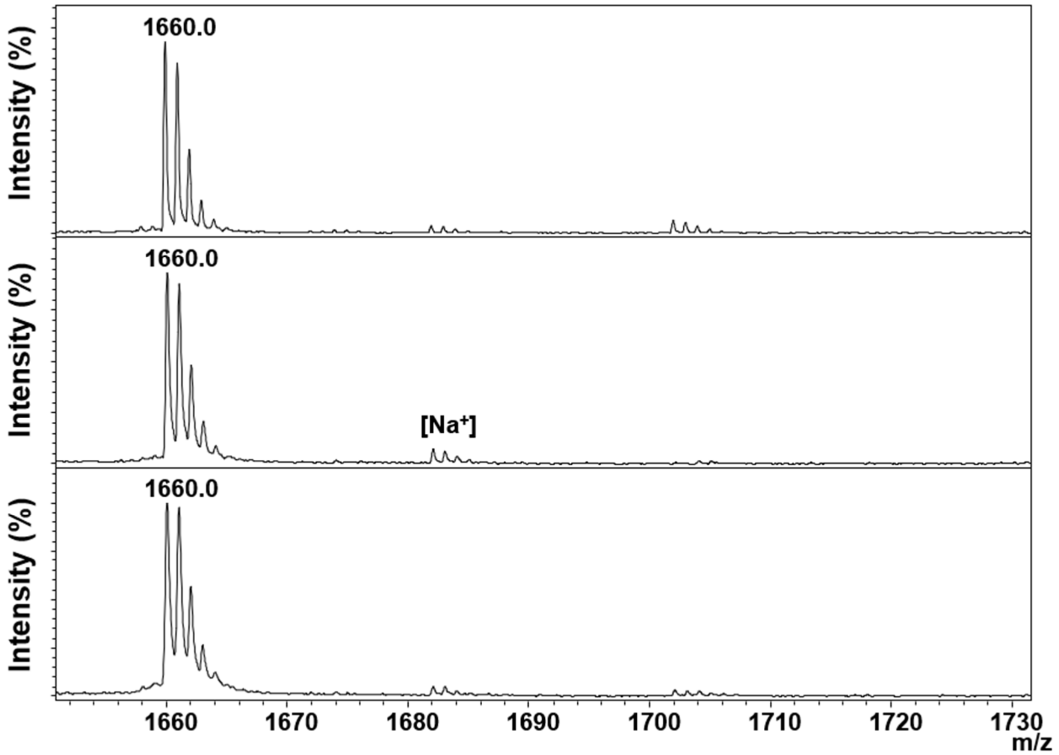

Figure S51. MALDI-TOF MS analysis of PCAF (2 μM)-catalyzed acetylation reaction of $H3h_4K9$ peptide (100 μM) in the presence of AcCoA (300 μM) at 37 °C, after 3 h incubation (top panel). Control reactions after 3 h: absence of AcCoA (middle panel), absence of PCAF (bottom panel).

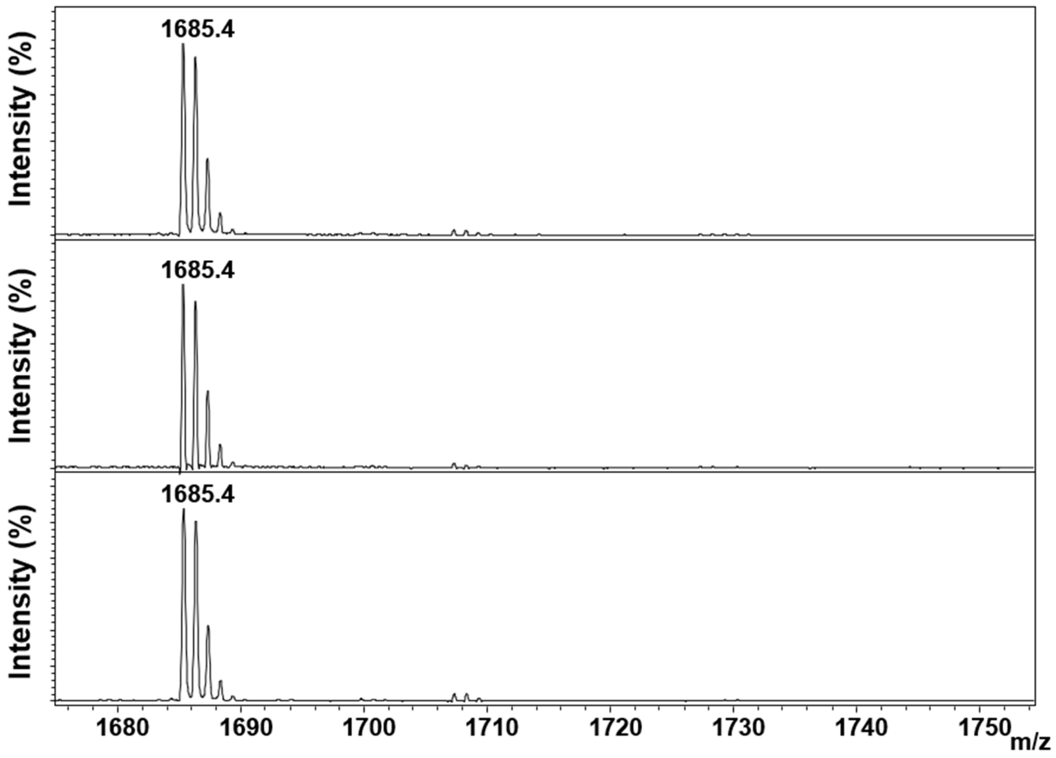

Figure S52. MALDI-TOF MS analysis of GCN5 (2 μM)-catalyzed acetylation reaction of $H3h_4K14$ peptide (100 μM) in the presence of AcCoA (300 μM) at 37 °C, after 3 h incubation (top panel). Control reactions after 3 h: absence of AcCoA (middle panel), absence of GCN5 (bottom panel).

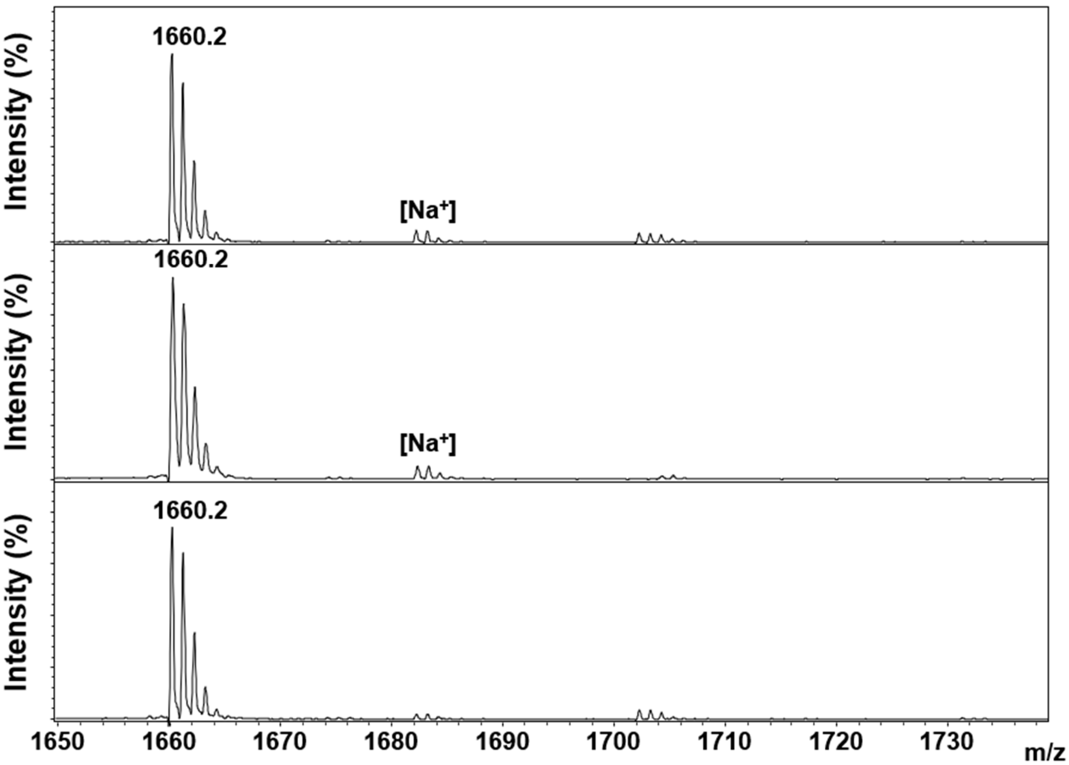

Figure S53. MALDI-TOF MS analysis of GCN5 (2 μM)-catalyzed acetylation reaction of H3h$_4$K9 peptide (100 μM) in the presence of AcCoA (300 μM) at 37 °C, after 3 h incubation (top panel). Control reactions after 3 h: absence of AcCoA (middle panel), absence of GCN5 (bottom panel).

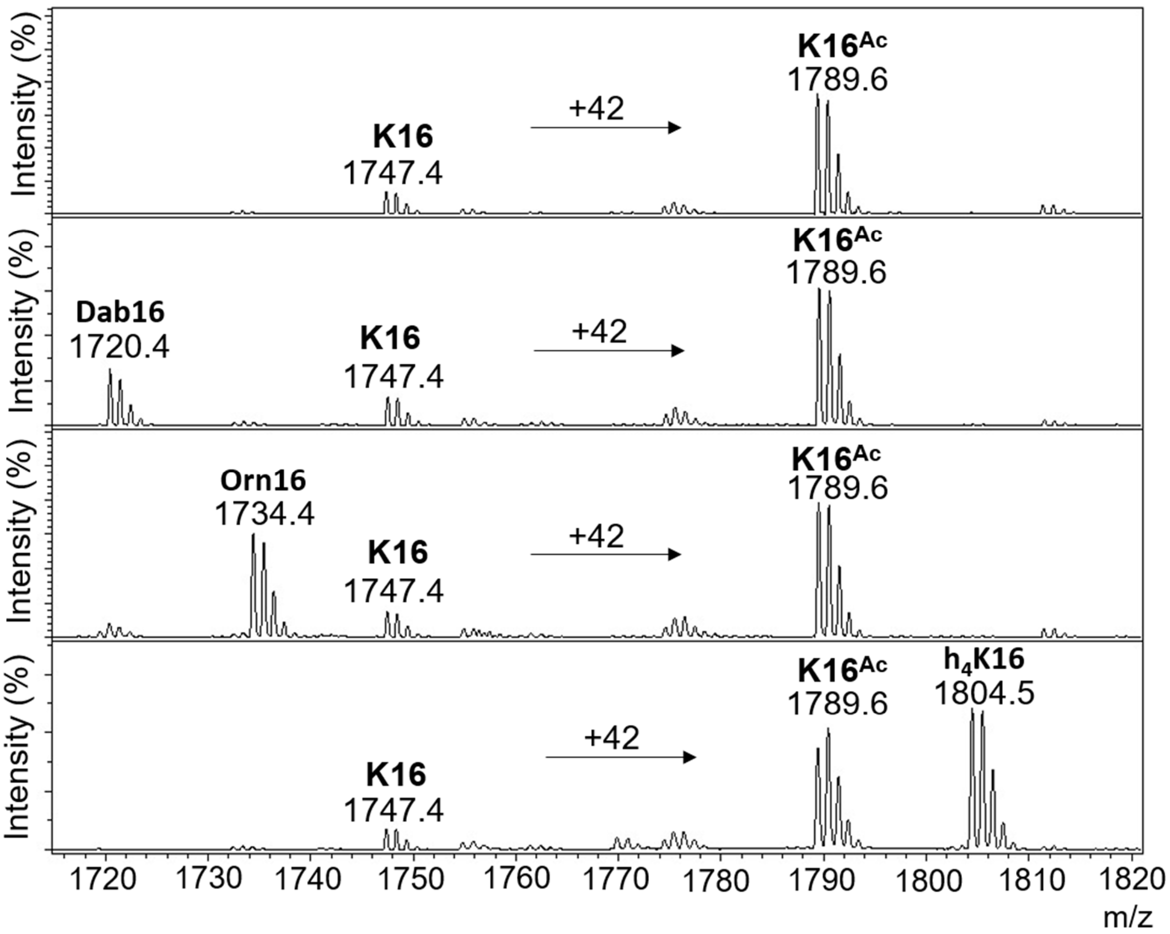

Figure S54. MALDI-TOF analysis of the competition experiment showing the MOF (0.2 µM)-catalyzed acetylation of H4K16 (100 µM) in presence, or not, of equimolar amount of H4X16 peptides (100 µM). Reactions were carried out at 37 °C and quenched after 1 h. From the top to the bottom: Control reaction; H4K16 + H4Dab16; H4K16 + H4Orn16; H4K16 + H4h_4K16.

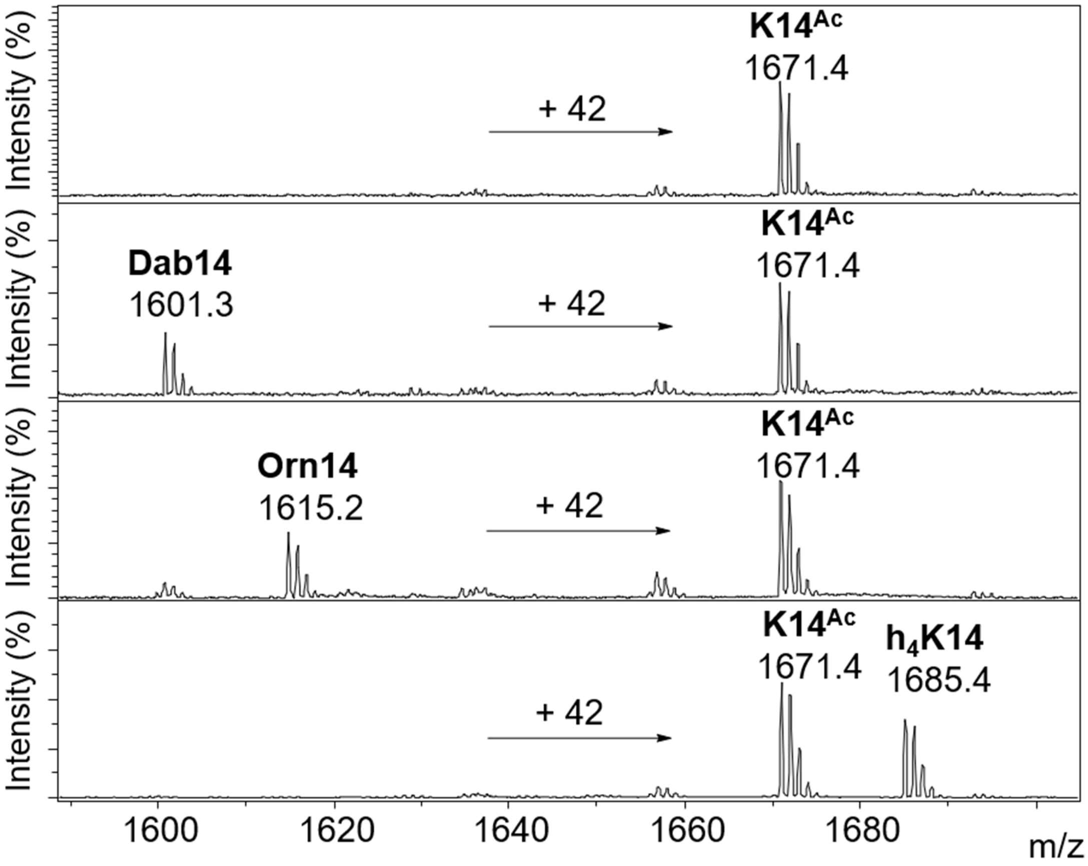

Figure S55. MALDI-TOF analysis of the competition experiment showing the PCAF (0.5 µM)-catalyzed acetylation of H3K14 (100 µM) in presence, or not, of equimolar amount of H3X14 peptides (100 µM). Reactions were carried out at 37 °C and quenched after 1 h. From the top to the bottom: Control reaction; H3K14 + H3Dab14; H3K14 + H3Orn14; H3K14 + H3h_4K14.

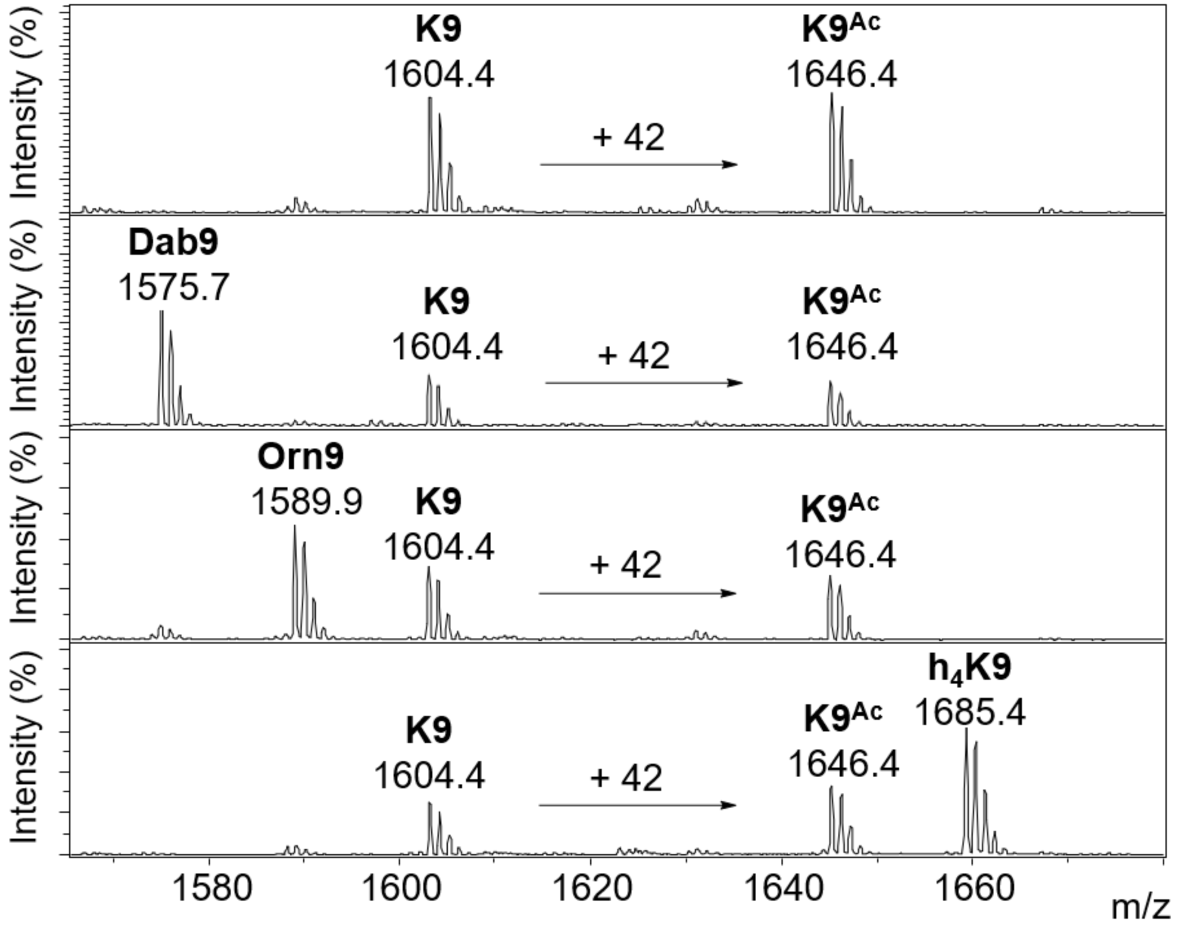

Figure S56. MALDI-TOF analysis of the competition experiment showing the PCAF (0.5 µM)-catalyzed acetylation of H3K9 (100 µM) in presence, or not, of equimolar amount of H3X9 peptides (100 µM). Reactions were carried out at 37 °C and quenched after 1 h. From the top to the bottom: Control reaction; H3K9 + H3Dab9; H3K9 + H3Orn9; H3K9 + H3h_4K9.

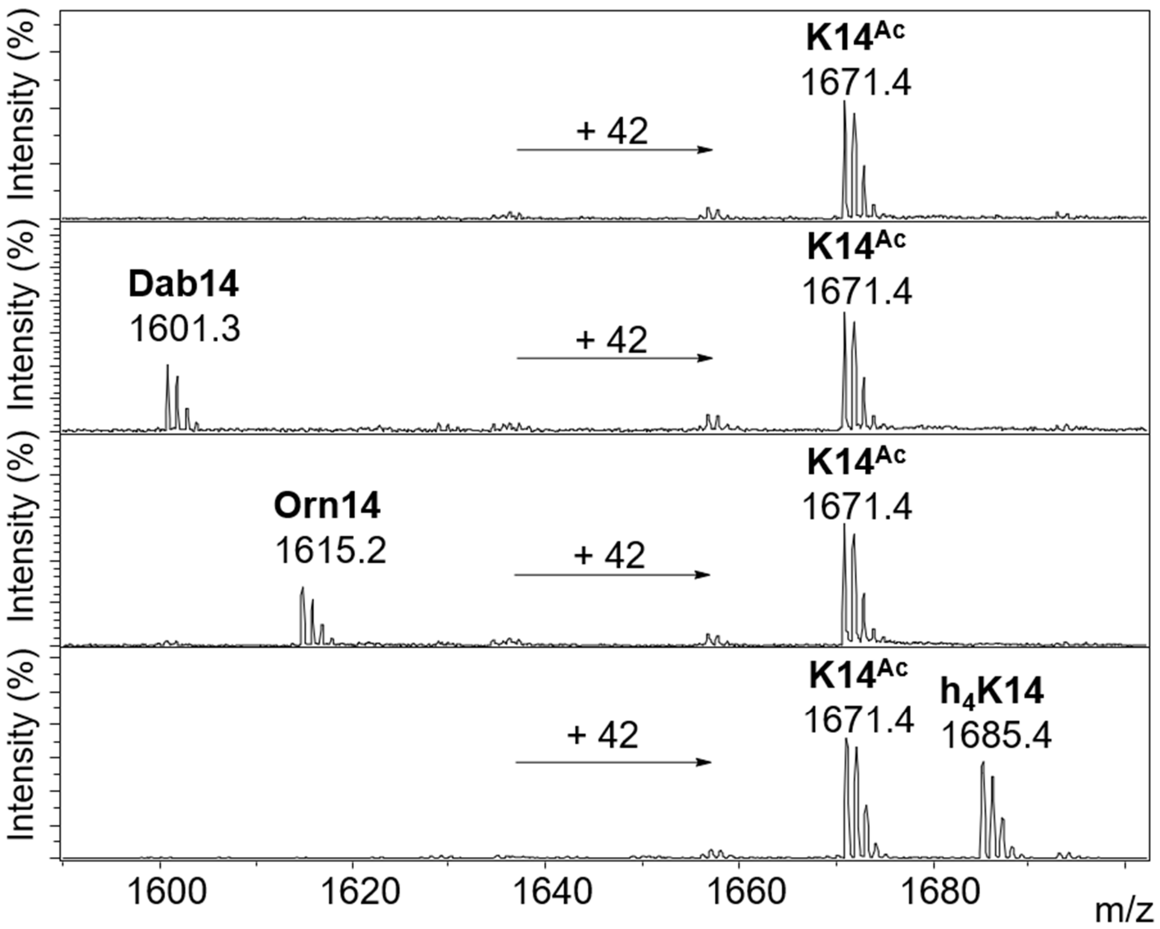

Figure S57. MALDI-TOF analysis of the competition experiment showing the GCN5 (0.5 µM)-catalyzed acetylation of H3K14 (100 µM) in presence, or not, of equimolar amount of H3X14 peptides (100 µM). Reactions were carried out at 37 °C and quenched after 1 h. From the top to the bottom: Control reaction; H3K14 + H3Dab14; H3K14 + H3Orn14; H3K14 + H3h$_4$K14.

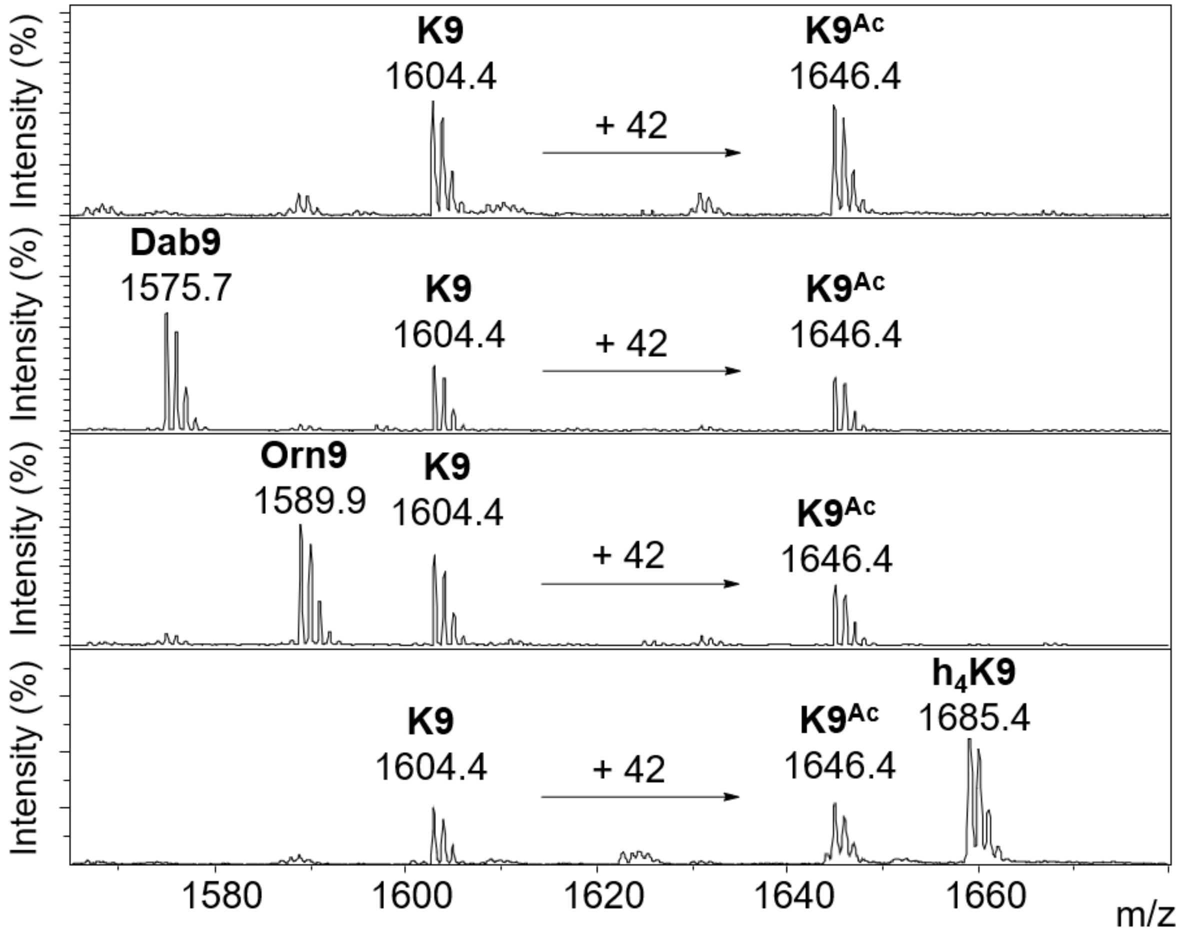

Figure S58. MALDI-TOF analysis of the competition experiment showing the GCN5 (0.5 µM)-catalyzed acetylation of H3K9 (100 µM) in presence, or not, of equimolar amount of H3X9 peptides (100 µM). Reactions were carried out at 37 °C and quenched after 1 h. From the top to the bottom: Control reaction; H3K9 + H3Dab9; H3K9 + H3Orn9; H3K9 + $H3h_4K9$.

8. Kinetics plots

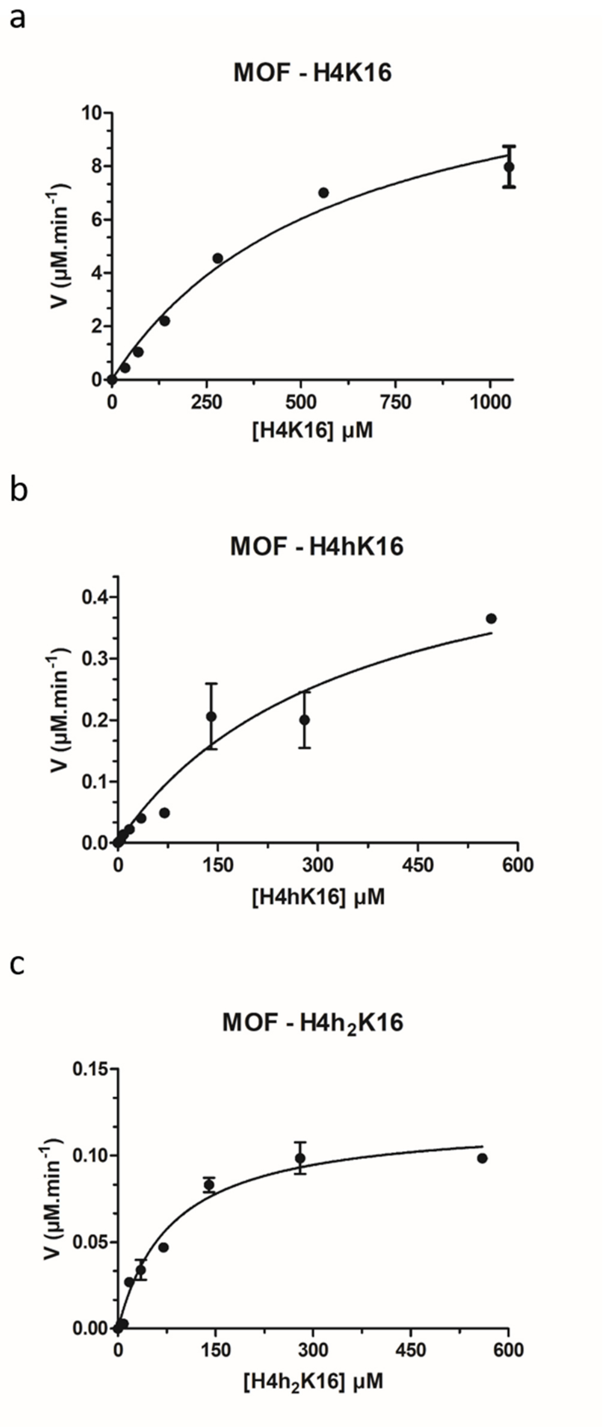

Figure S59. Michaelis-Menten plots of MOF-catalyzed acetylation of (a) H4K16; (b) H4hK16; (c) H4h$_2$K16. Experiments were carried out in replicates and data are reported as value ± SD.

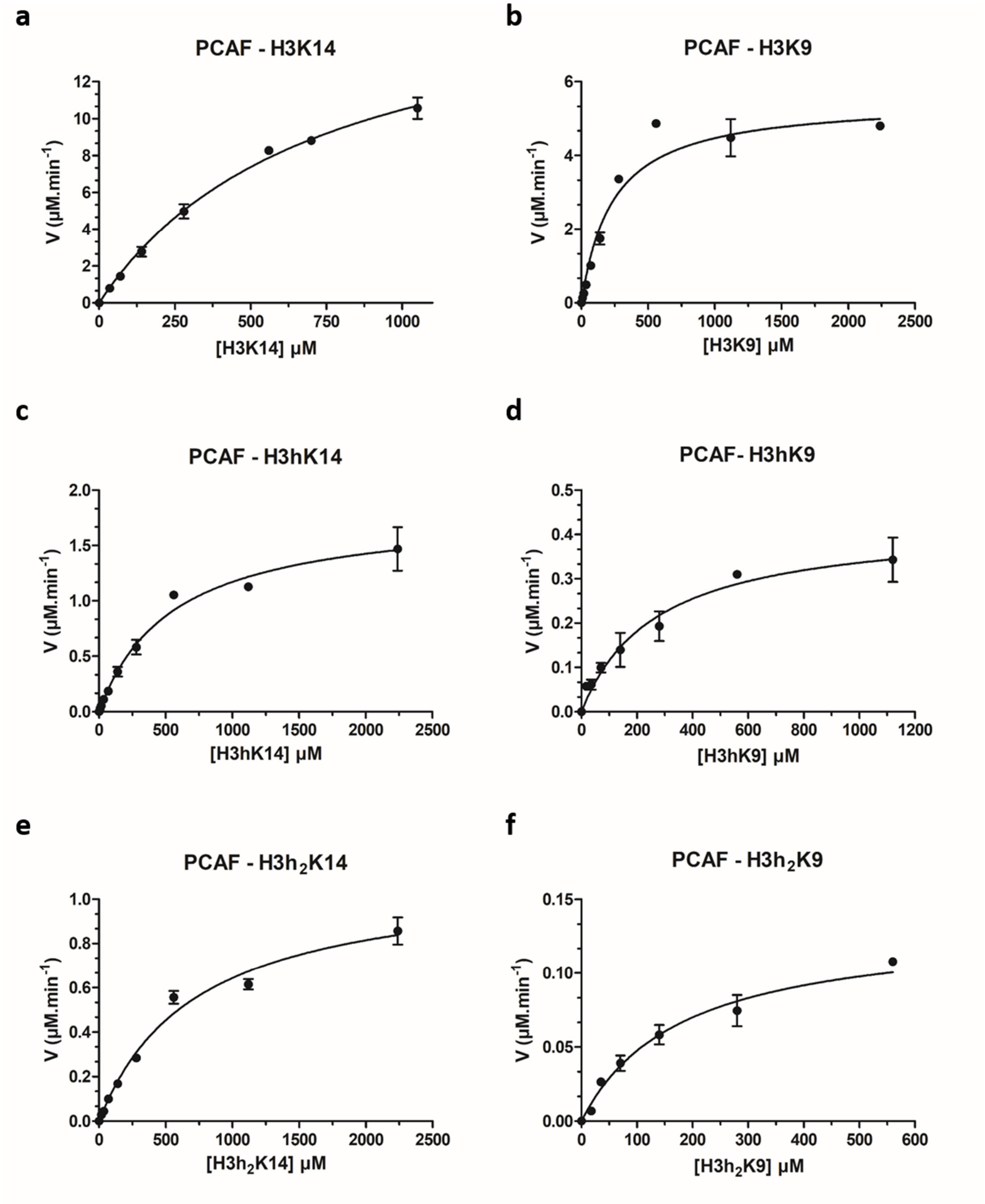

Figure S60. Michaelis-Menten plots of PCAF-catalyzed acetylation of (a) H4K14; (b) H3K9; (c) H3hK14; (d) H3hK9; (e) $H3h_2K14$; (f) $H3h_2K9$. Experiments were carried out in replicates and data are reported as value ± SD.

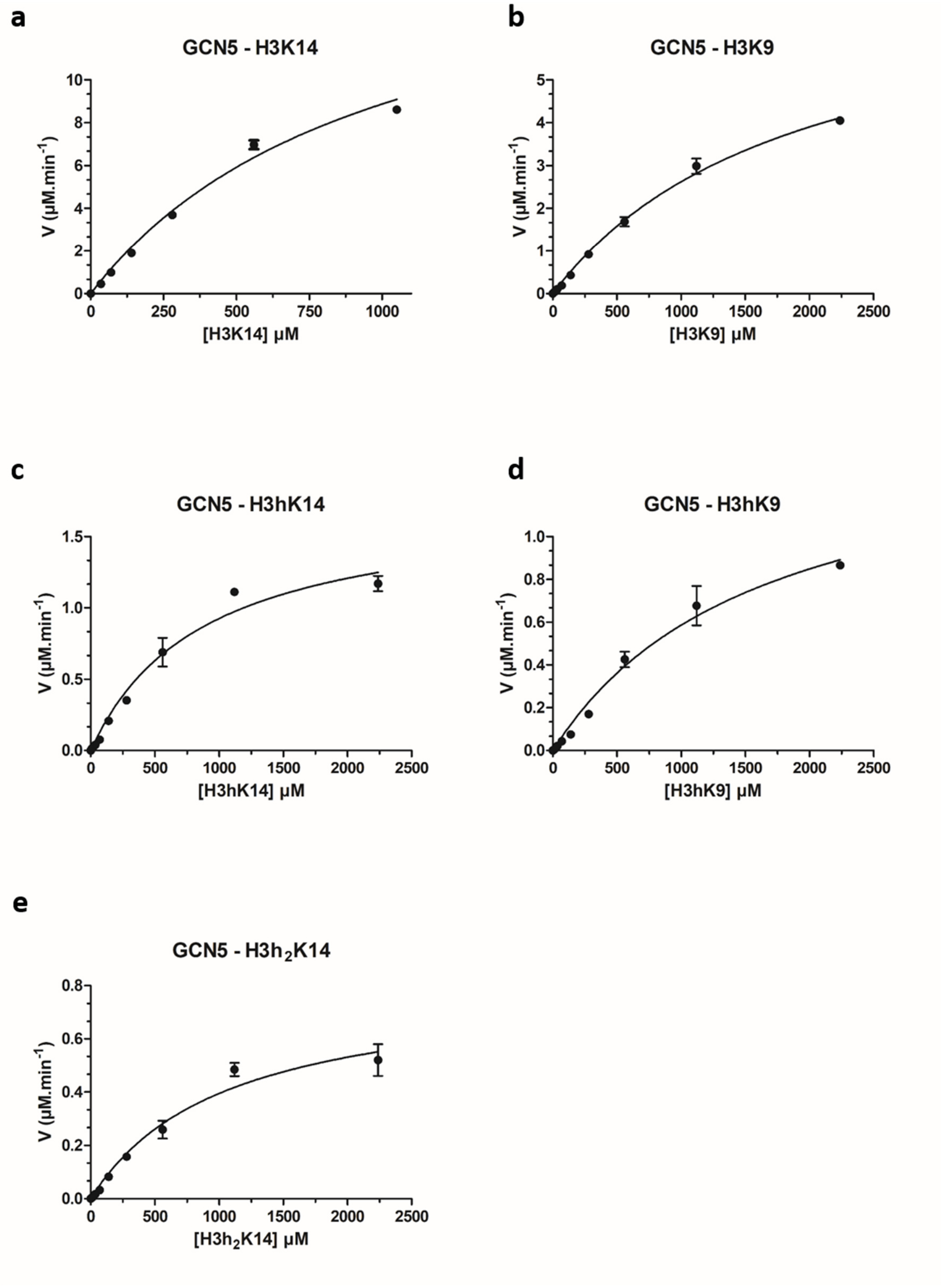

Figure S61. Michaelis-Menten plots of GCN5-catalyzed acetylation of (a) H4K14; (b) H3K9; (c) H3hK14; (d) H3hK9; (e) $H3h_2K14$. Experiments were carried out in replicates and data are reported as value ± SD.

9. NMR spectra

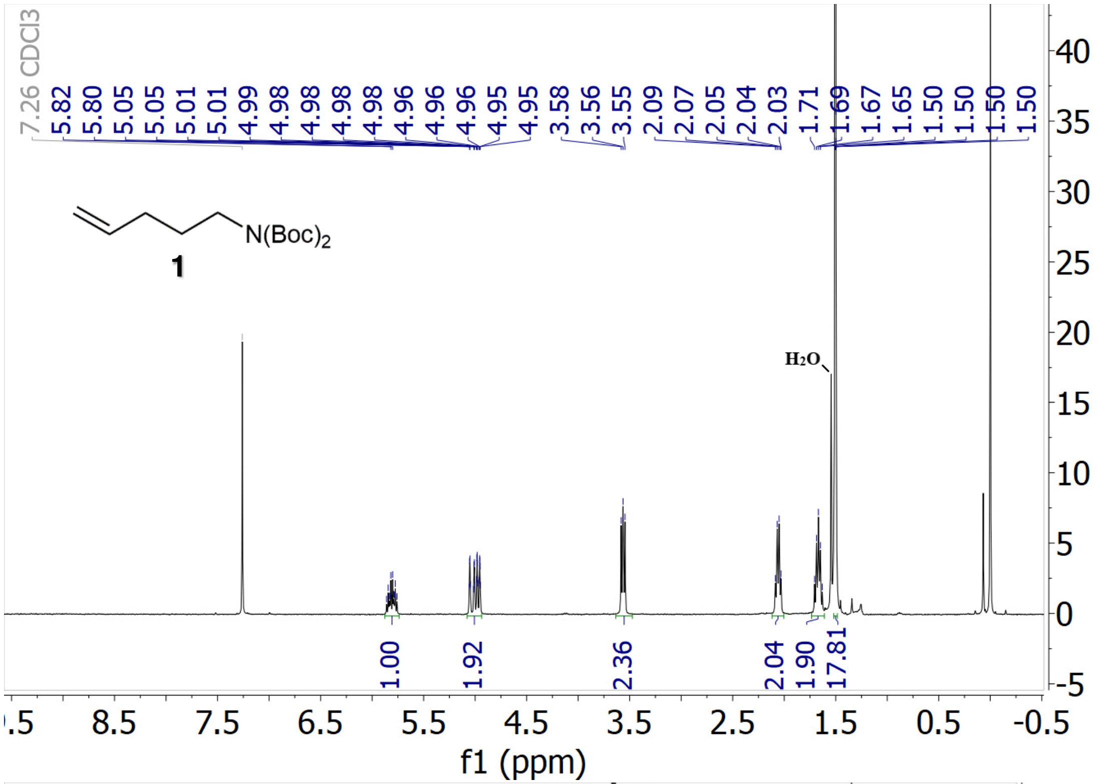

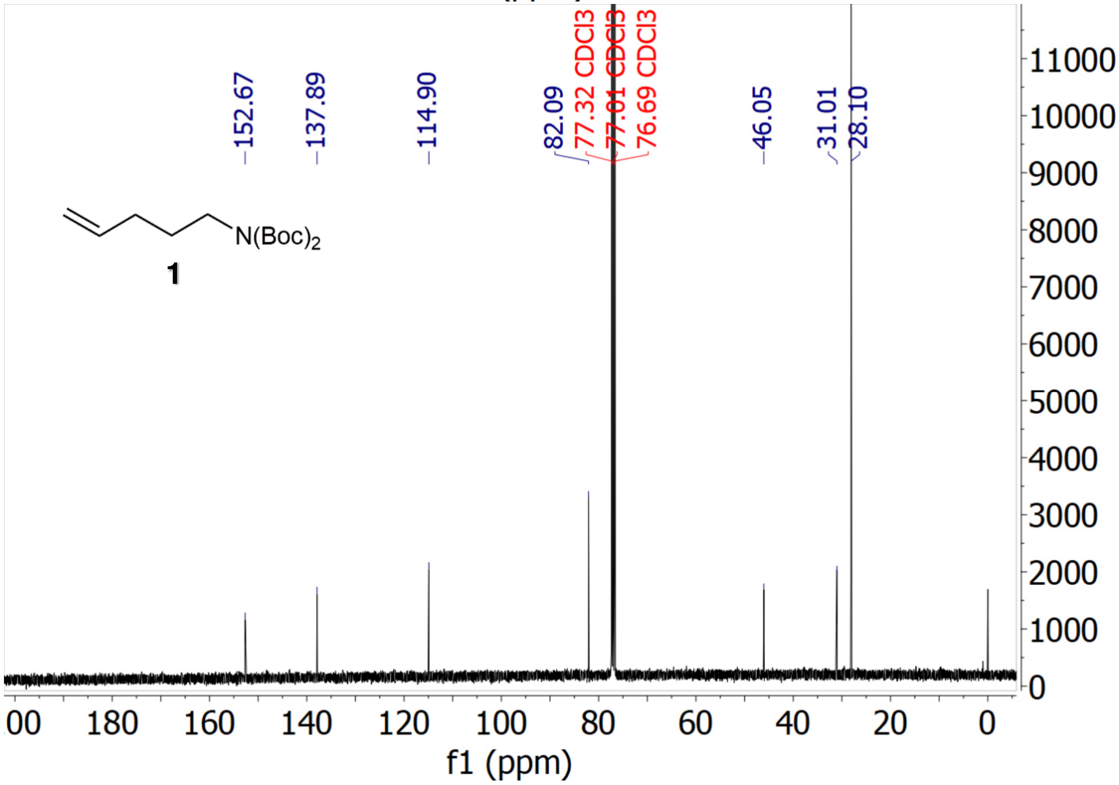

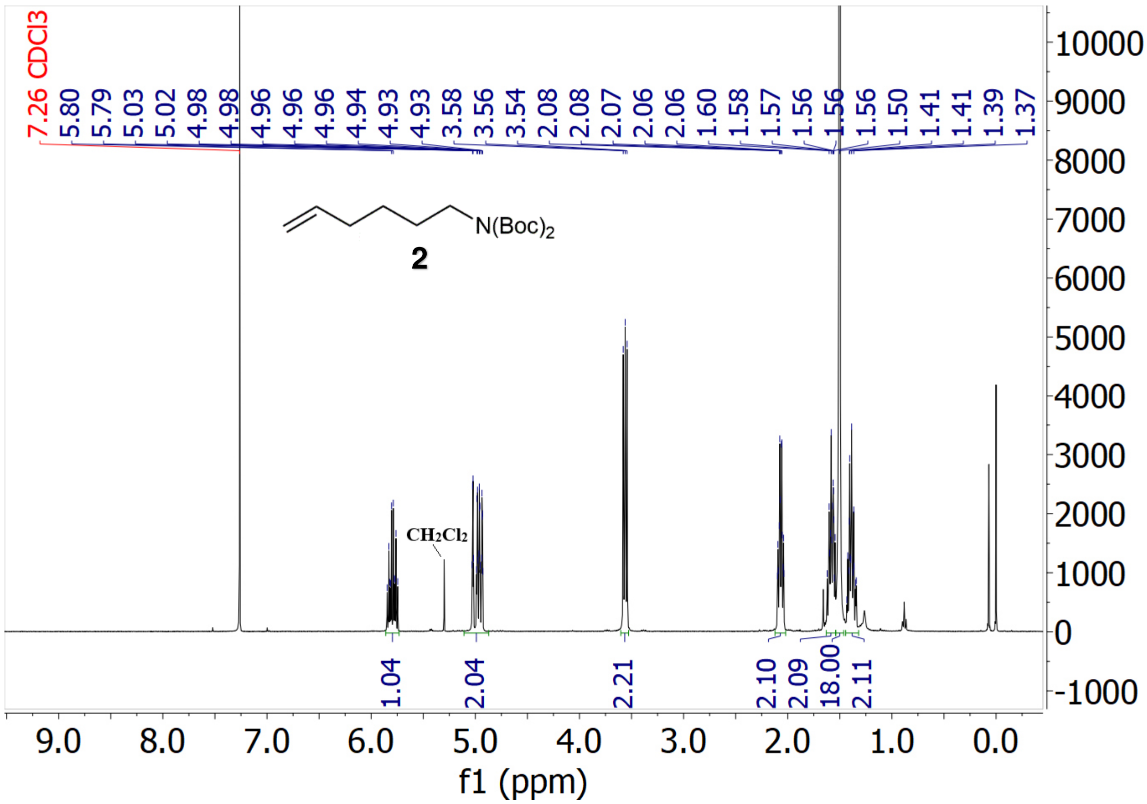

7.26 CDCl3
5.80
5.79
5.03
5.02
4.98
4.98
4.96
4.96
4.96
4.94
4.93
4.93
3.58
3.56
3.54
2.08
2.08
2.07
2.06
2.06
1.60
1.58
1.57
1.56
1.56
1.56
1.50
1.41
1.41
1.39
1.37
N(Boc)2
2
CH2Cl2
1.04
2.04
2.21
2.10
2.09
18.00
2.11
f1 (ppm)

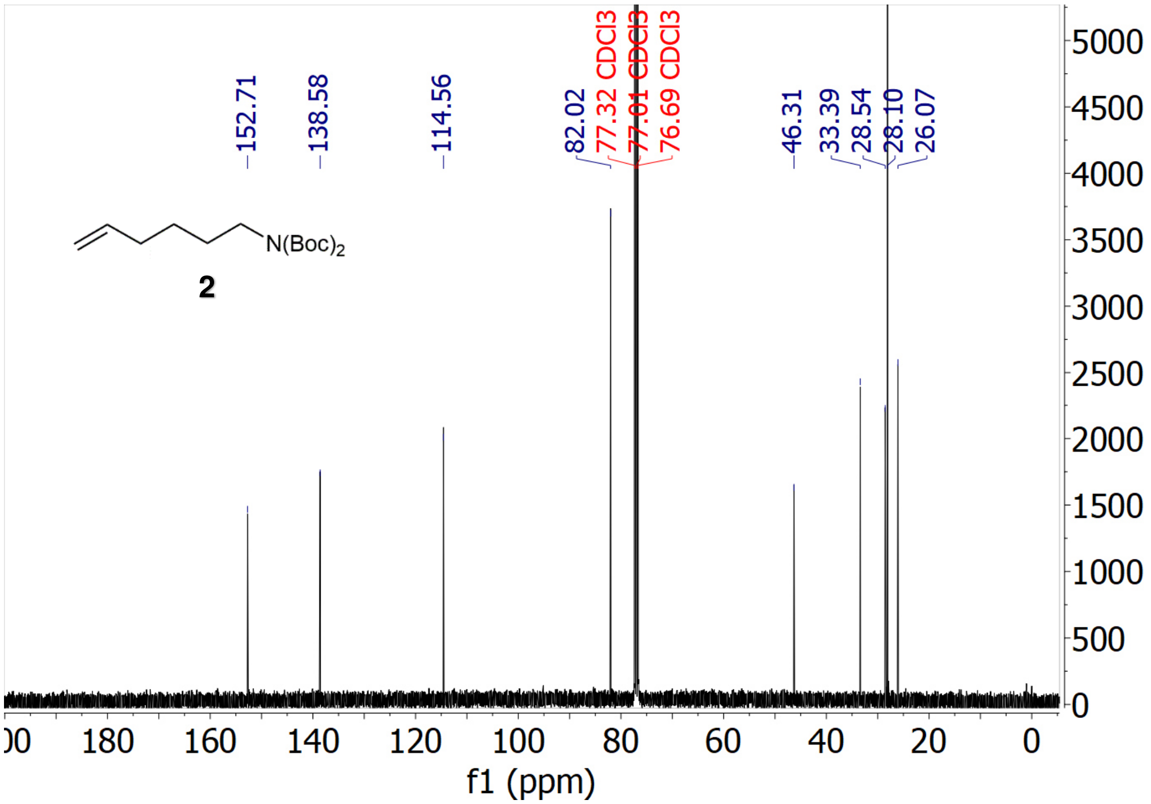

152.71
138.58
114.56
82.02
77.32 CDCl3
77.01 CDCl3
76.69 CDCl3
46.31
33.39
28.54
28.10
26.07
N(Boc)2
2
f1 (ppm)

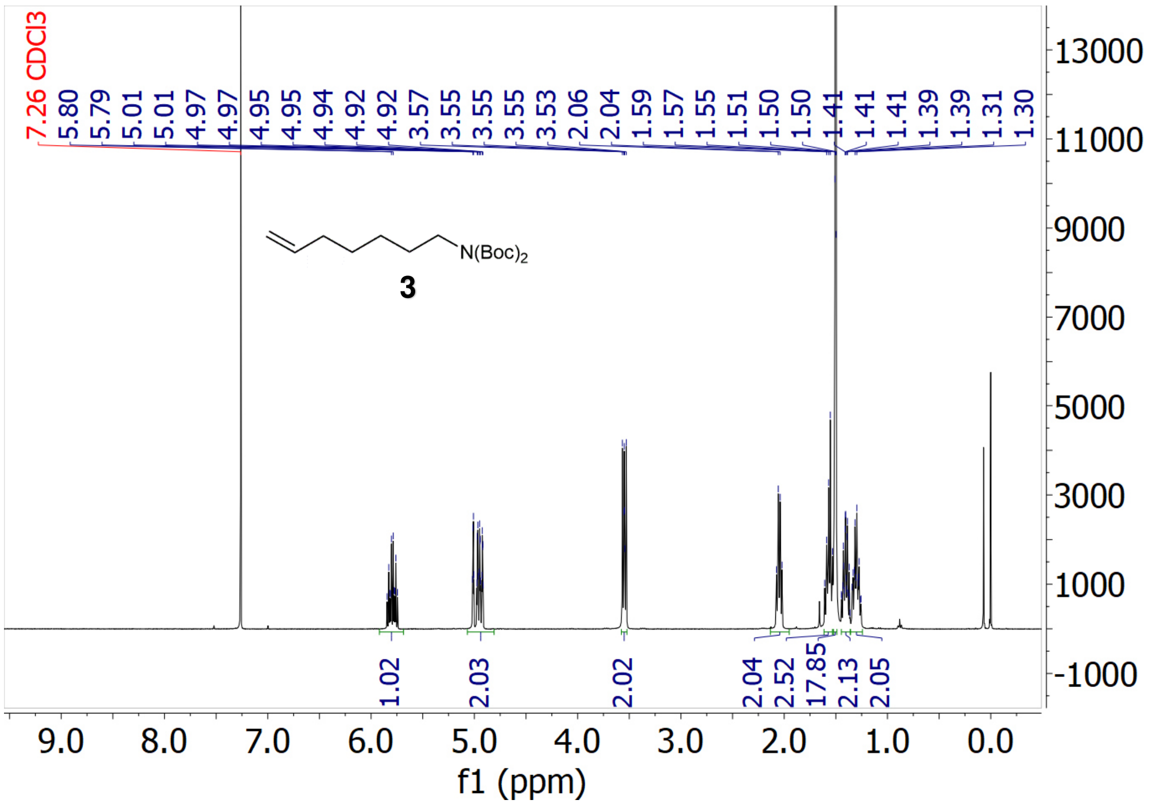
7.26 CDCl3
5.80
5.79
5.01
5.01
4.97
4.97
4.95
4.95
4.94
4.92
4.92
3.57
3.55
3.55
3.55
3.53
2.06
2.04
1.59
1.57
1.55
1.51
1.50
1.50
1.41
1.41
1.41
1.39
1.39
1.31
1.30
N(Boc)2
3
1.02
2.03
2.02
2.04
2.52
17.85
2.13
2.05
f1 (ppm)

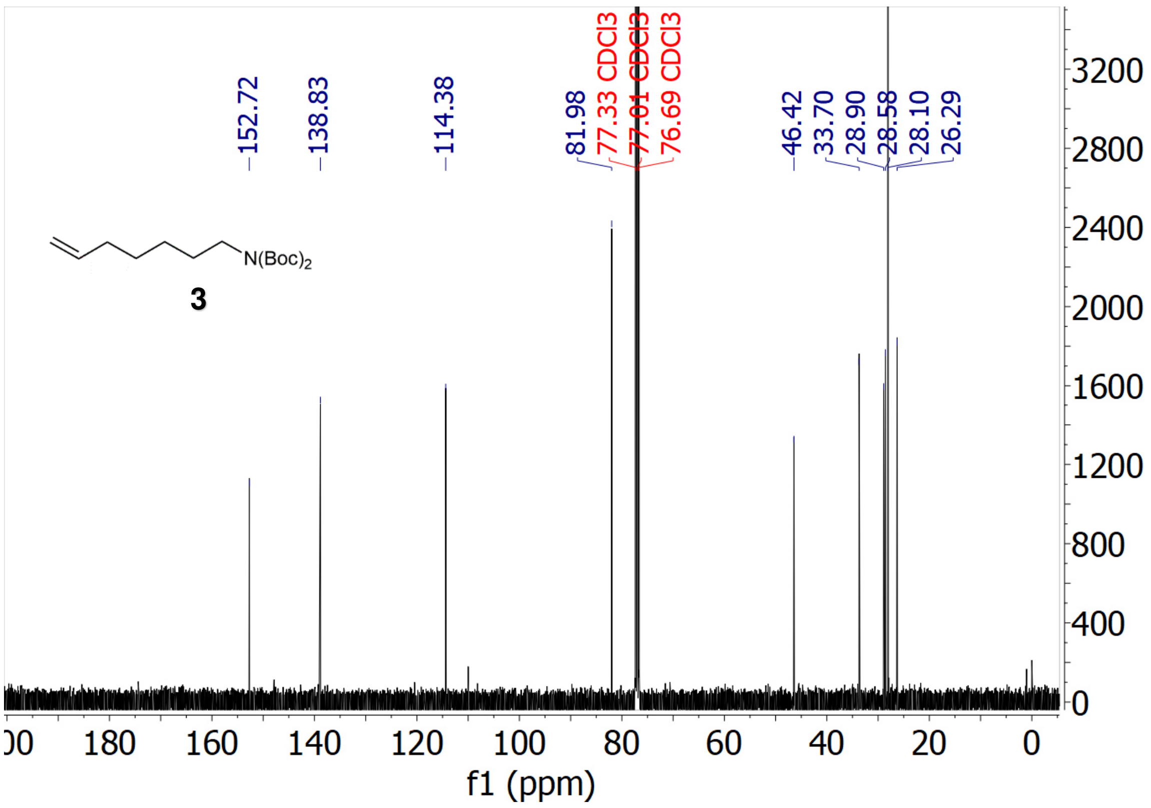
152.72
138.83
114.38
81.98
77.33 CDCl3
77.01 CDCl3
76.69 CDCl3
46.42
33.70
28.90
28.58
28.10
26.29
N(Boc)2
3
f1 (ppm)

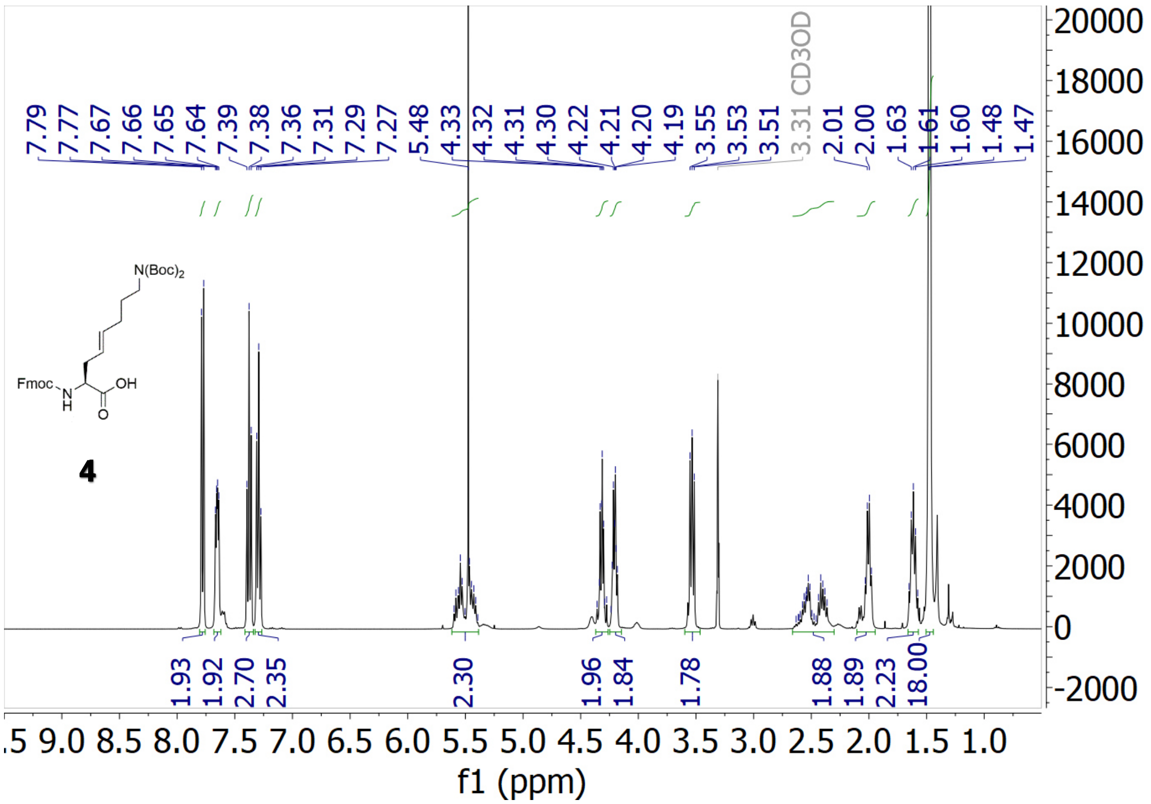

7.79 7.77 7.67 7.66 7.65 7.64 7.39 7.38 7.36 7.31 7.29 7.27 5.48 4.33 4.32 4.31 4.30 4.22 4.21 4.20 4.19 3.55 3.53 3.51 3.31 CD3OD 2.01 2.00 1.63 1.61 1.60 1.48 1.47
N(Boc)2
Fmoc
N
H
OH
O
4
1.93 1.92 2.70 2.35 2.30 1.96 1.84 1.78 1.88 1.89 2.23 18.00
20000 18000 16000 14000 12000 10000 8000 6000 4000 2000 0 -2000
.5 9.0 8.5 8.0 7.5 7.0 6.5 6.0 5.5 5.0 4.5 4.0 3.5 3.0 2.5 2.0 1.5 1.0
f1 (ppm)

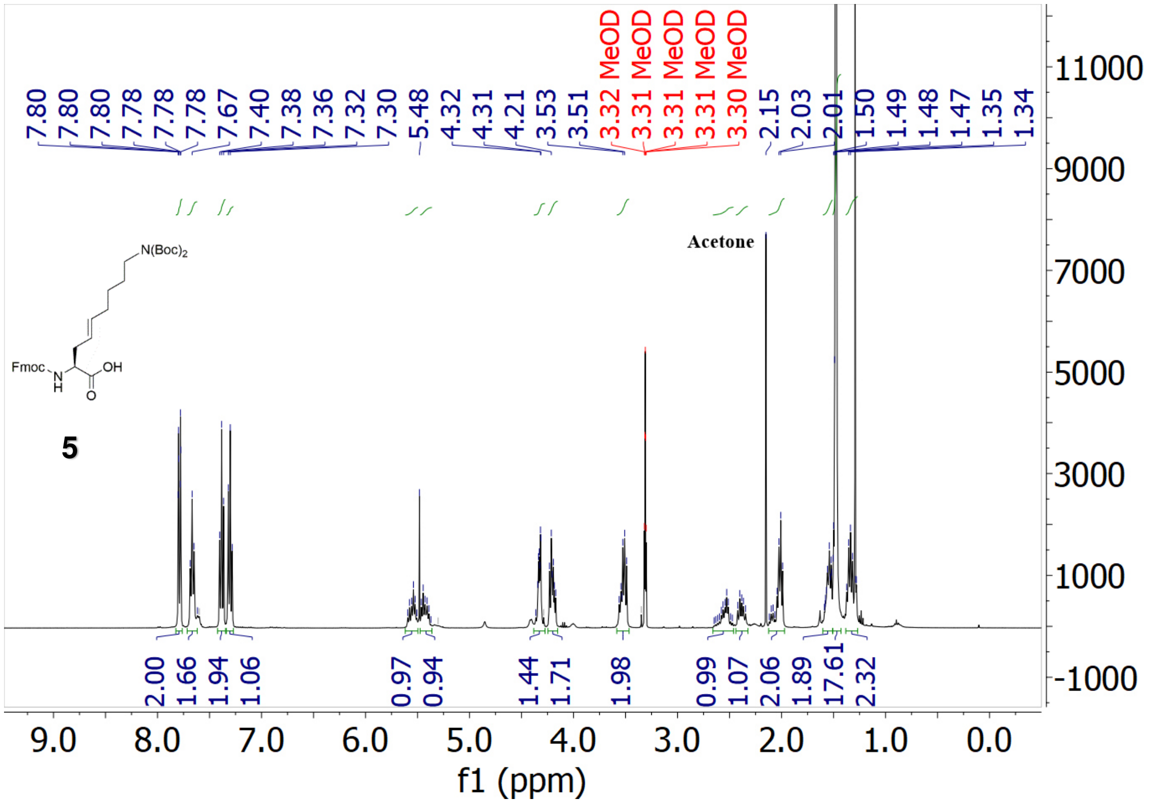

7.80 7.80 7.80 7.78 7.78 7.78 7.67 7.40 7.38 7.36 7.32 7.30 5.48 4.32 4.31 4.21 3.53 3.51 3.32 MeOD 3.31 MeOD 3.31 MeOD 3.31 MeOD 3.30 MeOD 2.15 2.03 2.01 1.50 1.49 1.48 1.47 1.35 1.34
Acetone
N(Boc)2
Fmoc
N
H
OH
O
5
2.00 1.66 1.94 1.06 0.97 0.94 1.44 1.71 1.98 0.99 1.07 2.06 1.89 17.61 2.32
11000 9000 7000 5000 3000 1000 -1000
9.0 8.0 7.0 6.0 5.0 4.0 3.0 2.0 1.0 0.0
f1 (ppm)

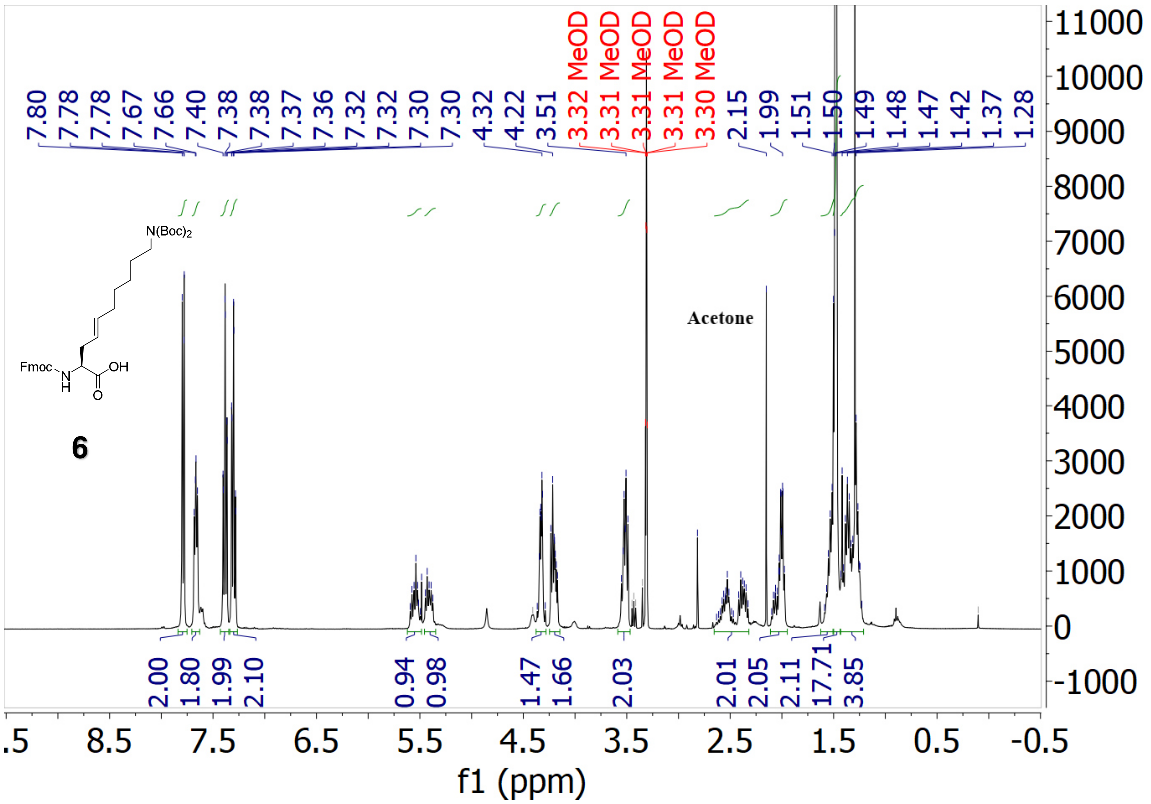

7.80 7.78 7.78 7.67 7.66 7.40 7.38 7.38 7.37 7.36 7.32 7.32 7.30 7.30 4.32 4.22 3.51
3.32 MeOD 3.31 MeOD 3.31 MeOD 3.31 MeOD 3.30 MeOD
2.15 1.99 1.51 1.50 1.49 1.48 1.47 1.42 1.37 1.28
N(Boc)2
Fmoc
N
H
OH
O
6
Acetone
2.00 1.80 1.99 2.10 0.94 0.98 1.47 1.66 2.03 2.01 2.05 2.11 17.71 3.85
11000 10000 9000 8000 7000 6000 5000 4000 3000 2000 1000 0 -1000
.5 8.5 7.5 6.5 5.5 4.5 3.5 2.5 1.5 0.5 -0.5
f1 (ppm)

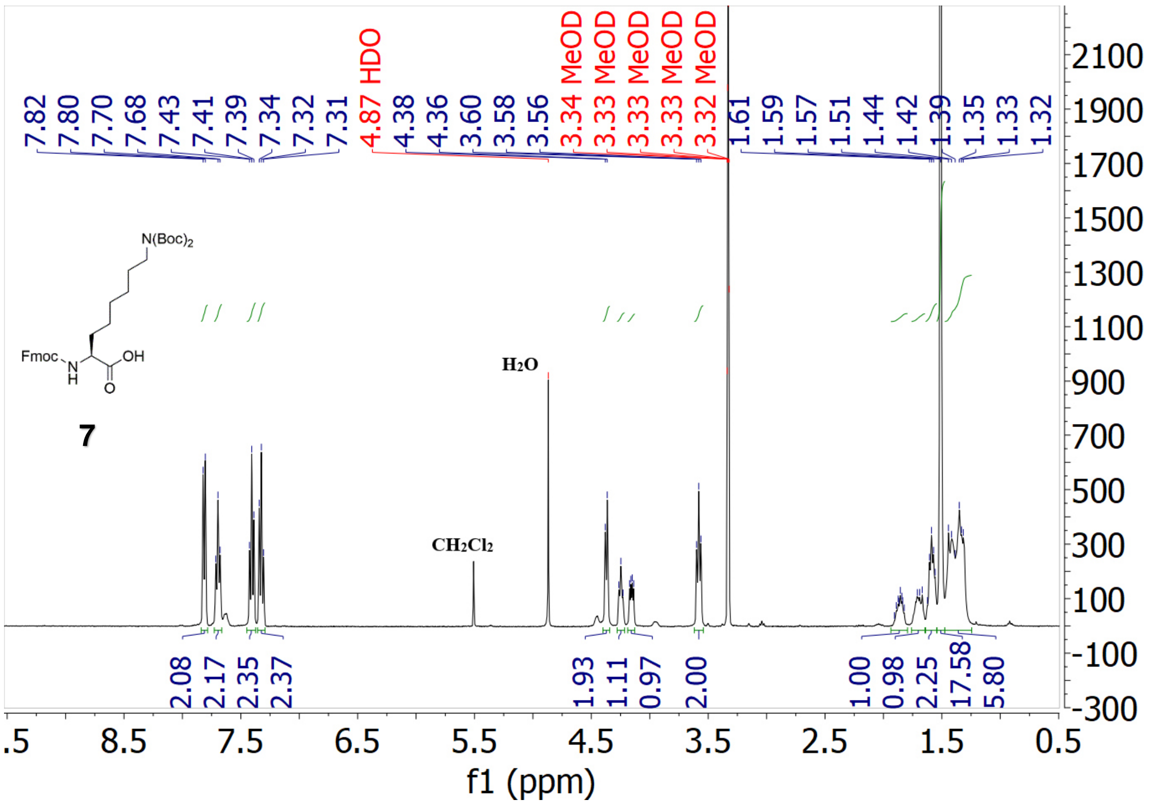
7.82
7.80
7.70
7.68
7.43
7.41
7.39
7.34
7.32
7.31
4.87 HDO
4.38
4.36
3.60
3.58
3.56
3.34 MeOD
3.33 MeOD
3.33 MeOD
3.33 MeOD
3.32 MeOD
1.61
1.59
1.57
1.51
1.44
1.42
1.39
1.35
1.33
1.32
N(Boc)2
Fmoc
N
H
OH
O
7
H2O
CH2Cl2
2.08
2.17
2.35
2.37
1.93
1.11
0.97
2.00
1.00
0.98
2.25
17.58
5.80
f1 (ppm)

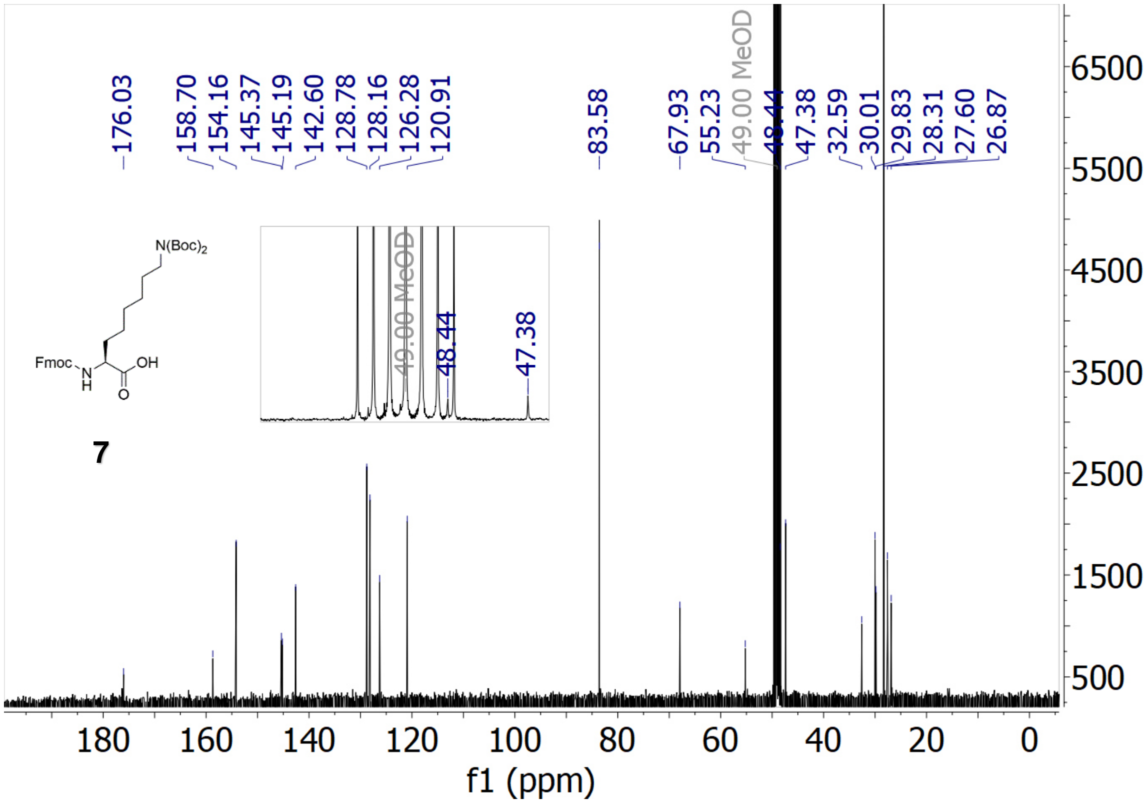
176.03
158.70
154.16
145.37
145.19
142.60
128.78
128.16
126.28
120.91
83.58
67.93
55.23
49.00 MeOD
48.44
47.38
32.59
30.01
29.83
28.31
27.60
26.87
N(Boc)2
Fmoc
N
H
OH
O
7
f1 (ppm)

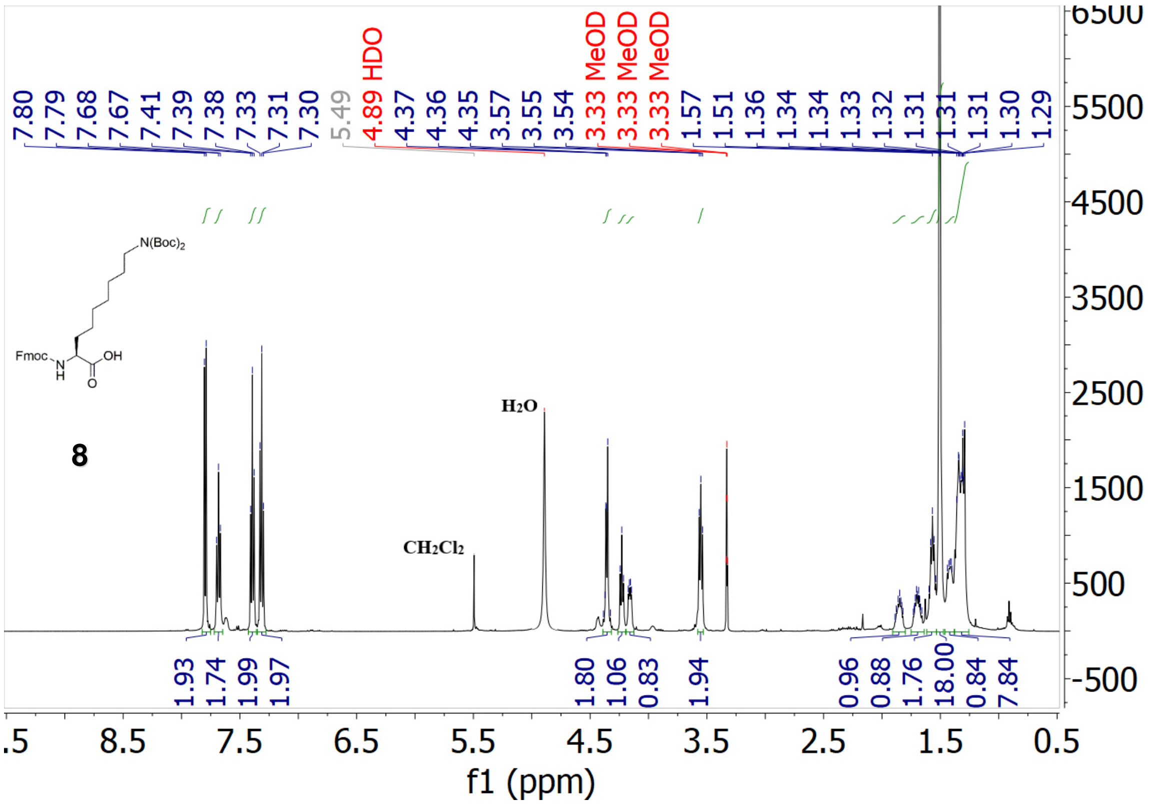
7.80
7.79
7.68
7.67
7.41
7.39
7.38
7.33
7.31
7.30
5.49
4.89 HDO
4.37
4.36
4.35
3.57
3.55
3.54
3.33 MeOD
3.33 MeOD
3.33 MeOD
1.57
1.51
1.36
1.34
1.34
1.33
1.32
1.31
1.31
1.31
1.30
1.29
N(Boc)2
Fmoc
N
H
OH
O
8
H2O
CH2Cl2
1.93
1.74
1.99
1.97
1.80
1.06
0.83
1.94
0.96
0.88
1.76
18.00
0.84
7.84
f1 (ppm)

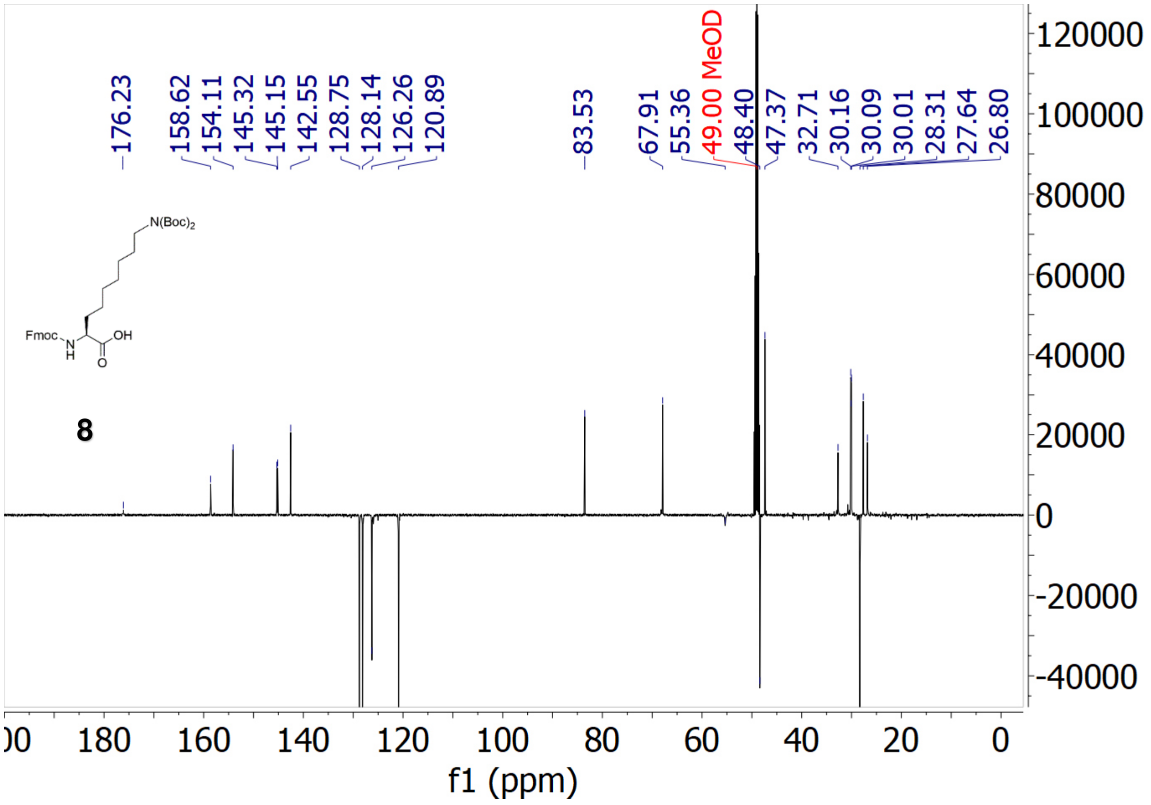
176.23
158.62
154.11
145.32
145.15
142.55
128.75
128.14
126.26
120.89
83.53
67.91
55.36
49.00 MeOD
48.40
47.37
32.71
30.16
30.09
30.01
28.31
27.64
26.80
N(Boc)2
Fmoc
N
H
OH
O
8
f1 (ppm)

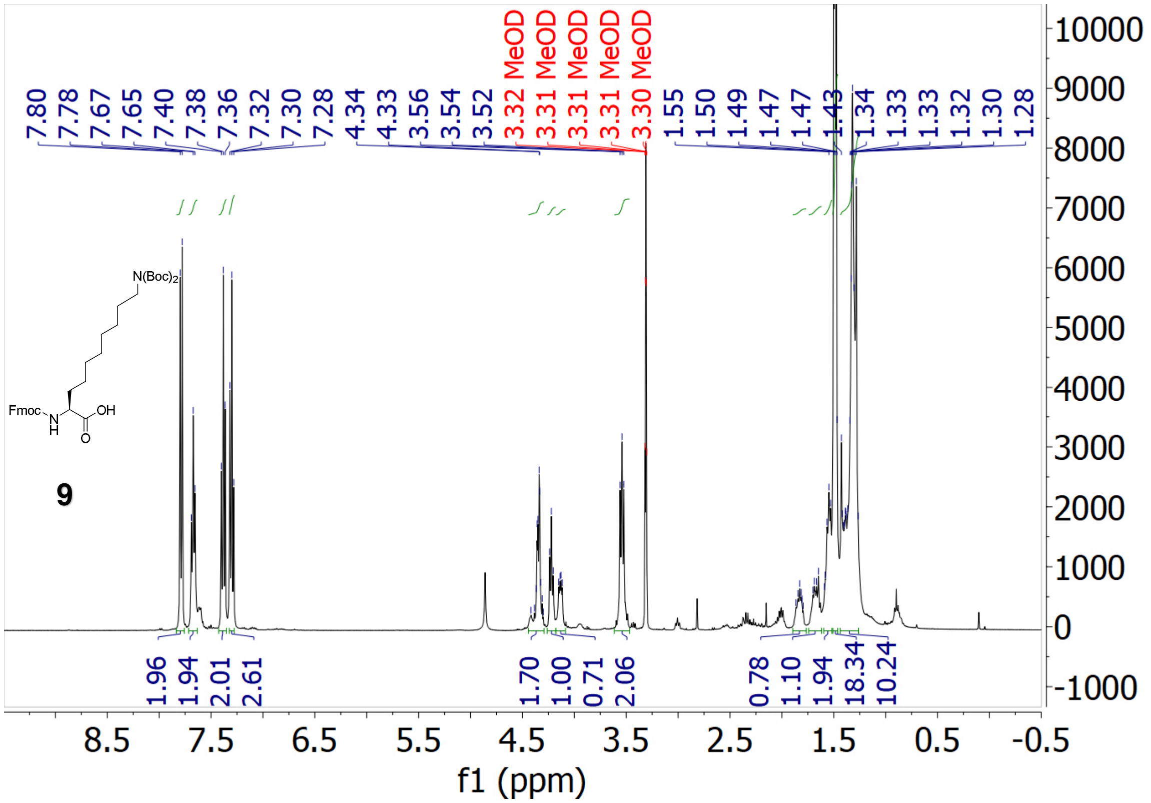
7.80 7.78 7.67 7.65 7.40 7.38 7.36 7.32 7.30 7.28 4.34 4.33 3.56 3.54 3.52 3.32 MeOD 3.31 MeOD 3.31 MeOD 3.31 MeOD 3.30 MeOD 1.55 1.50 1.49 1.47 1.47 1.43 1.34 1.33 1.33 1.32 1.30 1.28
N(Boc)2
Fmoc
N
H
OH
O
9
1.96 1.94 2.01 2.61 1.70 1.00 0.71 2.06 0.78 1.10 1.94 18.34 10.24
f1 (ppm)

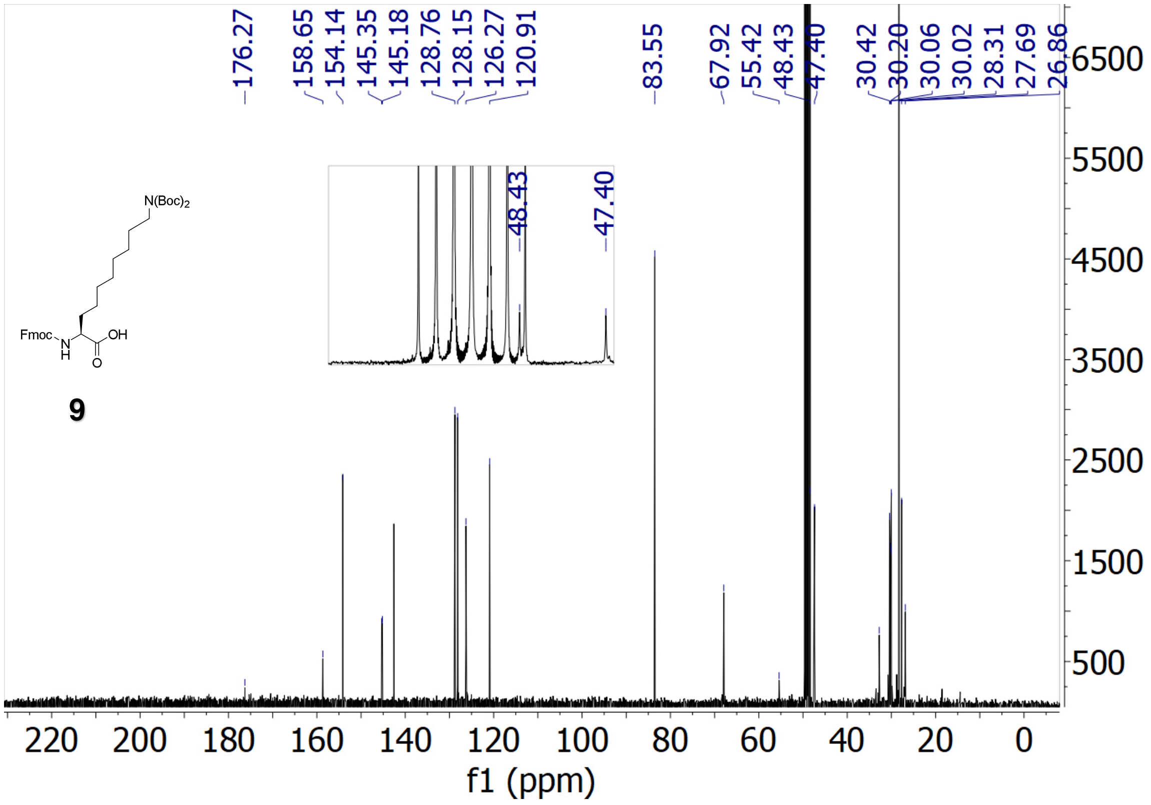
176.27 158.65 154.14 145.35 145.18 128.76 128.15 126.27 120.91 83.55 67.92 55.42 48.43 47.40 30.42 30.20 30.06 30.02 28.31 27.69 26.86
48.43
47.40
N(Boc)2
Fmoc
N
H
OH
O
9
f1 (ppm)

10. References

1. Cox, N., Dang, H., Whittaker, A. M. & Lalic, G. NHC-copper hydrides as chemoselective reducing agents: Catalytic reduction of alkynes, alkyl triflates, and alkyl halides. *Tetrahedron* (2014).

2. Smith, M. E. B. *et al.* Development of chemical probes: Toward the mode of action of a methylene-linked di(aryl acetate) E1. *Bioorg. Med. Chem.* (2010).
